# Cytotoxic polyhydroxylated pregnane glycosides from *Cissampelos pareira* var. *hirsuta*†

**DOI:** 10.1039/d1ra07498a

**Published:** 2021-12-22

**Authors:** Yan-Jun Sun, Hao-Jie Chen, Rui-Jie Han, Chen Zhao, Ying-Ying Si, Meng Li, Kun Du, Hui Chen, Wei-Sheng Feng

**Affiliations:** Co-construction Collaborative Innovation Center for Chinese Medicine, Respiratory Diseases by Henan & Education Ministry of P. R. China, Henan University of Chinese Medicine Zhengzhou 450046 P. R. China sunyanjunily@126.com fwsh@hactcm.edu.cn; School of Pharmacy, Henan University of Chinese Medicine Zhengzhou 450046 P. R. China; Henan Research Center for Special Processing Technology of Chinese Medicine Zhengzhou 450046 P. R. China

## Abstract

Fourteen new polyhydroxylated pregnane glycosides, cissasteroid A–N (1–14), and five known analogues (15–19), were isolated from the dried whole plant of *Cissampelos pareira* var. *hirsuta*. Their structures and stereochemistry were elucidated by extensive spectroscopic data, chemical hydrolysis, and ECD measurements. All the compounds were tested for their cytotoxicity against five human cancer cell lines, and inhibitory activity against NO release in LPS-induced RAW 264.7 cells. Compared with cisplatin, compound 7 showed more potent cytotoxicities against the HL-60, A549, SMMC-7721, MCF-7, and SW480 cell lines, with IC_50_ values of 2.19, 14.38, 2.00, 7.58, and 7.44 μM, respectively. The preliminary study of structure–activity relationship indicated that benzoic acid esterification at C-20 may have a negative effect on the cytotoxic activity of polyhydroxylated pregnane derivatives in these five human cancer cell lines. These results revealed the potential of compound 7 as an ideal antitumor lead compound.

## Abstract 2

Fourteen new polyhydroxylated pregnane glycosides, cissasteroid A–N (1–14), and five known analogues (15–19), were isolated from the dried whole plant of *Cissampelos pareira* var. *hirsuta*.

## Graphical abstract

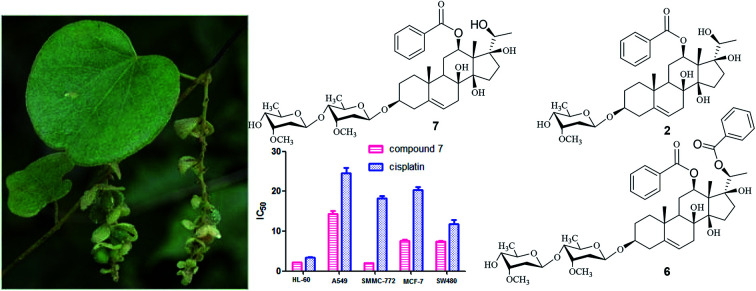

## Introduction

**Fig. 1:**
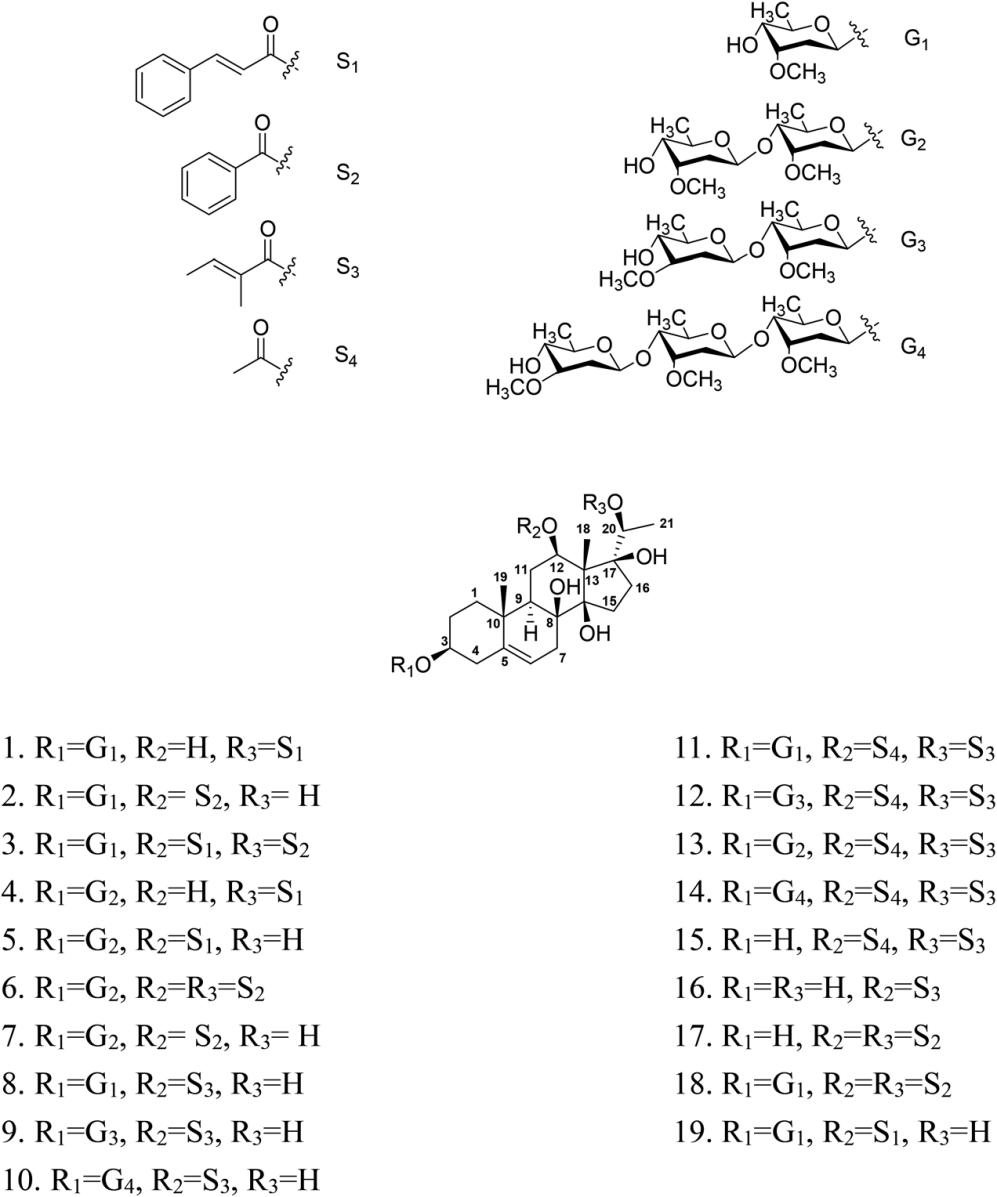
Structures of compounds 1–19.

Pregnane glycosides are an important class of secondary metabolites in the plant kingdom. Previous pharmacological investigations have demonstrated various kinds of bioactivities, such as immunosuppressive, anti-oxidant,^1^ anti-inflammatory,^2^ anti-epileptic,^3^ neuroprotective,^4^ anti-diabetic,^5^ anti-proliferative,^6^ anti-obesitic,^7^ and gastroprotective properties.^8^ The genus *Cissampelos* (Menispermaceae) is composed of 21 species, distributed in the Southwest of China, India, Malaysia, Pakistan, America and East Africa.^9,10^ The plants of this genus are used for the treatment of indolent ulcer, asthma, cholera, diarrhea, dysentery, epilepsy, fever, rabies,^9^ malaria,^10^ abdominal pain, inflammation, indigestion, wound healing, skin disorders, and snake venom.^11^ More than 60 natural products have been previously obtained, including alkaloids,^12^ flavonoids,^11^ and terpenes.^13^ Listed in *Chinese Pharmacopoeia*, the whole plant of *C. pareira* var. *Hirsuta* has been used clinically for trauma pain and bleeding as a traditional Dai medicine. In a search for bioactive natural products from traditional Chinese medicines, fourteen new polyhydroxylated pregnane glycosides, cissasteroid A–N (1–14), and five known analogues (15–19), were isolated from the dried whole plant of *C. pareira* var. *hirsuta* (Fig. 1). Detailed isolation, structure elucidation and biological assessment of those isolates are reported herein.

## Results and discussion

**Fig. 2:**
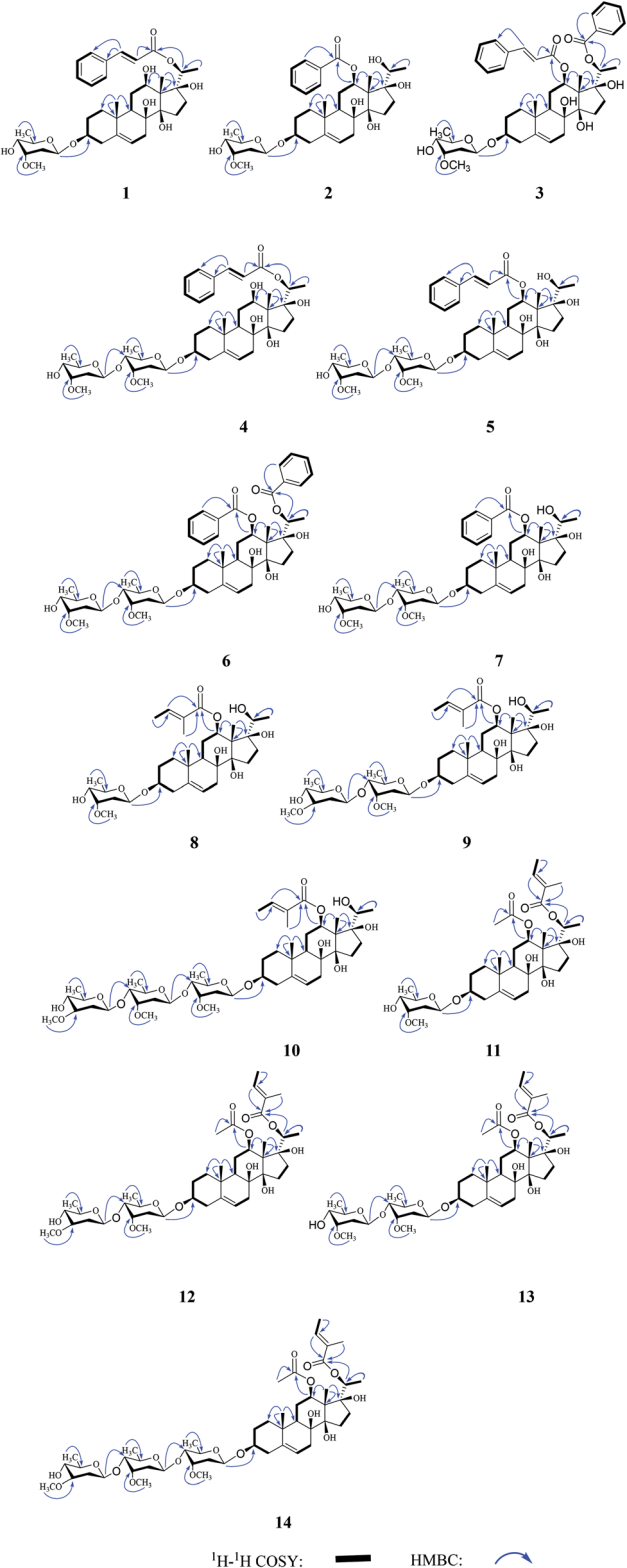
Key ^1^H–^1^H COSY and HMBC correlations of compounds 1–14.

Compound 1 was obtained as a white amorphous powder and possessed a molecular formula C_37_H_52_O_10_ with twelve degrees of unsaturation, as revealed from its HR-ESI-MS analysis (*m*/*z* 679.3451 [M + Na]^+^, calcd 679.3458). The IR spectrum displayed the presence of aromatic ring (1639, 1451 cm^−1^), conjugated carbonyl (1703 cm^−1^), hydroxyl (3398 cm^−1^), and ether (1029 cm^−1^) functionalities. The ^13^C-NMR and DEPT spectra showed thirty-seven carbon signals, including eight quaternary carbons [two olefinic/aromatic, *δ*_C_ 140.3 (C-5), 136.1 (C-1′), one ester carbonyl *δ*_C_ 167.7 (C-9′), three oxygenated *δ*_C_ 75.1 (C-8), 89.5 (C-14), 88.9 (C-17)], sixteen methines [eight oxygenated *δ*_C_ 79.3 (C-3), 75.1 (C-8), 71.3 (C-12), 76.3 (C-20), 97.2 (C-1′′), 79.2 (C-3′′), 74.5 (C-4′′), 71.4 (C-5′′), eight olefinic/aromatic *δ*_C_ 120.1 (C-6), 129.1 (×2) (C-2′, 6′), 130.0 (×2) (C-3′, 5′), 131.3 (C-4′), 120.0 (C-7′), 145.7 (C-8′)], eight methylenes and five methyls (one oxygenated *δ*_C_ 58.1, C-7′′). The ^1^H and ^13^C-NMR spectra (Tables 1 and 2) revealed the presence of one cinnamoyl group, one *O*-methylated 2,6-dideoxysugar and one pregnanehexaol skeleton.^14^ One monosubstituted benzene ring *δ*_H_ 7.59 (2H, m, H-2′, 6′), 7.40 (3H, m, H-3′, 4′, 5′), one set of *trans* conjugated olefinic protons *δ*_H_ 7.74 (1H, d, *J* = 16.1 Hz, H-7′), 6.52 (1H, d, *J* = 16.1 Hz, H-8′), one ester carbonyl *δ*_C_ 167.7 (C-9′) were observed, suggesting the occurence of *trans*-cinnamoyl group.^14^ The cymaropyranosyl group was based on a series of signals consisting of one methylene group *δ*_H_ 2.13 (1H, m, H-2′′), 1.51 (1H, m, H-2′′), *δ*_C_ 35.9 (C-2′′), one methoxy group *δ*_H_ 3.43 (3H, s, H-7′′), *δ*_C_ 58.1 (C-7′′), one secondary methyl group *δ*_H_ 1.21 (3H, d, *J* = 6.3 Hz, H-6′′), *δ*_C_ 18.6 (C-6′′), three oxygenated aliphatic carbons *δ*_C_ 79.2 (C-3′′), 74.5 (C-4′′), 71.4 (C-5′′), one anomeric carbon *δ*_C_ 97.2 (C-1′′), and one monosaccharide anomeric proton *δ*_H_ 4.86 (1H, dd, *J* = 9.9, 1.6 Hz, H-1′′).^14^ One cinnamoyl group, two olefinic carbons *δ*_C_ 140.3 (C-5), 120.0 (C-7′), and one cymaropyranosyl group accounted for eight out of the twelve degrees of unsaturation, and the remaining four indicated that compound 1 possesses a tetracyclic carbon skeleton. The presence of three methyls *δ*_H_ 1.39 (3H, s, H-18), 1.15 (3H, s, H-19), 1.29 (3H, d, *J* = 6.3 Hz, H-21), *δ*_C_ 9.8 (C-18), 18.7 (C-19), 15.2 (C-21), seven methylenes, three oxygenated methines *δ*_H_ 3.51 (1H, m, H-3), 3.45 (1H, dd, *J* = 10.9, 3.9 Hz, H-12), 5.27 (1H, q, *J* = 6.3 Hz, H-20), *δ*_C_ 79.3 (C-3), 71.3 (C-12), 76.3 (C-20); three oxygenated quaternary carbons *δ*_C_ 75.1 (C-8), 89.5 (C-14), 88.9 (C-17), one olefinic proton *δ*_H_ 5.33 (1H, br. s, H-6), and two olefinic carbons *δ*_C_ 140.3 (C-5), 120.1 (C-6), suggested that compound 1 possessed a 3β,8β,12β,14β,17β,20-hexahydroxypregn-5-ene skeleton, and the aglycone was identified as sarcostin.^15^ Its ^1^H and ^13^C NMR data (Tables 1 and 2) were unambiguously assigned by analysis of the DEPT, HSQC, HMBC, ^1^H–^1^H COSY spectra. The ^13^C-NMR chemical shift *δ*_C_ 35.9 (C-2′′) of methylene group and coupling constant (9.9, 1.6 Hz) of axial anomeric proton allowed the identification of one β-cymaropyranosyl moiety.^16^ Acid hydrolysis of 1 yielded *d*-cymaropyranose, which was identified by its specific dextrorotatory value.^16^ The ^1^H–^1^H COSY spectrum suggested the presence of eight spin-coupling systems, H-1/H-2/H-3/H-4, H-6/H-7, H-9/H-11/H-12, H-15/H-16, H-20/H-21, H-2′/H-3′/H-4′/H-5′/H6′, H-7′/H8′, and H-1′′/H-2′′/H-3′′/H-4′′/H-5′′/H-6′′, as shown in Fig. 2. In the HMBC spectrum, the correlation (Fig. 2) between H-20 (*δ*_H_ 5.27) and C-9′ (*δ*_C_ 167.7) indicated that 20-OH was esterified by cinnamic acid. The HMBC cross peak of H-1′′ (*δ*_H_ 4.86) with C-3 (*δ*_C_ 79.3) suggested that the aglycone was glycosylated at C-3 by cymarose.


^1^H NMR data of compounds 1–14^a^

**Table:** 

No.	1^b^	2^b^	3^b^	4^b^	5^b^	7^c^	8^b^
1	1.86, m; 1.09, m	1.89, m; 1.16, m	1.86, m; 1.10, m	1.87, m; 1.08, m	1.84, m; 1.11, m	1.85, m; 1.08, m	1.90, m; 1.10, m
2	1.53, m; 1.84, m	1.61, m; 1.90, m	1.57, m; 1.84, m	1.59, m; 1.84, m	1.59, m; 1.84, m	1.58, m; 1.89, m	1.87, m; 1.63, m
3	3.51, m	3.56, m	3.52, m	3.58, m	3.59, m	3.53, m	3.54, m
4	2.36, m; 2.22, m	2.39, m; 2.26, m	2.34, m; 1.99, m	2.34, m; 2.20, m	2.39, dd (12.6, 3.9); 2.20, m	2.37, m; 2.27, m	2.36, m; 2.23, m
6	5.33, br s	5.38, br s	5.34, br s	5.32, br s	5.37, br s	5.37, br s	5.34, br s
7	2.13, m	2.18, m	2.17, m	2.13, m	2.14, m	2.17, m	2.12, m
9	1.44, m	1.53, m	1.55, m	1.44, m	1.55, m	1.51, m	1.52, m
11	1.60, m; 1.32, m	2.15, m; 1.83, m	1.96, m; 1.65, m	1.87, m; 1.51, m	2.07, m; 1.54, m	2.07, m; 1.55, m	2.06, m; 1.68, m
12	3.45, dd (10.9, 3.9)	4.93, dd (11.2, 4.3)	4.86, dd (11.5, 4.2)	3.47, dd (110, 4.1)	4.77, dd (11.5, 4.3)	4.87, dd (11.4, 4.3)	4.70, dd (11.5, 4.2)
15	1.89, m	2.01, m; 1.90, m	2.16, m	1.88, m; 1.82, m	1.86, m; 1.77, m	1.88, m	1.91, m; 1.72, m
16	1.85, m; 1.52, m	1.85, m	2.01, m; 1.87, m	1.85, m	1.78, m	1.80, m	1.82, m; 1.78, m
18	1.39, s	1.70, s	1.63, s	1.38, s	1.60, s	1.62, s	1.55, s
19	1.15, s	1.18, s	1.11, s	1.14, s	1.19, s	1.13, s	1.15, s
20	5.27, q (6.3)	3.61, q (6.3)	4.79, q (6.2)	5.28, q (6.2)	3.54, q (6.2)	3.55, q (6.2)	3.48, q (6.3)
21	1.29, d (6.3)	1.06, d (6.3)	1.32, d (6.4)	1.28, d (6.2)	1.25, d (6.2)	1.24, d (6.2)	1.07, d (6.3)
2′	7.59, m	8.16, d (8.4)	7.24, d (7.3)	7.59, m	7.65, m	8.07, d (7.2)	
3′	7.40, m	7.50, t (7.9)	7.32, m	7.38, m	7.43, m	7.45, t (7.7)	7.00, qd (7.1, 1.3)
4′	7.40, m	7.62, t (7.5)	7.32, m	7.38, m	7.43, m	7.57, t (7.4)	1.82, d (7.1)
5′	7.40, m	7.50, t (7.9)	7.32, m	7.38, m	7.43, m	7.45, t (7.7)	1.87, s
6′	7.59, m	8.16, d (8.4)	7.24, d (7.3)	7.59, m	7.65, m	8.07, d (7.2)	
7′	7.74, d (16.1)		7.35, d (16.0)	7.73, d (16.0)	7.81, d (16.0)		
8′	6.52, d (16.1)		6.05, d (16.0)	6.50, d (16.0)	6.66, d (16.0)		
1′′	4.86, dd (9.9, 1.6)	4.88, dd (9.6, 1.8)		4.85, dd (9.7, 1.4)	4.85, dd (9.7, 1.4)	4.82, dd (9.7, 1.6)	4.82, dd (10.0, 1.3)
2′′	2.13, m; 1.51, m	2.15, m; 1.54, m	7.94, d (7.2)	2.22, m; 1.53, m	2.23, m; 1.56, m	2.17, m; 1.59, m	2.12, m; 1.49, m
3′′	3.59, m	3.62, m	7.32, m	3.49, m	3.52, m	3.58, m	3.59, m
4′′	3.16, dd (9.5, 3.0)	3.19, dd (9.4, 3.0)	7.54, t (7.5)	3.22, dd (9.7, 2.8)	3.24, dd (9.6, 2.9)	3.19, dd (9.6, 2.8)	3.16, dd (9.5, 3.1)
5′′	3.71, m	3.74, m	7.32, m	3.79, m	3.81, m	3.83, m	3.71, m
6′′	1.21, d (6.3)	1.25, d (6.2)	7.94, d (7.2)	1.18, d (6.3)	1.22, d (6.2)	1.03, d (6.2)	1.22, d (6.3)
7′′	3.43, s	3.46, s		3.42, s	3.42, s	3.40, s	3.43, s
1′′′			4.84, dd (9.8, 2.0)	4.77, dd (9.6, 1.7)	4.79, dd (9.8, 1.6)	4.65, dd (9.7, 1.6)	
2′′′			2.14, m; 1.50, m	2.06, m; 1.52, m	2.07, m; 1.53, m	2.07, m; 1.53, m	
3′′′			3.58, m	3.84, m	3.84, m	3.77, m	
4′′′			3.15, dd (9.6, 3.2)	3.16, dd (9.6, 3.2)	3.19, dd (9.6, 3.2)	3.17, dd (9.6, 2.8)	
5′′′			3.71, m	3.72, m	3.72, m	3.53, m	
6′′′			1.20, d (6.2)	1.22, d (6.3)	1.11, d (6.3)	1.20, d (6.2)	
7′′′			3.43, s	3.42, s	3.42, s	3.42, s	

a Recorded at 500 MHz. *δ*_H_ in ppm, *J* in Hz.

b Recorded in methanol-*d*_4_.

c Recorded in chloroform-*d*_1_.

**Table:** 

No.	6^b^	9^b^	10^b^	11^b^	12^b^	13^b^	14^b^
1	1.76, m; 1.09, m	1.84, m; 1.10, m	1.85, m; 1.13, m	1.90, m; 1.10, m	1.90, m; 1.10, m	1.79, m; 1.10, m	1.79, m; 1.10, m
2	1.54, m; 1.82, m	1.60, m; 1.87, m	1.63, m; 1.89, m	1.87, m; 1.61, m	1.60, m; 1.87, m	1.59, m; 1.85, m	1.59, m; 1.86, m
3	3.58, m	3.53, m	3.54, m	3.42, m	3.52, m	3.50, m	3.51, m
4	2.34, m; 2.19, m	2.37, m; 2.23, m	2.39, m; 2.26, m	2.36, m; 2.21, m	2.38, m; 2.21, m	2.35, m; 2.21, m	2.33, m; 2.21, m
6	5.35, br s	5.34, br s	5.36, br s	5.33, br s	5.34, br s	5.33, br s	5.33, br s
7	2.19, m	2.13, m	2.17, m	2.13, m	2.14, m	2.12, m	2.13, m
9	1.58, m	1.54, m	1.53, m	1.49, m	1.49, m	1.49, m	1.49, m
11	1.58, m; 2.04, m	1.64, m; 2.03, m	1.66, m; 2.04, m	1.95, m; 1.61, m	1.62, m; 1.93, m	1.62, m; 1.93, m	1.61, m; 1.92, m
12	5.03, dd (11.4, 4.3)	4.71, dd (11.6, 4.3)	4.73, dd (11.5, 4.2)	4.67, dd (11.5, 4.1)	4.68, dd (11.5, 4.1)	4.65, dd (11.5, 4.2)	4.66, dd (11.4, 4.1)
15	1.95, m; 2.06, m	1.92, m; 1.85, m	1.95, m	1.89, m	1.89, m	1.95, m; 1.88, m	1.95, m; 1.87, m
16	1.56, m	1.77, m; 1.82, m	1.79, m	1.95, m; 1.89, m	1.94, m; 1.89, m	1.88, m	1.87, m
18	1.68, s	1.55, s	1.57, s	1.45, s	1.46, s	1.45, s	1.45, s
19	1.10, s	1.15, s	1.17, s	1.13, s	1.13, s	1.12, s	1.12, s
20	4.82, q (6.1)	3.48, q (6.3)	3.52, q (6.2)	4.56, q (6.2)	4.58, q (6.2)	4.56, q (6.0)	4.56, q (6.5)
21	1.27, d (6.1)	1.07, d (6.3)	1.21, d (6.2)	1.20, d (6.2)	1.21, d (6.2)	1.20, d (6.0)	1.20, d (6.5)
2′	7.62, d (8.1)			1.88, s	1.89, s	1.88, s	1.88, s
3′	7.09, t (7.8)	7.01, q (7.1)	7.02, qd (7.1, 1.3)				
4′	7.43, t (7.4)	1.82, d (7.1)	1.85, d (7.1)				
5′	7.09, t (7.8)	1.88, s	1.90, s				
6′	7.62, d (8.1)						
1′′		4.88, dd (9.6, 1.9)	4.84, dd (9.6, 1.6)				
2′′	7.62, d (8.1)	2.09, m; 1.54, m	2.07, m; 1.57, m				
3′′	7.33, t (7.8)	3.84, m	3.84, m	7.01, q (6.2)	7.01, q (6.2)	7.00, qd (7.0, 1.0)	7.00, qd (7.0, 1.0)
4′′	7.54, t (7.4)	3.29, dd (9.1, 4.1)	3.27, dd (9.3, 3.0)	1.82, d (7.1)	1.84, d (7.0)	1.83, d (7.1)	1.82, d (7.1)
5′′	7.33, t (7.8)	3.84, m	3.82, m	1.86, s	1.86, s	1.85, s	1.85, s
6′′	7.62, d (8.1)	1.21, d (6.3)	1.21, d (6.1)				
7′′		3.43, s	3.45, s				
1′′′	4.85, dd (9.7, 1.6)	4.61, dd (9.0, 1.8)	4.60, dd (9.7, 1.5)	4.86, dd (9.6, 1.7)	4.87, dd (9.6, 1.7)	4.86, dd (9.6, 1.8)	4.86, dd (9.6, 1.7)
2′′′	2.04, m; 1.55, m	2.34, m; 1.37, m	2.10, m; 1.57, m	2.13, m; 1.52, m	2.07, m; 1.55, m	2.03, m; 1.55, m	2.05, m; 1.58, m
3′′′	3.50, m	3.21, m	3.84, m	3.59, m	3.85, m	3.60, m	3.81, m
4′′′	3.21, dd (9.6, 2.9)	2.97, t (9.0)	3.26, dd (9.4, 3.0)	3.16, dd (9.6, 3.1)	3.26, dd (9.1, 2.5)	3.22, dd (9.6, 2.9)	3.30, dd (9.6, 3.3)
5′′′	3.80, m	3.27, m	3.82, m	3.72, m	3.82, m	3.81, m	3.81, m
6′′′	1.22, d (6.2)	1.28, d (6.2)	1.08, d (6.2)	1.22, d (6.3)	1.21, d (6.2)	1.18, d (6.4)	1.21, d (6.1)
7′′′	3.42, s	3.44, s	3.44, s	3.43, s	3.44, s	3.42, s	3.42, s
1′′′′	4.79, dd (9.6, 3.2)		4.73, dd (9.6, 1.6)		4.60, dd (9.7, 1.5)	4.76, dd (9.8, 1.8)	4.78, dd (9.7, 1.8)
2′′′′	2.05, m; 1.53, m		2.34, m; 1.36, m		2.33, m; 1.37, m	2.21, m; 1.56, m	2.02, m; 1.58, m
3′′′′	3.83, m		3.20, m		3.42, m	3.84, m	3.81, m
4′′′′	3.15, dd (9.6, 3.2)		2.97, t (9.0)		2.97, t (9.0)	3.16, dd (9.7, 3.2)	3.24, dd (9.7, 3.0)
5′′′′	3.70, m		3.26, m		3.26, m	3.72, m	3.80, m
6′′′′	1.17, d (6.2)		1.30, d (6.2)		1.28, d (6.2)	1.22, d (6.5)	1.18, d (6.3)
7′′′′	3.42, s		3.45, s		3.42, s	3.41, s	3.42, s
1′′′′′							4.59, dd (9.8, 1.8)
2′′′′′							2.32, m; 1.37, m
3′′′′′							3.20, m
4′′′′′							2.97, t (9.0)
5′′′′′							3.27, m
6′′′′′							1.27, d (6.2)
7′′′′′							3.41, s

**Table:** ^13^C NMR data of compounds 1–14^a^

No.	1^b^	2^b^	3^b^	4^b^	5^b^	6^b^	7^c^	8^b^	9^b^	10^b^	11^b^	12^b^	13^b^	14^b^
1	39.8 t	39.8 t	39.7 t	39.8 t	39.8 t	39.7 t	38.9 t	39.8 t	39.8 t	39.8 t	39.7 t	39.7 t	39.7 t	39.7 t
2	30.2 t	30.2 t	30.1 t	30.2 t	30.2 t	30.1 t	29.0 t	30.2 t	30.2 t	30.2 t	30.1 t	30.1 t	30.1 t	30.1 t
3	79.3 d	79.3 d	79.22 d	79.4 d	79.3 d	79.3 d	78.0 d	79.3 d	79.3 d	79.3 d	79.3 d	79.3 d	79.2 d	79.2 d
4	39.9 t	39.8 t	39.8 t	39.9 t	39.8 t	39.8 t	38.8 t	39.8 t	39.8 t	39.8 t	39.8 t	39.8 t	39.7 t	39.8 t
5	140.3 s	140.1 s	140.1 s	140.3 s	140.1 s	140.1 s	139.7 s	140.0 s	140.1 s	140.1 s	140.0 s	140.1 s	140.0 s	140.1 s
6	120.1 d	120.0 d	120.1 d	120.0 d	120.0 d	119.8 d	118.4 d	120.0 d	120.0 d	120.0 d	119.8 d	119.8 d	120.0 d	119.8 d
7	35.3 t	35.3 t	35.2 t	35.3 t	35.2 t	35.2 t	33.7 t	35.2 t	35.2 t	35.2 t	35.1 t	35.1 t	35.1 t	35.1 t
8	75.1 s	75.0 s	75.0 s	75.1 s	75.5 s	75.0 s	72.4 s	75.0 s	75.2 s	75.2 s	75.3 s	75.4 s	74.9 s	75.5 s
9	45.3 d	44.8 d	44.7 d	45.4 d	44.8 d	44.6 d	43.4 d	44.7 d	44.7 d	44.7 d	44.7 d	44.7 d	44.6 d	44.7 d
10	38.0 s	38.1 s	38.0 s	38.0 s	38.0 s	38.0 s	37.0 s	38.0 s	38.0 s	38.0 s	38.0 s	38.0 s	37.9 s	38.0 s
11	29.8 t	26.0 t	26.1 t	29.8 t	26.0 t	26.3 t	24.7 t	26.0 t	26.0 t	26.0 t	26.0 t	26.0 t	26.0 t	26.0 t
12	71.3 d	75.5 d	75.2 d	71.3 d	75.0 d	75.5 d	74.0 d	75.1 d	75.0 d	75.0 d	75.0 d	75.0 d	75.4 d	75.0 d
13	59.2 s	57.7 s	57.7 s	58.1 s	57.5 s	57.8 s	56.3 s	57.6 s	57.7 s	57.4 s	57.4 s	54.8 s	57.3 s	57.4 s
14	89.5 s	89.2 s	88.5 s	88.9 s	89.1 s	88.6 s	87.9 s	89.2 s	89.2 s	89.2 s	88.5 s	88.5 s	88.4 s	88.5 s
15	34.2 t	34.4 t	34.5 t	34.2 t	34.3 t	34.5 t	33.3 t	34.3 t	34.3 t	34.3 t	34.2 t	34.2 t	34.1 t	34.2 t
16	34.1 t	33.5 t	34.1 t	34.1 t	33.6 t	34.1 t	31.7 t	33.5 t	33.5 t	33.5 t	33.0 t	34.0 t	34.0 t	34.0 t
17	88.9 s	89.4 s	89.6 s	89.5 s	89.3 s	89.6 s	87.9 s	89.3 s	89.3 s	89.3 s	89.5 s	89.5 s	89.4 s	89.4 s
18	9.8 q	11.3 q	11.3 q	9.8 q	11.2 q	11.3 q	11.1 q	11.2 q	11.2 q	11.2 q	10.9 q	10.9 q	10.9 q	10.9 q
19	18.7 q	18.5 q	18.5 q	18.6 q	18.5 q	18.5 q	18.2 q	18.5 q	18.9 q	18.4 q	18.7 q	18.5 q	18.5 q	18.6 q
20	76.3 d	71.6 d	76.4 d	76.3 d	71.7 d	76.0 d	74.6 d	71.4 d	71.6 d	71.6 d	75.5 d	75.5 d	75.3 d	75.3 d
21	15.2 q	18.9 q	15.2 q	15.2 q	18.7 q	15.2 q	15.5 q	18.9 q	18.5 q	15.1 q	15.2 q	15.2 q	15.2 q	15.2 q
1′	136.1 s	131.9 s	135.6 s	136.1 s	135.9 s	132.2 s	130.1 s	169.1 s	169.2 s	169.2 s	173.0 s	173.0 s	172.9 s	172.9 s
2′	129.1 d	131.0 d	129.3 d	129.1 d	129.4 d	130.5 d	129.6 d	139.6 s	139.4 s	139.6 s	22.1 q	22.1 q	22.1 q	22.1 q
3′	130.0 d	129.5 d	129.8 d	130.0 d	130.0 d	129.2 d	128.7 d	130.1 d	130.0 d	130.1 d				
4′	131.3 d	134.3 d	131.3 d	131.3 d	131.5 d	133.7 d	133.4 d	14.5 q	14.5 q	14.5 q				
5′	130.0 d	129.5 d	129.8 d	130.0 d	130.0 d	129.2 d	128.7 d	12.1 q	12.1 q	12.1 q				
6′	129.1 d	131.0 d	129.3 d	129.1 d	129.4 d	130.5 d	129.6 d							
7′	120.0 d	167.8 s	119.8 d	119.9 d	119.3 d	167.8 s	165.8 s							
8′	145.7 d		145.3 d	145.7 d	146.8 d									
9′	167.7 s		168.0 s	167.7 s	168.4 s									
1′′	97.2 d	97.2 d	131.7 s	97.2 d	97.2 d	131.6 s	96.1 d	97.2 d	97.2 d	97.2 d	168.5 s	168.6 s	168.5 s	168.5 s
2′′	35.9 t	35.9 t	131.0 d	35.6 t	35.6 t	130.8 d	34.5 t	35.9 t	36.7 t	36.6 t	139.5 s	139.5 s	139.4 s	139.5 s
3′′	79.2 d	79.2 d	129.5 d	79.2 d	79.2 d	129.2 d	77.4 d	79.2 d	78.5 d	78.5 d	130.0 d	130.0 d	129.9 d	130.0 d
4′′	74.5 d	74.5 d	134.2 d	83.8 d	83.8 d	134.0 d	82.5 d	74.5 d	83.9 d	83.9 d	14.5 q	14.5 q	14.5 q	14.5 q
5′′	71.4 d	71.4 d	129.5 d	70.0 d	70.0 d	129.2 d	68.5 d	71.5 d	70.0 d	69.8 d	12.2 q	12.2 q	12.2 q	12.2 q
6′′	18.6 q	18.7 q	131.0 d	18.5 q	18.5 q	130.8 d	18.2 q	18.7 q	18.5 q	18.5 q				
7′′	58.1 q	58.1 q	166.9 s	58.4 q	58.1 q	166.6 s	57.2 q	58.1 q	57.4 q	57.6 q				
1′′′			97.2 d	101.2 d	101.2 d	97.2 d	99.4 d		102.8 d	101.2 d	97.2 d	97.2 d	97.2 d	97.2 d
2′′′			35.9 t	36.7 t	36.6 t	35.6 t	35.5 t		37.4 t	36.4 t	35.9 t	36.7 t	36.6 t	36.6 t
3′′′			79.17 d	78.6 d	78.6 d	79.2 d	77.3 d		81.6 d	78.6 d	79.2 d	78.5 d	79.1 d	78.5 d
4′′′			74.4 d	74.5 d	74.4 d	83.8 d	71.0 d		77.0 d	83.9 d	74.7 d	83.8 d	83.8 d	83.8 d
5′′′			71.4 d	71.3 d	71.3 d	70.0 d	70.7 d		73.3 d	70.0 d	71.4 d	69.9 d	69.9 d	69.8 d
6′′′			18.7 q	18.7 q	18.8 q	18.5 q	18.3 q		18.4 q	18.9 q	18.4 q	18.4 q	18.5 q	18.5 q
7′′′			58.1 q	59.3 q	58.5 q	58.1 q	58.0 q		58.5 q	58.5 q	58.1 q	58.5 q	58.1 q	57.4 q
1′′′′						101.2 d				102.8 d		102.8 d	101.1 d	101.2 d
2′′′′						36.6 t				37.4 t		37.4 t	35.6 t	36.4 t
3′′′′						78.6 d				81.6 d		81.6 d	78.5 d	78.54 d
4′′′′						74.4 d				77.0 d		77.0 d	74.4 d	83.8 d
5′′′′						71.3 d				73.3 d		73.3 d	71.2 d	69.9 d
6′′′′						18.7 q				18.6 q		18.4 q	18.8 q	18.5 q
7′′′′						58.4 q				58.4 q		57.4 q	58.4 q	58.5 q
1′′′′′														102.8 d
2′′′′′														37.4 t
3′′′′′														81.6 d
4′′′′′														76.9 d
5′′′′′														73.2 d
6′′′′′														18.4 q
7′′′′′														58.4 q

a Recorded at 125 MHz.

b Recorded in methanol-*d*_4_.

c Recorded in chloroform-*d*_1_.

**Fig. 3:**
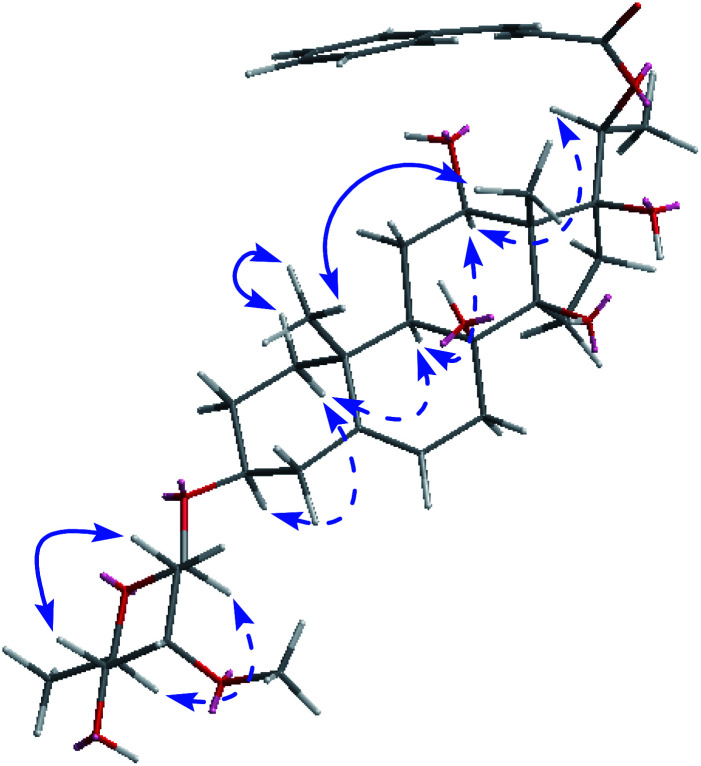
Selected NOE correlations of compound 1.

The absolute configuration of the ring substituents of aglycone skeleton was determined by analysis of the ECD and NOESY spectrum (Fig. 3). The ECD curve of C21 steroid with a (2*E*,4*E*)-5-phenyl-2,4-pentadienoate group at C-20 (lyciumsterol A) showed that 20*S* derivative gave positive Cotton effect at around 300 nm, while 20*R* derivative showed the negative Cotton effect.^16^ The ECD spectrum of compound 1 exhibited the positive Cotton effect at 279 nm. Consequently, the absolute configuration of C-20 was determined to be *S*.^16^ The NOESY correlations (Fig. 3) from H-1α to H-3 and H-9, from H-9 to H-12, from H-16α to H-20, from H-12 to H-20, from H-19 to H-18, and from H-1β to H-19, indicated the α-orientation for H-3, H-9, H-12, and H-20, and the β-orientation for Me-19 and Me-18. All pregnanes from natural sources possess the *trans*/*trans*/*cis* connection modes for the A, B, C, and D rings, so 8-OH and 14-OH were β-oriented.^16^ In combination with the same biosynthetic relationship, the absolute configurations of the chiral carbons in the pregnane skeleton were defined as 3*S*, 8*S*, 9*S*, 10*R*, 12*S*, 13*S*, 14*R*, and 17*S* in 1.^15–18^ Based on these data, compound 1 was established as 20-*O-trans*-cinnamoylsarcostin 3-*O*-β-d-cymaropyranoside, and named cissasteroid A.

Compound 2 was obtained as a white amorphous powder. Its ^1^H and ^13^C NMR spectra (Tables 1 and 2) were analogous to those of 1, except that benzoyl group [one monosubstituted benzene ring *δ*_H_ 8.16 (2H, d, *J* = 8.4 Hz, H-2′, 6′), 7.50 (2H, t, *J* = 7.9 Hz, H-3′, 5′), 7.62 (1H, t, *J* = 7.5 Hz, H-4′), on ester carbonyl *δ*_C_ 167.8 (C-7′)] was observed in 2 instead of the cinnamoyl group in 1. This was further supported by their HR-ESI-MS, which gave a sodium adduct ion *m*/*z* 653.3304 (calcd 653.3302) in 2, with 26 mass-units less than that of 1. The HMBC correlation (Fig. 2) between H-12 (*δ*_H_ 4.93) and C-7′ (*δ*_C_ 167.8), indicated that 12-OH was esterified by benzoic acid. Hence, compound 2 was assigned as 12-*O*-benzoylsarcostin 3-*O*-β-d-cymaropyranoide, and named cissasteroid B.

Compound 3 was obtained as a white amorphous powder. Its ^1^H and ^13^C NMR data (Tables 1 and 2) were almost superimposable on those of 2, except that one additional cinnamoyl group [one monosubstituted benzene ring *δ*_H_ 7.24 (2H, d, *J* = 7.3 Hz, H-2′, 6′), 7.32 (3H, m, H-3′, 4′, 5′), one set of *trans* conjugated olefinic protons *δ*_H_ 7.35 (1H, d, *J* = 16.0 Hz, H-7′), 6.05 (1H, d, *J* = 16.0 Hz, H-8′), one ester carbonyl *δ*_C_ 168.0 (C-9′)] was observed in 3. This was further supported by their HR-ESI-MS, which gave a sodium adduct ion *m*/*z* 783.3720 (calcd 783.3720) in 3, with 130 mass-units more than that of 2. The HMBC correlations (Fig. 2) from H-12 (*δ*_H_ 4.86) to C-9′ (*δ*_C_ 168.0), from H-20 (*δ*_H_ 4.79) to C-7′′ (*δ*_C_ 166.9), indicated that 12-OH and 20-OH were esterified by cinnamic acid and benzoic acid, respectively. Thus, compound 3 was identified as 12-*O-trans*-cinnamoyl-20-*O*-benzoylsarcostin 3-*O*-β-d-cymaropyranoside, and named cissasteroid C.

Compound 4 was obtained as a white amorphous powder. Its ^1^H and ^13^C NMR spectra (Tables 1 and 2) bore a resemblance to those of 1, with the notable difference given by the presence of one additional β-cymaropyranosyl group [the anomeric proton *δ*_H_ 4.77 (1H, dd, *J* = 9.6, 1.7 Hz, H-1′′′), seven carbon signals *δ*_C_ 101.2 (C-1′′′), 36.7 (C-2′′′), 78.6 (C-3′′′), 74.5 (C-4′′′), 71.3 (C-5′′′), 18.7 (C-6′′′), 59.3 (C-7′′′)] in 4. Acid hydrolysis of 4 yielded only d-cymaropyranose. The HMBC cross peaks of H-1′′ (*δ*_H_ 4.85) with C-3 (*δ*_C_ 79.4), and H-1′′′ (*δ*_H_ 4.77) with C-4′′ (*δ*_C_ 83.8), indicated that one cymaropyranosyl was at C-3 of the aglycone and the other was substituted at C-4′′ of the inner cymarose. Therefore, compound 4 was identified as 20-*O-trans*-cinnamoylsarcostin 3-*O*-β-d-cymaropyransyl-(1→4)-β-d-cymaropyranoside, and named cissasteroid D.

Compound 5 was obtained as a white amorphous powder. It gave the same molecular formula C_44_H_64_O_13_ as that of 4, based on a sodium adduct ion *m*/*z* 823.4244 (calcd 823.4245). A comparison of the NMR spectroscopic data demonstrated that the difference between these two compounds was in the linkage position of the cinnamoyl group. The HMBC correlation from H-12 (*δ*_H_ 4.77) to C-9′ (*δ*_C_ 168.4) confirmed that the cinnamoyl group was located at C-12. From the above analysis, compound 5 was characterized as 12-*O-trans*-cinnamoylsarcostin 3-*O*-β-d-cymaropyransyl-(1→4)-β-d-cymaropyranoside, and named cissasteroid E.

Compound 6 was obtained as a white amorphous powder. Its ^1^H and ^13^C NMR spectra (Tables 1 and 2) were almost consistent with those of 5, except that two benzoyl groups [two monosubstituted benzene rings *δ*_H_ 7.62 (4H, t, *J* = 8.1 Hz, H-2′, 6′, 2′′, 6′′), 7.09 (2H, t, *J* = 7.8 Hz, H-3′, 5′), 7.43 (1H, t, *J* = 7.4 Hz, H-4′), 7.33 (2H, t, *J* = 7.8 Hz, H-3′′, 5′′), 7.54 (1H, t, *J* = 7.4 Hz, H-4′′), two ester carbonyls *δ*_C_ 167.8 (C-7′), 166.6 (C-7′′)] were observed in 6 instead of one cinnamoyl group in 5. This was further confirmed by their HR-ESI-MS, which gave a sodium adduct ion *m*/*z* 901.4350 (calcd 901.4350) in 6, with 78 mass-units more than that of 5. Moreover, the HMBC correlations of H-12 (*δ*_H_ 5.03) with C-7′ (*δ*_C_ 167.8), and of H-20 (*δ*_H_ 4.82) with C-7′′ (*δ*_C_ 166.6), suggested that 12-OH and 20-OH were esterified by benzoic acids. Consequently, compound 6 was designated as 12,20-*O*-dibenzoylsarcostin 3-*O*-β-d-cymaropyranoide-(1→4)-β-d-cymaropyranoside, and named cissasteroid F.

Compound 7 was obtained as a white amorphous powder. Its ^1^H and ^13^C NMR spectra (†) were analogous to those of 6, except that the absence of one benzoyl group in 7. This was further supported by their HR-ESI-MS, which gave a sodium adduct ion *m*/*z* 797.4087 (calcd 797.4088) in 7, being 104 mass-units less than that of 6. The HMBC correlation (Fig. 2) between H-12 (*δ*_H_ 4.87) and C-7′ (*δ*_C_ 165.8), indicated that 12-OH was esterified by benzoic acid. Thus, compound 7 was designated as 12-*O*-benzoylsarcostin 3-*O*-β-d-cymaropyranoide-(1→4)-β-d-cymaropyranoside, and named cissasteroid G.

Compound 8 was obtained as a white amorphous powder. Its ^1^H and ^13^C NMR spectra (Tables 1 and 2) were closely related to those of 1, except that one tigloyl group [one olefinic proton *δ*_H_ 7.00 (1H, qd, *J* = 7.1, 1.3 Hz, H-3′), one tertiary methyl *δ*_H_ 1.87 (3H, s, H-5′), *δ*_C_ 12.1 (C-5′), one secondary methyl *δ*_H_ 1.82 (3H, d, *J* = 7.1 Hz, H-4′), *δ*_C_ 14.5 (C-4′), two olefinic carbons *δ*_C_ 139.6 (C-2′), 130.1 (C-3′), and one ester carbonyl *δ*_C_ 169.1 (C-1′)] was observed in 8 instead of the cinnamoyl group in 1. This was further supported by their HR-ESI-MS, which gave a sodium adduct ion *m*/*z* 631.3458 (calcd 631.3458) in 8, with 48 mass-units less than that of 1. The HMBC correlation (Fig. 2) between H-12 (*δ*_H_ 4.70) and C-1′ (*δ*_C_ 169.1), indicated that 12-OH was esterified by tiglic acid. Thus, compound 8 was established as 12-*O*-tigloylsarcostin 3-*O*-β-d-cymaropyranoside, and named cissasteroid H.

Compound 9 was obtained as a white amorphous powder. Its ^1^H and ^13^C NMR spectra (Tables 1 and 2) were quite similar to those of 8, except that one oleandropyranosyl group [*δ*_C_ 102.8 (C-1′′′), 37.4 (C-2′′′), 81.6 (C-3′′′), 77.0 (C-4′′′), 73.3 (C-5′′′), 18.4 (C-6′′′), 58.5 (C-7′′′) ]was observed in 9. This was further supported by their HR-ESI-MS, which gave a sodium adduct ion *m*/*z* 775.4244 (calcd 775.4245) in 9, with 144 mass-units more than that of 8. d-Oleandropyranose was identified by acid hydrolysis and specific rotation value.^16^ The β-configuration of d-oleandropyranose was determined by the large coupling constants (*J* = 9.0, 1.8 Hz) of the anomeric proton and the chemical shifts (*δ*_C_ 37.4, C-2′′′) of the methylene carbon.^16^ The HMBC cross peaks of H-1′′ (*δ*_H_ 4.88) with C-3 (*δ*_C_ 79.3), and H-1′′′ (*δ*_H_ 4.61) with C-4′′ (*δ*_C_ 83.9), indicated that the cymaropyranosyl group was at C-3 of the aglycone and the oleandrose was substituted at C-4′′ of the inner cymarose. Thus, compound 9 was identified as 12-*O*-tigloylsarcostin 3-*O*-β-d-oleandropyranosyl-(1→4)-β-d-cymaropyranoside, and named cissasteroid I.

Compound 10 was isolated as a white amorphous powder. Its ^1^H and ^13^C NMR (Tables 1 and 2) bore a resemblance to those of 9, with the obvious difference being the resonances of seven carbon signals [*δ*_C_ 101.2 (C-1′′′), 36.4 (C-2′′′), 78.6 (C-3′′′), 83.9 (C-4′′′), 70.0 (C-5′′′), 18.9 (C-6′′′), and 58.5 (C-7′′′)] and one anomeric proton *δ*_H_ 4.60 (1H, dd, *J* = 9.7, 1.5 Hz, H-1′′′), which indicated the occurrence of one additional β-cymaropyranosyl moiety. Furthermore, the absolute configurations of the three deoxysugars were confirmed as D-series by the same method as 9. The sequence of this trisaccharide moiety was established as β-d-oleandropyranosyl-(1→4)-β-d-cymaropyranosyl-(1→4)-β-d-cymaropyranoside, based on the HMBC correlations from H-1′′′′ (*δ*_H_ 4.73) to C-4′′′ (*δ*_C_ 83.9), and from H-1′′′ (*δ*_H_ 4.60) to C-4′′ (*δ*_C_ 83.9). In addition, the HMBC correlation from H-1′′ (*δ*_H_ 4.84) to C-3 (*δ*_C_ 79.3) suggested that the trisaccharide moiety is attached at C-3. Thus, compound 10 was defined as 12-*O*-tigloylsarcostin 3-*O*-β-d-oleandropyranosyl-(1→4)-β-d-cymaropyranosyl-(1→4)-β-d-cymaropyranoside, and named cissasteroid J.

Compound 11 was obtained as a white amorphous powder. Its ^1^H and ^13^C NMR (Tables 1 and 2) data showed a distinct similarity with those of 8, except that an acetyl group *δ*_H_ 1.88 (3H, s, H-2′), *δ*_C_ 22.1 (C-2′), 173.0 (C-1′) was observed in 11. This was further supported by their HR-ESI-MS, which gave a sodium adduct ion *m*/*z* 673.3565 (calcd 673.3564) in 11, with 42 mass-units more than that of 8. The HMBC correlations (Fig. 2) between H-12 (*δ*_H_ 4.67) and C-1′ (*δ*_C_ 173.0), between H-20 (*δ*_H_ 4.56) and C-1′′ (*δ*_C_ 168.5), indicated that 12-OH and 20-OH were esterified by acetic acid and tiglic acid, respectively. Thus, compound 11 was deduced as 12-*O*-acetyl-20-*O*-tigloylsarcostin 3-*O*-β-d-cymaropyranoide, and named cissasteroid K.

Compounds 12 and 13 were obtained as white amorphous powders. Their HR-ESI-MS showed the same molecular formula of C_42_H_66_O_14_, according to a sodium adduct ion [*m*/*z* 817.4350 in 12; *m*/*z* 817.4360 in 13 (calcd 817.4350)]. Analysis of the UV, IR, and NMR data suggested that compounds 5 and 6 possess the same planar structure. Their ^1^H and ^13^C NMR spectra (Tables 1 and 2) bore a resemblance to those of 11, except that another 2,6-deoxysugar moiety [one anomeric proton and seven carbon signals, *δ*_H_ 4.60 (1H, dd, *J* = 9.7, 1.5 Hz, H-1′′′′), *δ*_C_ 102.8 (C-1′′′′), 37.4 (C-2′′′′), 81.6 (C-3′′′′), 77.0 (C-4′′′′), 73.3 (C-5′′′′), 18.4 (C-6′′′′), 57.4 (C-7′′′′) in 12; *δ*_H_ 4.76 (1H, dd, *J* = 9.8, 1.8 Hz, H-1′′′′), *δ*_C_ 101.1 (C-1′′′′), 35.6 (C-2′′′′), 78.5 (C-3′′′′), 74.4 (C-4′′′′), 71.2 (C-5′′′′), 18.8 (C-6′′′′), 58.4 (C-7′′′′) in 13] were observed. Acid hydrolysis of 12 and 13 yielded d-oleandropyranose and d-cymaropyranose, and d-cymaropyranose, respectively. Their β-configurations were established by the large coupling constants (cymarose, *J* = 9.6, 1.7 Hz, oleandrose, *J* = 9.7, 1.5 Hz in 12; cymarose, *J* = 9.6, 1.8 Hz, cymarose, *J* = 9.8, 1.8 Hz in 13) of the anomeric protons and the chemical shifts (*δ*_C_ 36.7 (C-2′′′), 37.4 (C-2′′′′) in 12; *δ*_C_ 36.6 (C-2′′′), 35.6 (C-2′′′′) in 13) of the methylene carbons.^16^ The disaccharide moieties at C-3 in 12 and 13 were determined as β-d-oleandropyranosyl-(1→4)-β-d-cymaropyranosyl and β-d-cymaropyranosyl-(1→4)-β-d-cymaropyranosyl sugar sequences, respectively, based on the HMBC correlations from H-1′′′′ (*δ*_H_ 4.60) to C-4′′′ (*δ*_C_ 83.8) in 12, from H-1′′′′ (*δ*_H_ 4.76) to C-4′′′ (*δ*_C_ 83.8) in 13, respectively. Thus, compounds 12 and 13 were identified as 12-*O*-acetyl-20-*O*-tigloylsarcostin 3-*O*-β-d-oleandropyranosyl-(1→4)-β-d-cymaropyranoside and 12-*O*-acetyl-20-*O*-tigloylsarcostin 3-*O*-β-d-cymaropyranosyl-(1→4)-β-d-cymaropyranoside, and named cissasteroid L (12) and M (13), respectively.

Compound 14 was obtained as a white amorphous powder. Comparison of its NMR spectra with those of 13 revealed these two compounds differ by the presence of an additional oleandropyranosyl group in 14. The β-oleandropyranose was confirmed by the large coupling constant (*J* = 9.8, 1.8 Hz) of the anomeric proton and the chemical shifts *δ*_C_ 37.4 (C-2′′′′′) of the methylene carbon. Acid hydrolysis gave d-oleandrose and d-cymarose. The sugar sequence of β-d-oleandropyranosyl-(1→4)-β-d-cymaropyranosyl-(1→4)-β-d-cymaropyranoside and its linkage at C-3 were determined, based on the HMBC correlations from H-1′′′′′ (*δ*_H_ 4.59) to C-4′′′′ (*δ*_C_ 83.8), from H-1′′′′ (*δ*_H_ 4.78) to C-4′′′ (*δ*_C_ 83.8) from H-1′′′ (*δ*_H_ 4.86) to C-3 (*δ*_C_ 79.2), respectively. Consequently, compound 14 was characterized as 12-*O*-acetyl-20-*O*-tigloylsarcostin 3-*O*-β-d -oleandropyranosyl-(1→4)-β-d-cymaropyranosyl-(1→4)-β-d-cymaropyranoside, and named cissasteroid G (14).

Five known compounds were obtained and identified as isokidjoladinin (15),^17^ deacetylkidjoladinin (16),^18^ 12,20-*O*-dibenzoylsarcostin (17),^19^ 12-*O*-cinnamoyl-3β,5α,8β,12β,14β,17β,20-heptahydroxy-(20*S*)-pregn-6-ene (18),^20^ 12,20-*O*-dibenzoylsarcostin-3-*O*-β-d-cymaropyranoide (19),^21^ by comparison of their spectroscopic data with values reported in the literature.

Polyoxypregnane glycosides have been reported to show various cytotoxic or anti-proliferative activities against MCF-7, H1299, HeLa, HepG2,^22^ PC-3, HT-29,^23^ and A-549 (ref. 24) cell lines. All the isolates (1–19) were evaluated for their cytotoxicity against five human cancer cell lines: HL-60, A549, SMMC-7721, MCF-7, and SW480 (Table 3), and against NO release in LPS-induced RAW 264.7 cells. Unfortunately, they were devoid of any NO production inhibitory activity. Compared with cisplatin, compound 7 showed more potent cytotoxicities against the HL-60, A549, SMMC-7721, MCF-7, and SW480 cell lines, with IC_50_ values of 2.19, 14.38, 2.00, 7.58, and 7.44 μM, respectively. However, all the tested compounds were less active than paclitaxel. Compound 7 was more cytotoxic than compound 6, suggesting that benzoic acid esterification at C-20 may have a negative effect on the cytotoxic activity of polyhydroxylated pregnane derivatives against these five cell lines.

**Table:** Cytotoxicities of compounds 1–19 against HL-60, A549, SMMC-7721, MCF-7 and SW480 cell lines (IC_50_, μM)

No.	HL-60	A549	SMMC-7721	MCF-7	SW480	No.	HL-60	A549	SMMC-7721	MCF-7	SW480
3	14.85 ± 0.55	>40	24.05 ± 0.61	30.97 ± 0.48	>40	1, 2, 4, 5, 8–16, 18, 19	>40	>40	>40	>40	>40
6	36.74 ± 0.72	>40	>40	>40	>40	Cisplatin	3.38 ± 0.23	24.58 ± 1.30	18.25 ± 0.57	20.37 ± 0.71	11.79 ± 1.08
7	2.19 ± 0.07	14.38 ± 0.65	2.00 ± 0.08	7.58 ± 0.25	7.44 ± 0.22	Paclitaxel	<0.008	<0.008	1.68 ± 0.21	<0.008	<0.008
17	>40	>40	32.81 ± 1.47	>40	>40						

## Conclusions

Compounds 1–19 represent the first report of polyhydroxylated pregnane glycosides from the genus *Cissampelos*. This also lays a solid chemical foundation for pharmacological research of *C. pareira* var. *hirsuta*. Compound 7 was the most promising of all isolated compounds based upon their IC_50_ values. Further studies are necessary to explore antitumor mechanism, cytotoxicities in normal cells, and structure optimization.

## Experimental method

### General experimental procedures

Optical rotations and ECD spectra were measured by a Rudolph AP-IV polarimeter (Rudolph, Hackettstown, NJ, USA) and an Applied Photophysics Chirascanq CD spectropolarimeter (Applied Photophysics, Leatherhead, Surrey, UK), respectively. UV and IR spectra were acquired using a ThermoEVO 300 spectrometer (Thermo, Waltham, MA, USA) and a ThermoNicolet IS 10 spectrometer (Thermo, Waltham, MA, USA), respectively. NMR and mass spectra were recorded on a Bruker Avance III 500 spectrometer (Bruker, Germany) and a Bruker maXisHD mass spectrometer (Bruker, Germany), respectively. Preparative HPLC separations were performed on a SEP system (Beijing Sepuruisi scientific Co., Ltd, China) equipped with a variable-wavelength UV detector, using a YMC-Pack ODS-A column (250 × 20 mm, 5 μm). Monosaccharide isolation was conducted on a Waters 2695 separation module with an evaporative light scattering detector (ELSD) (Waters, Milford, MA, USA). MCI gel CHP-20, ODS gel (50 μm), sephadex LH-20 (40–70 μm), and silica gel (160–200 mesh) were acquired from TOSOH Corp., Tokyo, Japan, YMC Group, Kyoto, Japan, Amersham Pharmacia Biotech AB, Uppsala, Sweden, and Marine Chemical Industry, Qingdao, China, respectively. Chemical reagents for isolation were of analytical grade and purchased from Tianjin Siyou Co., Ltd, China. Biological reagents were from Sigma Company. Human hepatocellular carcinoma (SMMC-7721) cell line was bought from China Infrastructure of Cell Line Resources (Beijing, China), from Institute of Materia Medica, Chinese Academy of Medical Sciences and Peking Union Medical College, China. Human myeloid leukemia cell line (HL-60), lung cancer (A549), breast cancer (MCF-7), and colon cancer (SW-480) were from American Type Culture Collection (ATCC, Manassas, VA, USA).

### Plant material

The dried whole plants of *C. pareira* var. *hirsuta* were collected in Yunnan province, China, in July 2018, and authenticated by Prof. Cheng-Ming Dong at School of Pharmacy, Henan University of Chinese Medicine, where a voucher specimen (SE 20180705) was deposited.

### Extraction and isolation

The dried and powdered whole plants of *C. pareira* var. *hirsuta* (50.7 kg) were refluxed with 95% EtOH (3 × 300 L) to yield a crude extract (2.7 kg). The extract was dispersed in water (9 L) and sequentially partitioned with petroleum ether (PE, 9 L × 3), CH_2_Cl_2_ (9L × 3), and *n*-BuOH (3.2 L × 3) to afford the PE (351.1 g), the CH_2_Cl_2_ (740.1 g), and *n*-BuOH fractions (594.5 g). The CH_2_Cl_2_ fraction was separated into five fractions (A1–A5) by silica gel column chromatography (CC, 125 × 15 cm) with a gradient of PE (60–90 °C)–acetone (v/v 100 : 0, 100 : 1, 100 : 3, 100 : 5, 100 : 10, 100 : 30, 100 : 50, 1 : 1, 1 : 2). Fraction A4 (10.32 g) was chromatographed over open MCI gel CHP-20 CC (23 × 4 cm) eluted with a gradient of methanol–H_2_O (v/v 10 : 90, 30 : 70, 40 : 60, 70 : 30, 80 : 20) to yield five subfractions (A4-1–A4-5). Subfraction A4-2 (3.21 g) was passed through sephadex LH-20 CC (4.4 × 120 cm) eluted by MeOH to obtain six subfractions (A4-2-1–A4-2-6). The subfraction A4-2-2 (864.3 mg) was subjected to silica gel CC (35 × 2.5 cm) with a CH_2_Cl_2_ : MeOH (80 : 1, 50 : 1, 30 : 1, 20 : 1, 10 : 1, 0 : 1) gradient to give five subfractions (A4-2-2-1–A4-2-2-5). Subfraction A4-2-2-1 (350.0 mg) was purified by preparative HPLC (MeOH : H_2_O 65 : 35) to afford compounds 15 (18.8 mg, *t*_R_ 23.0 min), and 16 (14.9 mg, *t*_R_ 17.4 min). Subfraction A4-3 (2.17 g) was submitted to silica gel CC (45 × 5 cm) eluted by PE–EtOAC (10 : 1, 8 : 1, 5 : 1, 3 : 1, 2 : 1, 1 : 1, 1 : 2) to afford five subfractions (A4-3-1–A4-3-5). Subfraction A4-3-3 (936.2 mg) was further purified by preparative HPLC (CH_3_CN–H_2_O 50 : 50) to produce compounds 1 (10.0 mg, *t*_R_ 51.0 min), 8 (15.2 mg, *t*_R_ 45.3 min), and 11 (9.8 mg, *t*_R_ 71.2 min). Subfraction A4-4 (1.36 g) was subjected to silica gel CC (45 × 5 cm) eluted by PE–EtOAC (10 : 1, 8 : 1, 5 : 1, 3 : 1, 2 : 1, 1 : 1, 1 : 2) to obtain four subfractions (A4-4-1–A4-4-4). Subfraction A4-4-4 (686.6 mg) was rechromatographed by sephadex LH-20 CC (100 × 2.5 cm) eluted by MeOH to provide three subfractions (A4-4-4-1–A4-4-4-3). Compounds 4 (3.9 mg, *t*_R_ 31.9 min), 7 (12.5 mg, *t*_R_ 48.4 min), 9 (4.3 mg, *t*_R_31.2 min), 10 (8.9 mg, *t*_R_ 55.0 min), 12 (5.4 mg, *t*_R_ 51.8 min), and 13 (7.0 mg, *t*_R_ 63.1 min) were obtained from subfraction A4-4-4-2 (280.3 mg) using preparative HPLC (CH_3_CN–H_2_O, 60 : 40) at a flow rate of 6 mL min^−1^. Compounds 2 (18.9 mg, *t*_R_ 27.2 min), 17 (4.5 mg, *t*_R_ 42.3 min), and 18 (38.0 mg, *t*_R_ 38.4 min) were isolated from sub-fraction A4-4-4-3 (265.8 mg) using preparative HPLC (CH_3_CN–H_2_O 60 : 40) at a flow rate of 6 mL min^−1^. Subfraction A4-5 (3.32 g) was subjeced to silica gel CC (45 × 5 cm) eluted by PE–EtOAC (10 : 1, 8 : 1, 5 : 1, 3 : 1, 2 : 1, 1 : 1, and 1 : 2) to obtain three subfractions (A4-5-1–A4-5-3). Further separation of subfraction A4-5-2 (988.5 mg) using sephadex LH-20 CC (91 × 2.4 cm) eluted by MeOH resulted in five subfractions A4-5-2-1–A4-5-2-5. Subfraction A4-5-2-2 (334.2 mg) was purified by preparative HPLC eluted with MeOH–H_2_O (75 : 25) at a flow rate of 6 mL min^−1^ to give compounds 5 (23.0 mg, *t*_R_ 75.1 min), 6 (23.3 mg, *t*_R_108.2 min), and 14 (20.0 mg, *t*_R_ 88.4 min). Compounds 3 (50.0 mg, *t*_R_ 102.0 min) and 19 (19.8 mg, *t*_R_ 57.3 min) were isolated from subfraction A4-5-2-3 (349.8 mg) by preparative HPLC (MeOH–H_2_O 75 : 25) at a flow rate of 6 mL min^−1^.

#### Cissasteroid A (1)

White, amorphous powder; [*α*]^D^_20_ +56.1 (*c* 0.02, MeOH); UV (MeOH) *λ*_max_ (log *ε*) 207 (3.88), 216 (3.81), 276 (3.92), 377 (1.42) nm; IR (iTR)*ν*_max_ 3398, 2933, 1703, 1639, 1451, 1376, 1311, 1278, 1187, 1082, 1029 cm^−1^; HR-ESI-MS (positive): *m*/*z* 679.3451 [M + Na]^+^ (calcd for C_37_H_52_O_10_Na, 679.3458); NMR data (CD_3_OD), see Tables 1 and 2.

#### Cissasteroid B (2)

White, amorphous powder; [*α*]^D^_20_ +18.2 (*c* 0.08, MeOH); UV (MeOH) *λ*_max_ (log *ε*) 205 (3.64), 231 (3.75), 274 (2.93) nm; IR (iTR)*ν*_max_ 3395, 2934, 1708, 1452, 1382, 1316, 1277, 1163, 1074, 1027 cm^−1^; HR-ESI-MS (positive): *m*/*z* 653.3304 [M + Na]^+^ (calcd for C_35_H_50_O_10_Na, 653.3302); NMR data (CD_3_OD), see Tables 1 and 2.

#### Cissasteroid C (3)

White, amorphous powder; [*α*]^D^_20_ +77.0 (*c* 0.02, MeOH); UV (MeOH) *λ*_max_ (log *ε*) 224 (4.09), 281 (4.04) nm; IR (iTR)*ν*_max_ 3365, 2939, 1705, 1638, 1451, 1311, 1278, 1165, 1075, 1025 cm^−1^; HR-ESI-MS (positive): *m*/*z* 783.3720 [M + Na]^+^ (calcd for C_44_H_56_O_11_Na, 783.3720); NMR data (CD_3_OD), see Table 1 and 2.

#### Cissasteroid D (4)

White, amorphous powder; [*α*]^D^_20_ +46.5 (*c* 0.05, MeOH); UV (MeOH) *λ*_max_ (log *ε*) 207 (3.84), 216 (3.83), 276 (3.86) nm; IR (iTR)*ν*_max_ 3418, 2931, 1704, 1638, 1451, 1368, 1311, 1280, 1165, 1084, 1061 cm^−1^; HR-ESI-MS (positive): *m*/*z* 823.4244 [M + Na]^+^ (calcd for C_44_H_64_O_13_Na, 823.4245); NMR data (CD_3_OD), see Tables 1 and 2.

#### Cissasteroid E (5)

White, amorphous powder; [*α*]^D^_20_ +12.4 (*c* 0.05, MeOH); UV (MeOH) *λ*_max_ (log *ε*) 207 (3.84), 218 (3.86), 280 (3.99), 375 (1.35) nm; IR (iTR)*ν*_max_ 3384, 2935, 1702, 1636, 1578, 1451, 1368, 1312, 1280, 1165, 1081, 1059, 1027 cm^−1^; HR-ESI-MS (positive): *m*/*z* 823.4244 [M + Na]^+^ (calcd for C_44_H_64_O_13_Na, 823.4245); NMR data (CD_3_OD), see Tables 1 and 2.

#### Cissasteroid F (6)

White, amorphous powder; [*α*]^D^_20_ +58.1 (*c* 0.12, MeOH); UV (MeOH) *λ*_max_ (log *ε*) 206 (4.02), 230 (4.18), 275 (3.21), 376 (1.78) nm; IR (iTR)*ν*_max_ 3405, 2933, 1711, 1602, 1451, 1307, 1316, 1277, 1078, 1026 cm^−1^; HR-ESI-MS (positive): *m*/*z* 901.4350 [M + Na]^+^ (calcd for C_49_H_66_O_14_Na, 901.4350); NMR data (CD_3_OD), see Tables 1 and 2.

#### Cissasteroid G (7)

White, amorphous powder; [*α*]^D^_20_ −10.9 (*c* 0.03, MeOH); UV (MeOH) *λ*_max_ (log *ε*) 207 (2.80), 228 (3.95), 274 (3.05) nm; IR (iTR)*ν*_max_ 3379, 2934, 1708, 1602, 1451, 1369, 1316, 1277, 1194, 1164, 1146, 1081, 1027 cm^−1^; HR-ESI-MS (positive): *m*/*z* 797.4087 [M + Na]^+^ (calcd for C_42_H_62_O_13_Na, 797.4088); NMR data (CD_3_OD), see Tables 1 and 2.

#### Cissasteroid H (8)

White, amorphous powder; [*α*]^D^_20_ +9.8 (*c* 0.04, MeOH); UV (MeOH) *λ*_max_ (log *ε*) 219 (2.89) nm; IR (iTR)*ν*_max_ 3409, 2935, 1690, 1648, 1454, 1380, 1267, 1194, 1161, 1142, 1078, 1030 cm^−1^; HR-ESI-MS (positive): *m*/*z* 631.3458 [M + Na]^+^ (calcd for C_33_H_52_O_10_Na, 631.3458); NMR data (CD_3_OD), see Tables 1 and 2.

#### Cissasteroid I (9)

White, amorphous powder; [*α*]^D^_20_–10.9 (*c* 0.05, MeOH); UV (MeOH) *λ*_max_ (log *ε*) 207 (3.86) nm; IR (iTR)*ν*_max_ 3395, 2932, 1703, 1649, 1451, 1377, 1268, 1194, 1149, 1060, 1029 cm^−1^; HR-ESI-MS (positive): *m*/*z* 775.4244 [M + Na]^+^ (calcd for C_40_H_64_O_13_Na, 775.4245); NMR data (CD_3_OD), see Tables 1 and 2.

#### Cissasteroid J (10)

White, amorphous powder; [*α*]^D^_20_ +8.9 (*c* 0.26, MeOH); UV (MeOH) *λ*_max_ (log *ε*) 216 (3.93) nm; IR (iTR)*ν*_max_ 3432, 2933, 1702, 1650, 1453, 1369, 1318, 1266, 1195, 1149, 1082, 1058, 1031 cm^−1^; HR-ESI-MS (positive): *m*/*z* 919.5031 [M + Na]^+^ (calcd for C_47_H_76_O_16_Na, 919.5031); NMR data (CD_3_OD), see Tables 1 and 2.

#### Cissasteroid K (11)

White, amorphous powder; [*α*]^D^_20_ +3.6 (*c* 0.07, MeOH); UV (MeOH) *λ*_max_ (log *ε*) 215 (4.25) nm; IR (iTR)*ν*_max_ 3409, 2933, 1730, 1701, 1650, 1452, 1374, 1265, 1237, 1146, 1076, 1027 cm^−1^; HR-ESI-MS (positive): *m*/*z* 673.3565 [M + Na]^+^ (calcd for C_35_H_54_O_11_Na, 673.3564); NMR data (CD_3_OD), see Tables 1 and 2.

#### Cissasteroid L (12)

White, amorphous powder; [*α*]^D^_20_ +13.9 (*c* 0.05, MeOH); UV (MeOH) *λ*_max_ (log *ε*) 209 (3.92) nm; IR (iTR)*ν*_max_ 3378, 2935, 1732, 1703, 1650, 1451, 1373, 1267, 1238, 1194, 1149, 1065, 1028 cm^−1^; HR-ESI-MS (positive): *m*/*z* 817.4350 [M + Na]^+^ (calcd for C_42_H_66_O_14_Na, 817.4350); NMR data (CD_3_OD), see Tables 1 and 2.

#### Cissasteroid M (13)

White, amorphous powder; [*α*]^D^_20_ +23.6 (*c* 0.40, MeOH); UV (MeOH) *λ*_max_ (log *ε*) 215 (3.72) nm; IR (iTR)*ν*_max_ 3387, 2934, 1731, 1702, 1650, 1451, 1371, 1319, 1266, 1237, 1194, 1162, 1147, 1079, 1027 cm^−1^; HR-ESI-MS (positive): *m*/*z* 817.4360 [M + Na]^+^ (calcd for C_42_H_66_O_14_Na, 817.4350); NMR data (CD_3_OD), see Tables 1 and 2.

#### Cissasteroid N (14)

White, amorphous powder; [*α*]^D^_20_ +20.5 (*c* 0.26, MeOH); UV (MeOH) *λ*_max_ (log *ε*) 212 (3.86) nm; IR (iTR)*ν*_max_ 3382, 2935, 1732, 1703, 1649, 1451, 1371, 1318, 1267, 1238, 1194, 1150, 1058, 1027 cm^−1^; HR-ESI-MS (positive): *m*/*z* 961.5137 [M + Na]^+^ (calcd for C_49_H_78_O_17_Na, 961.5137); NMR data (CD_3_OD), see Tables 1 and 2.

### Absolute configuration determination of sugar moieties

A solution of 10 (2.2 mg) in 1 mL of MeOH was hydrolyzed with 100 μL of 0.05 N H_2_SO_4_. The solution was stirred at 60 °C for 2 h. After cooling, the reation mixture was diluted with 10 mL of H_2_O and extracted with 10 mL of CH_2_Cl_2_. The H_2_O phase was neutralized with saturated aqueous Ba(OH)_2_ solution. The precipitate was filtered off, and then the filtrate was evaporated under reduced pressure to give the sugar fraction. The residue was isolated by preparative HPLC-ELSD (a TSKgel G3000PWXL column, 300 mm × 7.8 mm, 5 μm) and eluted with H_2_O at a flow of 0.2 mL min^−1^ to obtain oleandropyranose (*t*_R_ 53.9 min) and cymaropyranose (*t*_R_ 55.3 min), respectively. Compounds 1–9, and 11–14 were hydrolyzed by the above procedure. d-Oleandrose and d-cymarose were identified by comparing their experimental and reported rotation values.^16^

### Cytotoxicity assay *in vitro*

Cytotoxicity was tested by the MTS method previously described.^25^ Cisplatin and paclitaxel were used as positive control. The cytotoxicity of compounds 1–19 was evaluated against HL-60, A-549, SMMC-7721, MCF-7, and SW-480 cell lines. All the cells were cultured in RPMI-1640 medium, supplemented with 10% fetal bovine serum at 37 °C in a humidified atmosphere with 5% CO_2_. Cell viability was assessed by conducting colorimetric measurements of the amount of insoluble formazan formed in living cells based on the reduction of 3-(4,5-dimethylthiazol-2-yl)-5-(3-carboxymethoxyphenyl)-2-(4-sulfophenyl)-2*H*-tetrazolium (MTS). To be brief, 100 μL of adherent cells were seeded into each well of a 96-well cell culture plate and allowed to adhere for 24 h before drug addition, each tumor cell line was exposed to the test compound at various concentrations in triplicate for 48 h. After the incubation, MTS (20 μL) was added to each well, and the incubation continued for 4 h at 37 °C. The optical density of each well was measured at 492 nm in a 96-well microtiter plate reader. The IC_50_ value of each compound was calculated by the Reed–Muench's method.

### NO inhibitory activity

The NO inhibitory activity was evaluated by the previously reported protocol.^25^l-*N*^G^-Monomethyl arginine (l-NMMA) was used as a positive control. RAW 264.7 macrophages cells (2 × 10^5^ cells per well) were precultured in 96-well microplates for 24 h. The test compounds (50 μM) and l-NMMA with 1 μg mL^−1^ LPS were added and incubated for another 18 h at 37 °C. Nitric oxide production was assessed by the Griess Reagent System.

## Author contributions

H. J. Chen and H. Chen performed the experiments, data analysis, and experimental planning. Y. Y. Si, M. Li, and K. Du screened the biological activities. The project was conceived and supervised by Y. J. Sun and W. S. Feng. The manuscript was written by Y. J. Sun, R. J. Han, and C. Zhao. All authors reviewed the manuscript.

## Conflicts of interest

There are no conflicts to declare.

## Supplementary Material

RA-012-D1RA07498A-s001
