# Supplementary material for: Cytotoxic polyhydroxylated pregnane glycosides from Cissampelos pareira var. hirsuta
Source: RSC Adv. 2021 Dec 22;12(1):498–508. doi: 10.1039/d1ra07498a (PMC8693877; doi:10.1039/d1ra07498a)
Supplement: RA-012-D1RA07498A-s001 [file RA-012-D1RA07498A-s001.pdf]

Electronic Supplementary Material (ESI) for RSC Advances.
This journal is © The Royal Society of Chemistry 2021

Cytotoxic polyhydroxylated pregnane glycosides from *Cissampelos pareira* var. *hirsuta*

Yan-Jun Sun,[*abc] Hao-Jie Chen,[ab] Rui-Jie Han,[ab] Chen Zhao,[ab] Ying-Ying Si,[ab] Meng Li,[ab] Kun Du,[ab] Hui Chen,[ab] Wei-Sheng Feng[*ab]

Fourteen new polyhydroxylated pregnane glycosides, cissasteroid A−N (**1**−**14**), and five known analogues (**15**−**19**), were isolated from the dried whole plant of *Cissampelos pareira* var. *hirsuta*. Their structures and stereochemistry were elucidated by extensive spectroscopic data, chemical hydrolysis, and ECD measurements. All the compounds were tested for their cytotoxicity against five human cancer cell lines, and inhibitory activity against NO release in LPS-induced RAW 264.7 cells. Compared with cisplatin, compound **7** showed more potent cytotoxicities against the HL-60, A549, SMMC-7721, MCF-7, and SW480 cell lines, with IC_{50} values of 2.19, 14.38, 2.00, 7.58, and 7.44 μM, respectively. The preliminary study of structure-activity relationship indicated that benzoic acid esterification at C-20 may have a negative effect on the cytotoxic activity of polyhydroxylated pregnane derivatives in these five human cancer cell lines. These results revealed the potential of compound **7** as an ideal antitumor lead compound.

[a]*Co-construction Collaborative Innovation Center for Chinese Medicine and Respiratory Diseases by Henan & Education Ministry of P. R. China, Henan University of Traditional Chinese Medicine, Zhengzhou 450046, P. R. China. E-mails: sunyanjunily@126.com; fwsh@hactcm.edu.cn*

[b]*School of Pharmacy, Henan University of Traditional Chinese Medicine, Zhengzhou 450046, P. R. China*

[c]*Henan Research Center for Special Processing Technology of Chinese Medicine, Zhengzhou, 450046, P. R. China*

Table of Contents

Figure S1. ^{1}H NMR spectrum (500 MHz) of **1** in CD_3OD 5
Figure S2. ^{13}C NMR spectrum (125 MHz) of **1** in CD_3OD 5
Figure S3. DEPT spectrum of **1** in CD_3OD 6
Figure S4. ^{1}H-^{1}H COSY of **1** in CD_3OD 6
Figure S5. HSQC of **1** in CD_3OD 7
Figure S6. HMBC of **1** in CD_3OD 7
Figure S8. NOESY of **1** in CD_3OD 8
Figure S9. ^{1}H NMR spectrum (500 MHz) of **2** in CD_3OD 8
Figure S10. ^{13}C NMR spectrum (125 MHz) of **2** in CD_3OD 9
Figure S11. DEPTspectrum of **2** in CD_3OD 9
Figure S12. ^{1}H-^{1}H COSY of **2** in CD_3OD 10
Figure S13. HSQC of **2** in CD_3OD 10
Figure S14. HMBC of **2** in CD_3OD 11
Figure S15. NOESY of **2** in CD_3OD 11
Figure S16. ^{1}H NMR spectrum (500 MHz) of **3** in CD_3OD 12
Figure S17. ^{13}C NMR spectrum (500 MHz) of **3** in CD_3OD 12
Figure S18. DEPTspectrum of **3** in CD_3OD 13
Figure S19. ^{1}H-^{1}H COSY of **3** in CD_3OD 13
Figure S20. HSQC of **3** in CD_3OD 14
Figure S21. HMBC of **3** in CD_3OD 14
Figure S21. NOESY of **3** in CD_3OD 15
Figure S22. ^{1}H NMR spectrum (500 MHz) of **4** in CD_3OD 15
Figure S23. ^{13}C NMR spectrum (125 MHz) of **4** in CD_3OD 16
Figure S24. DEPT spectrum of **4** in CD_3OD 16
Figure S25. ^{1}H-^{1}H COSY of **4** in CD_3OD 17
Figure S26. HSQC of **4** in CD_3OD 17
Figure S27. HMBC of **4** in CD_3OD 18
Figure S28. NOESY of **4** in CD_3OD 18
Figure S29. ^{1}H NMR spectrum (500 MHz) of **5** in CD_3OD 19
Figure S30. ^{13}C NMR spectrum (125 MHz) of **5** in CD_3OD 19
Figure S31. DEPT spectrum of **5** in CD_3OD 20
Figure S32. ^{1}H-^{1}H COSY of **5** in CD_3OD 20
Figure S33. HSQC of **5** in CD_3OD 21
Figure S34. HMBC of **5** in CD_3OD 21
Figure S35. NOESY of **5** in CD_3OD 22
Figure S36. ^{1}H NMR spectrum (500 MHz) of **6** in CD_3OD 22
Figure S37. ^{13}C NMR spectrum (125 MHz) of **6** in CD_3OD 23
Figure S38. DEPTspectrum of **6** in CD_3OD 23
Figure S39. ^{1}H-^{1}H COSY of **6** in CD_3OD 24
Figure S40. HSQC of **6** in CD_3OD 24
Figure S41. HMBC of **6** in CD_3OD 25
Figure S42. NOESY of **6** in CD_3OD 25
Figure S43. ^{1}H NMR spectrum (500 MHz) of **7** in $CDCl_3$ 26

Figure S44. ^{13}C NMR spectrum (125 MHz) of **7** in $CDCl_3$ 26
Figure S45. DEPT spectrum of **7** in $CDCl_3$ 27
Figure S46. ^{1}H-^{1}H COSY of **7** in $CDCl_3$ 27
Figure S47. HSQC of **7** in $CDCl_3$ 28
Figure S48. HMBC of **7** in $CDCl_3$ 28
Figure S49. NOESY of **7** in $CDCl_3$ 29
Figure S50. ^{1}H NMR spectrum (500 MHz) of **8** in CD_3OD 29
Figure S51. ^{13}C NMR spectrum (125 MHz) of **8** in CD_3OD 30
Figure S52. DEPTspectrum of **8** in CD_3OD 30
Figure S53. ^{1}H-^{1}H COSY of **8** in CD_3OD 31
Figure S54. HSQC of **8** in CD_3OD 31
Figure S55. HMBC of **8** in CD_3OD 32
Figure S56. NOESY of **8** in CD_3OD 32
Figure S57. ^{1}H NMR spectrum (500 MHz) of **9** in CD_3OD 33
Figure S58. ^{13}C NMR spectrum (125 MHz) of **9** in CD_3OD 33
Figure S59. DEPTspectrum of **9** in CD_3OD 34
Figure S60.^{1}H-^{1}H COSY of **9** in CD_3OD 34
Figure S61. HSQC of **9** in CD_3OD 35
Figure S62. HMBC of **9** in CD_3OD 35
Figure S63. NOESY of **9** in CD_3OD 36
Figure S64. ^{1}H NMR spectrum (500 MHz) of **10** in CD_3OD 36
Figure S65. ^{13}C NMR spectrum (125 MHz) of **10** in CD_3OD 37
Figure S66. DEPTspectrum of **10** in CD_3OD 37
Figure S67. ^{1}H-^{1}H COSY of **10** in CD_3OD 38
Figure S68. HSQC of **10** in CD_3OD 38
Figure S69. HMBC of **10** in CD_3OD 39
Figure S70. NOESY of **10** in CD_3OD 39
Figure S71. ^{1}H NMR spectrum (500 MHz) of **11** in CD_3OD 40
Figure S72. ^{13}C NMR spectrum (125 MHz) of **11** in CD_3OD 40
Figure S73. DEPTspectrum of **11** in CD_3OD 41
Figure S74. ^{1}H-^{1}H COSY of **11** in CD_3OD 41
Figure S75. HSQC of **11** in CD_3OD 42
Figure S76. HMBC of **11** in CD_3OD 42
Figure S77. NOESY of **11** in CD_3OD 43
Figure S78. ^{1}H NMR spectrum (500 MHz) of **12** in CD_3OD 43
Figure S79.^{13}C NMR spectrum (125 MHz) of **12** in CD_3OD 44
Figure S80. DEPT spectrum of **12** in CD_3OD 44
Figure S81. ^{1}H-^{1}H COSY of **12** in CD_3OD 45
Figure S82. HSQC of **12** in CD_3OD 45
Figure S83. HMBC of **12** in CD_3OD 46
Figure S84. NOESY of **12** in CD_3OD 46
Figure S85. ^{1}H NMR spectrum (500 MHz) of **13** in CD_3OD 47
Figure S86. ^{13}C NMR spectrum (125 MHz) of **13** in CD_3OD 47
Figure S87. DEPT spectrum of **13** in CD_3OD 48

Figure S88. ^{1}H-^{1}H COSY of **13** in CD_3OD 48
Figure S89. HSQC of **13** in CD_3OD 49
Figure S90. HMBC of **13** in CD_3OD 49
Figure S91. NOESY of **13** in CD_3OD....50
Figure S92. ^{1}H NMR spectrum (500 MHz) of **14** in CD_3OD 50
Figure S93. ^{13}C NMR spectrum (125 MHz) of **14** in CD_3OD 51
Figure S94. DEPTspectrum of **14** in CD_3OD 51
Figure S95. ^{1}H-^{1}H COSY of **14** in CD_3OD 52
Figure S96. HSQC of **14** in CD_3OD 52
Figure S97. HMBC of **14** in CD_3OD 53
Figure S98. NOESY of **14** in CD_3OD....53

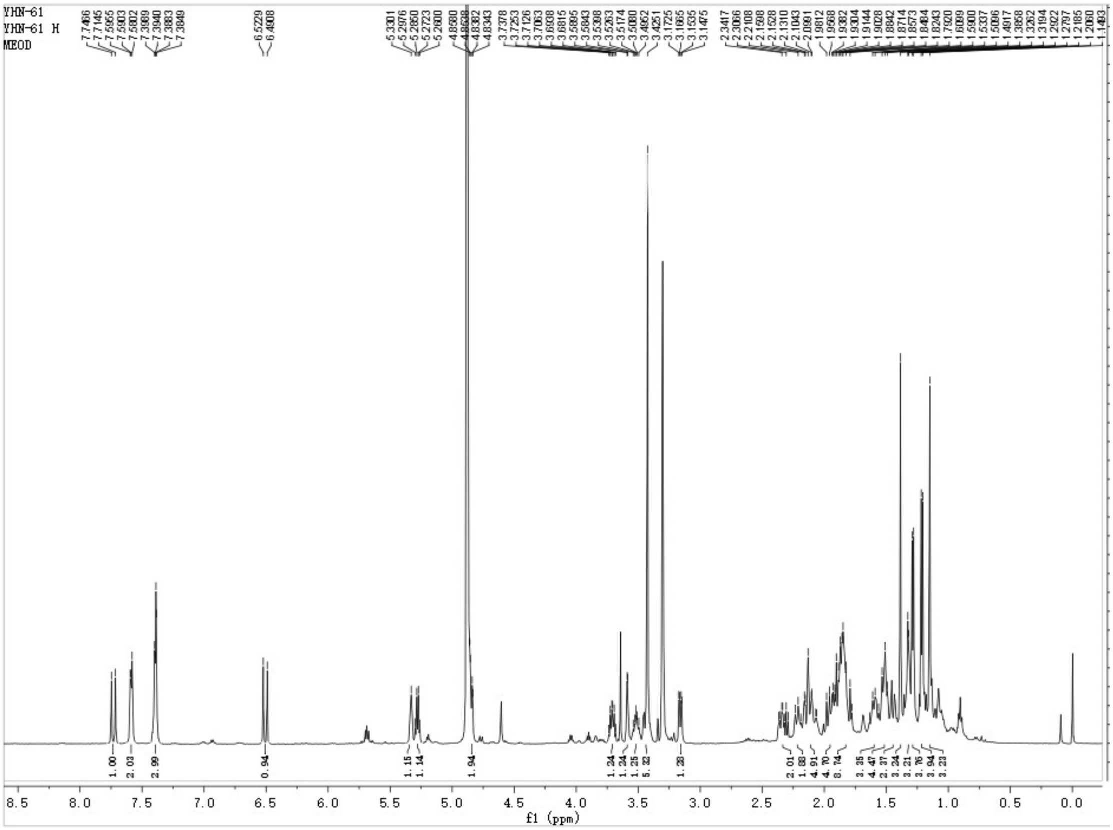

Figure S1. ^{1}H NMR spectrum (500 MHz) of **1** in CD_3OD

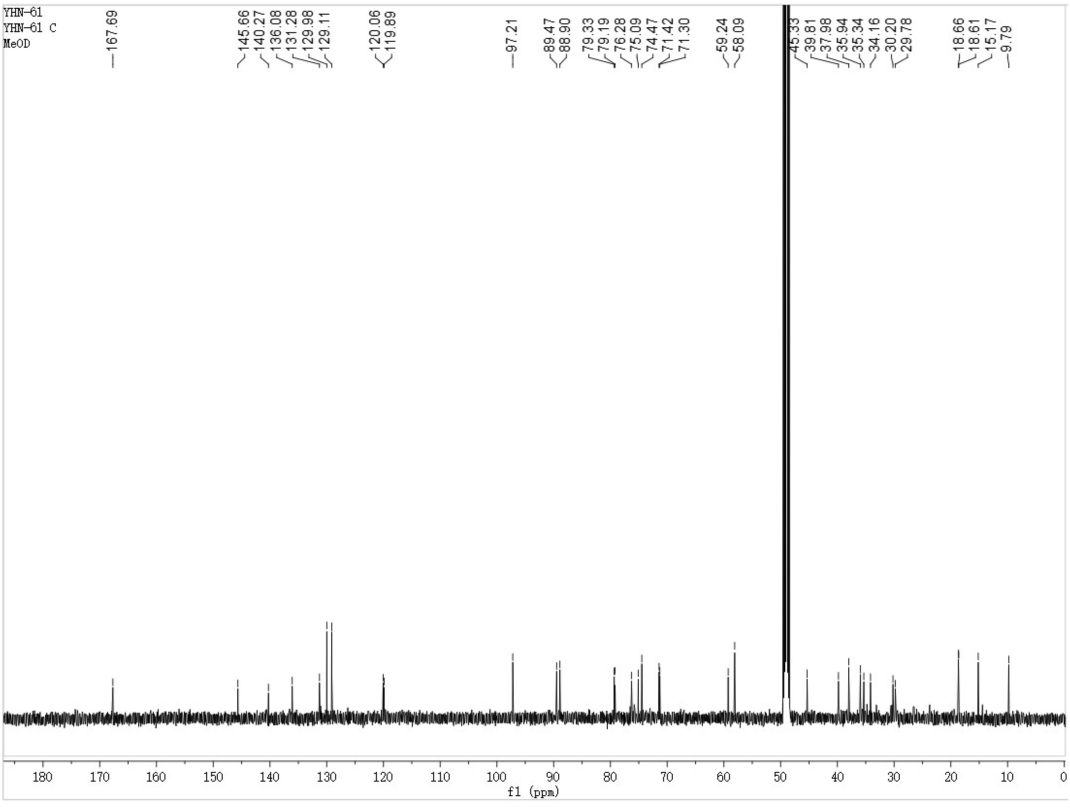

Figure S2. ^{13}C NMR spectrum (125 MHz) of **1** in CD_3OD

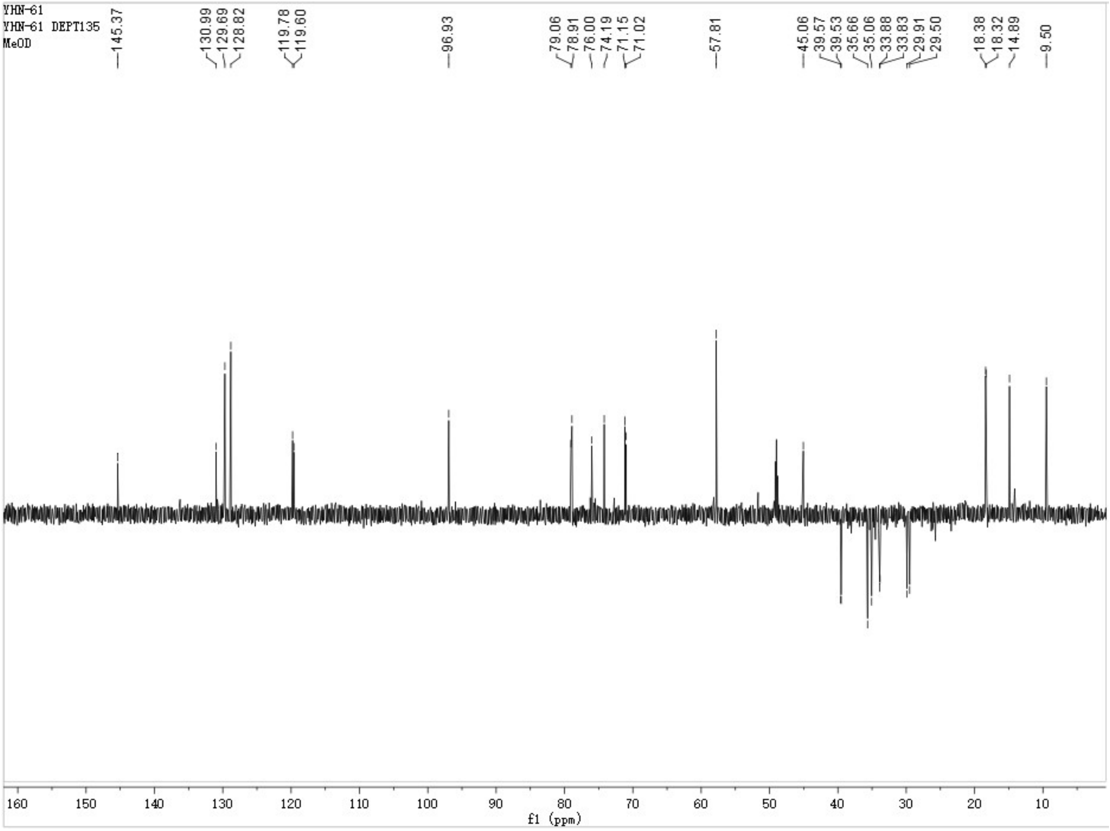

Figure S3. DEPT spectrum of **1** in CD_3OD

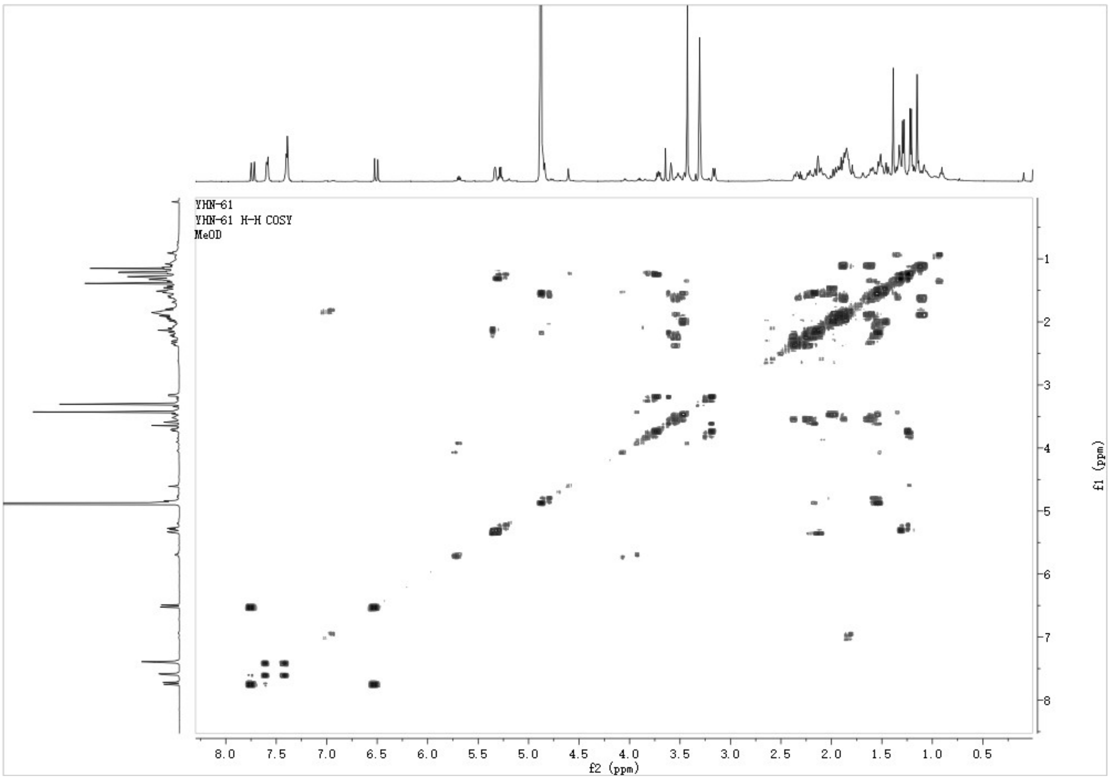

Figure S4. ^1H-^1H COSY of **1** in CD_3OD

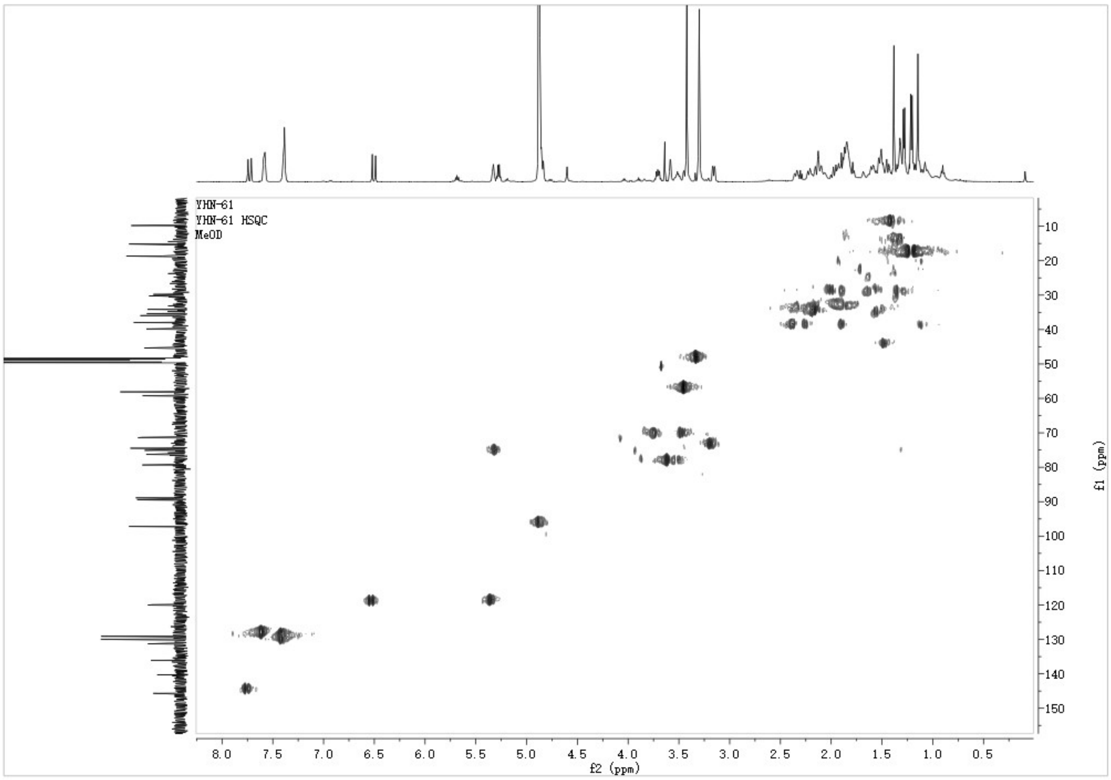

Figure S5. HSQC of **1** in CD_3OD

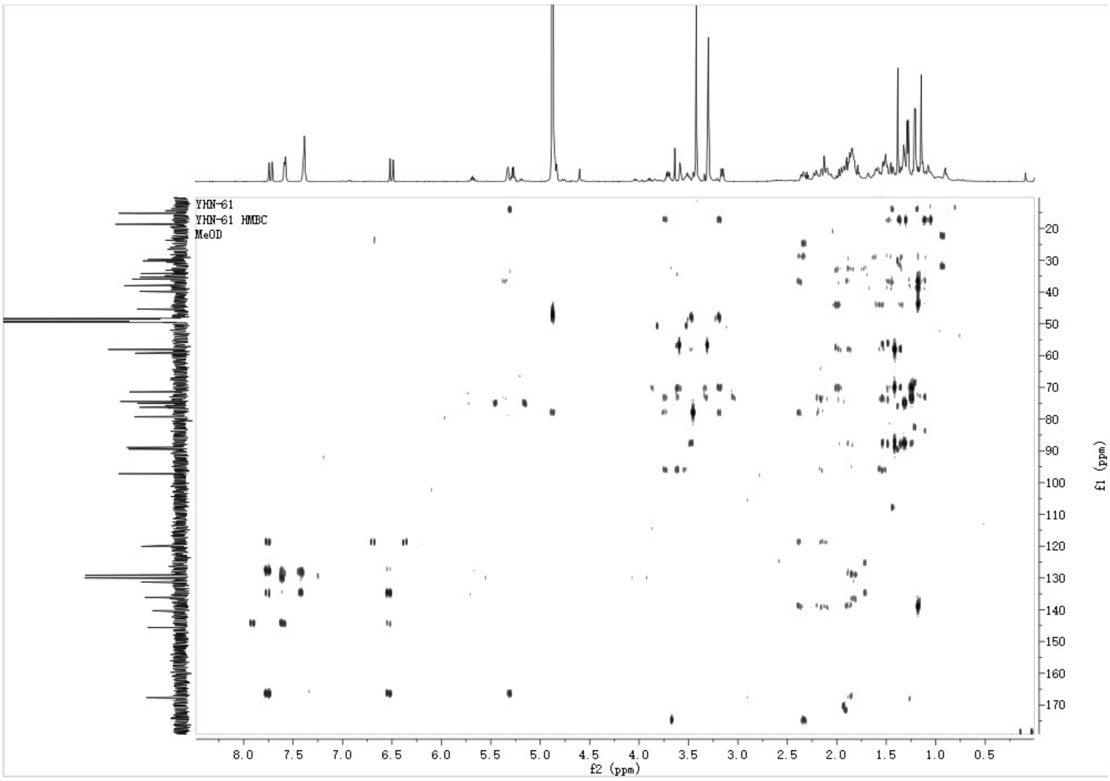

Figure S6. HMBC of **1** in CD_3OD

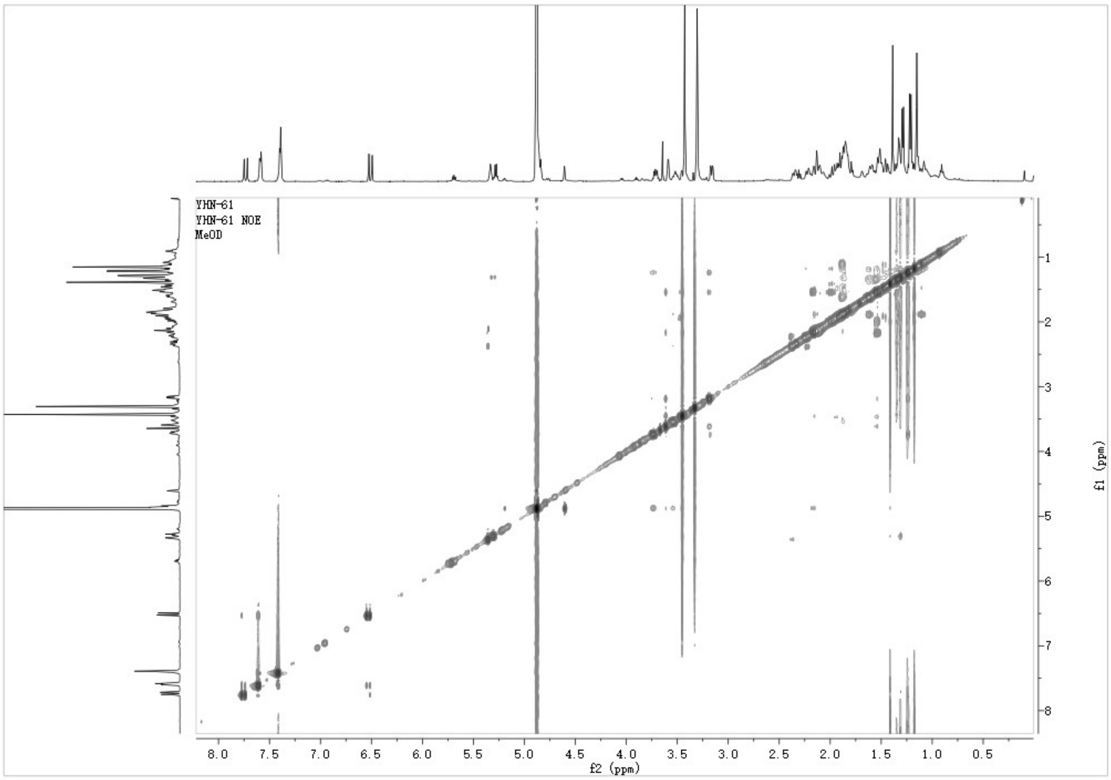

Figure S7. NOESY of **1** in CD_3OD

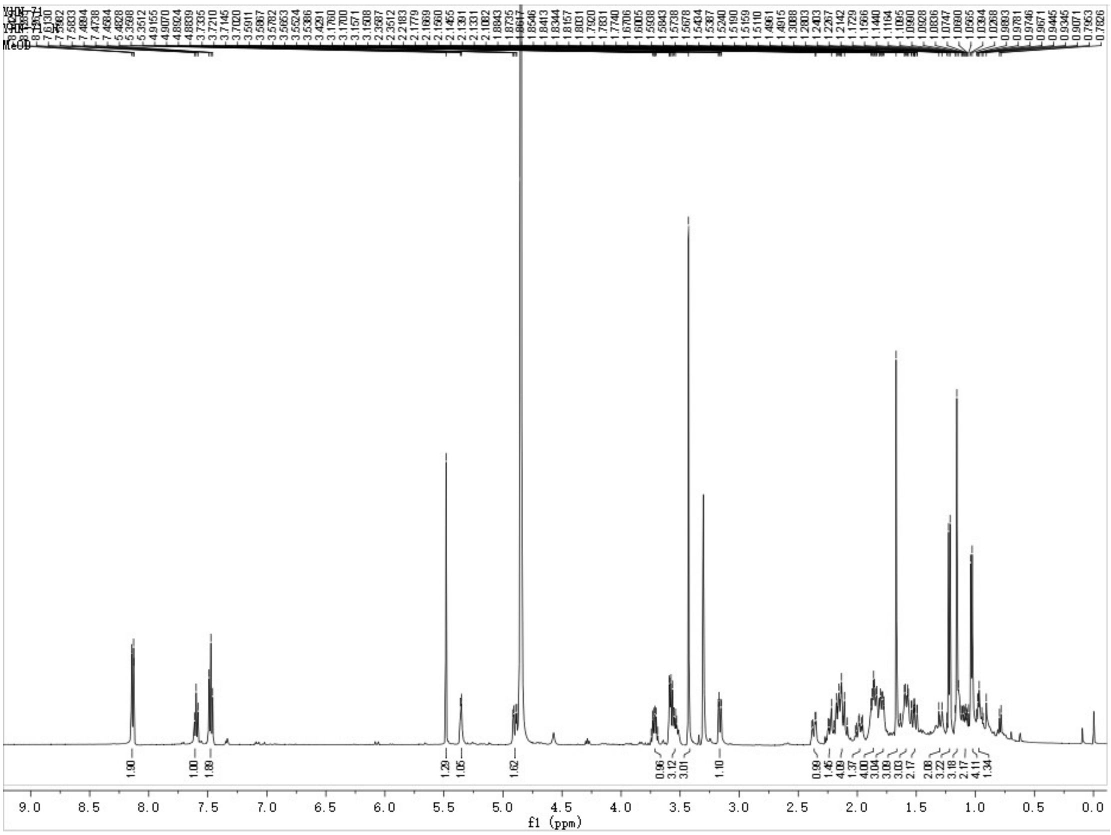

Figure S8. ^{1}H NMR spectrum (500 MHz) of **2** in CD_3OD

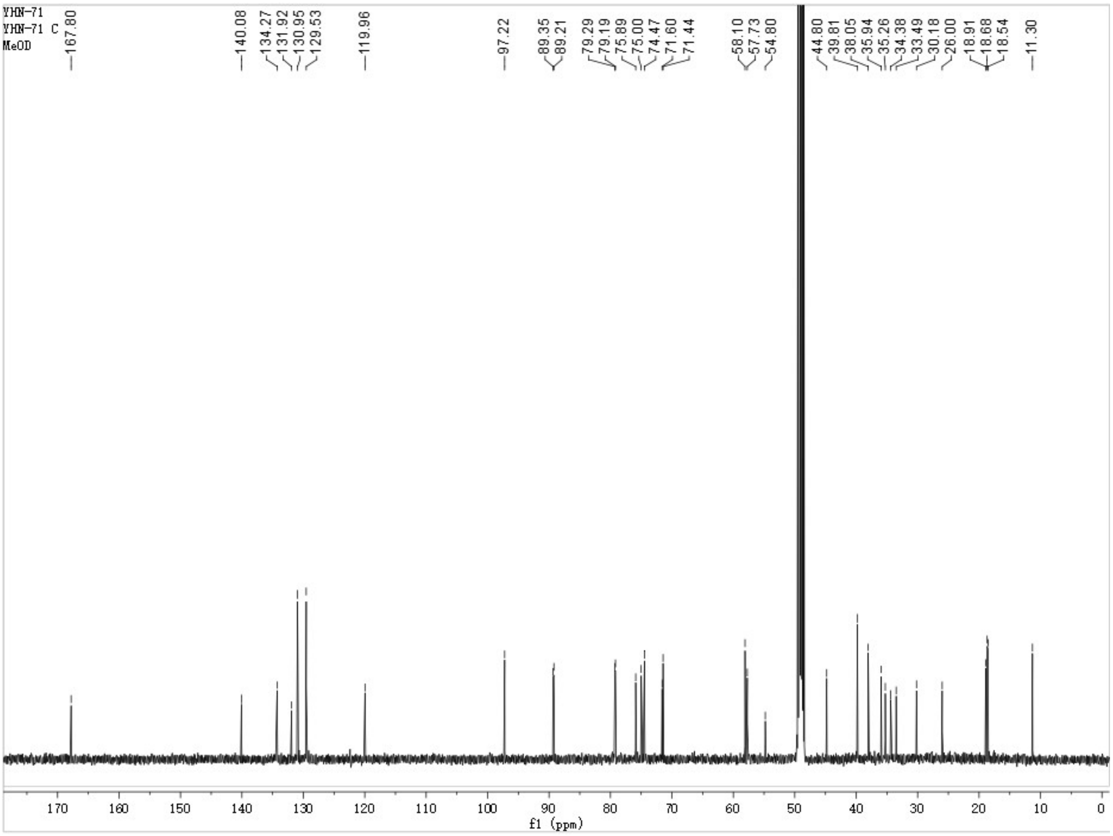

Figure S9. ^{13}C NMR spectrum (125 MHz) of **2** in CD_3OD

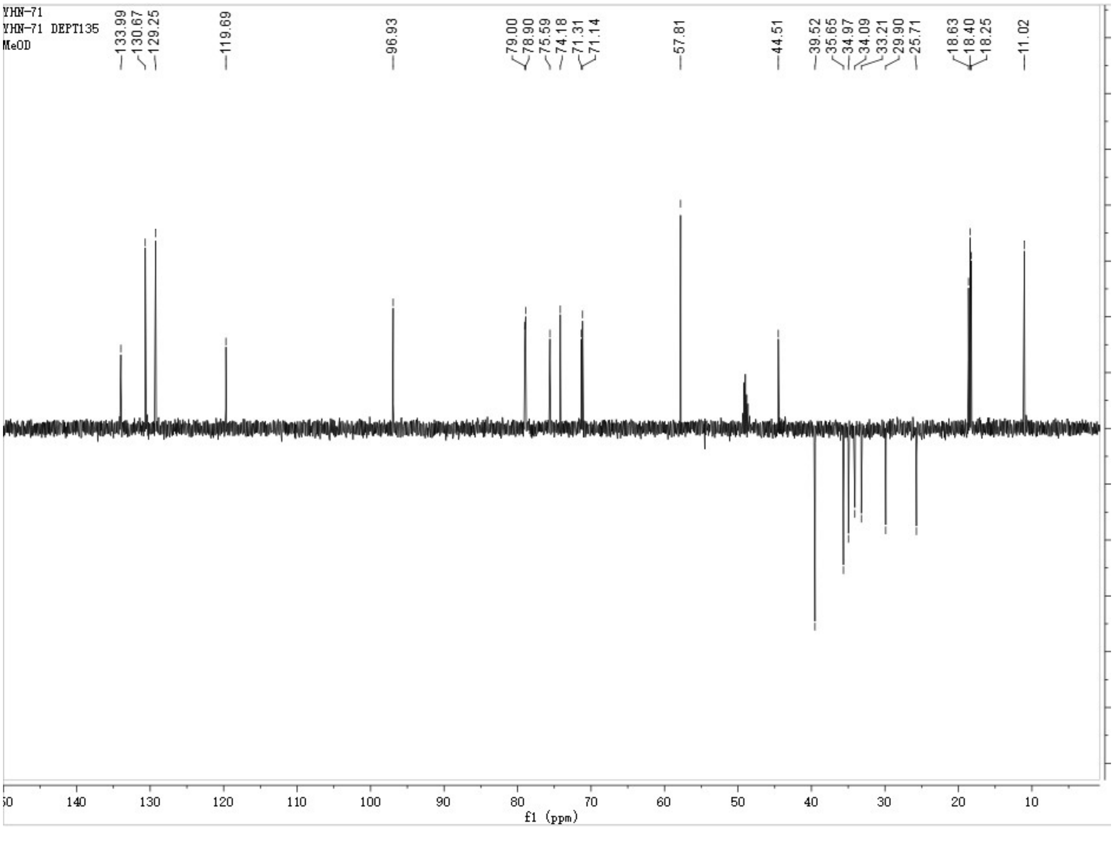

Figure S10. DEPT spectrum of **2** in CD_3OD

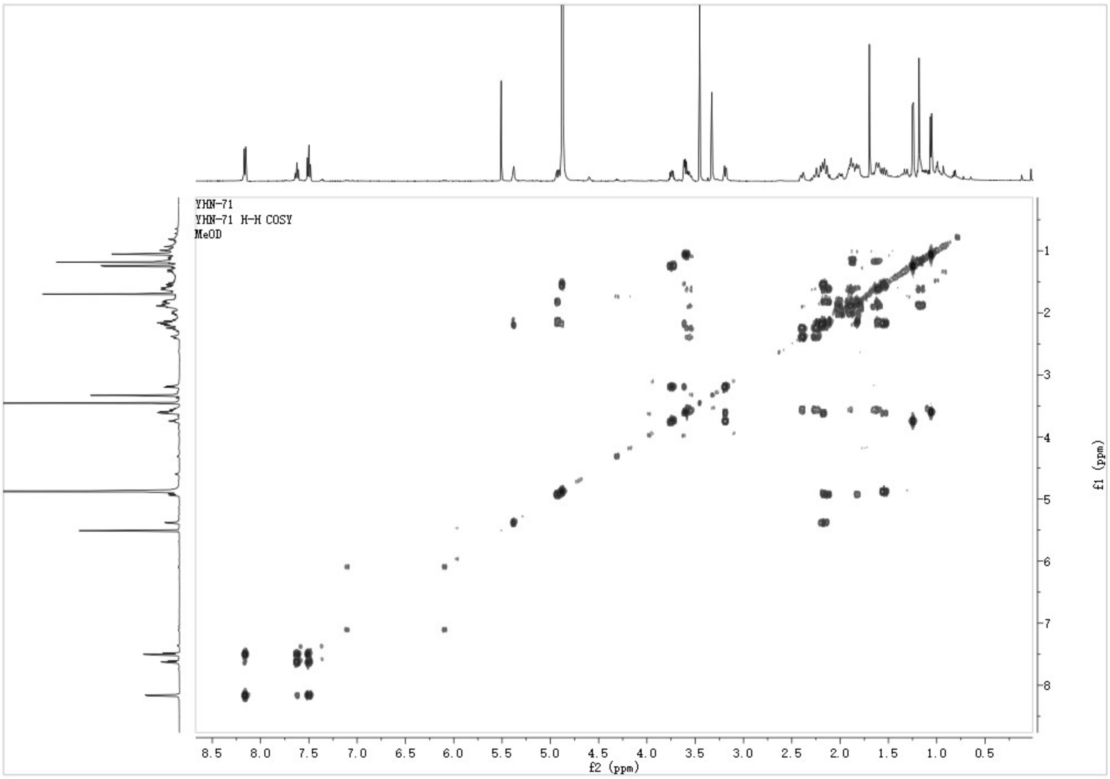

Figure S11. ^{1}H-^{1}H COSY of **2** in CD_3OD

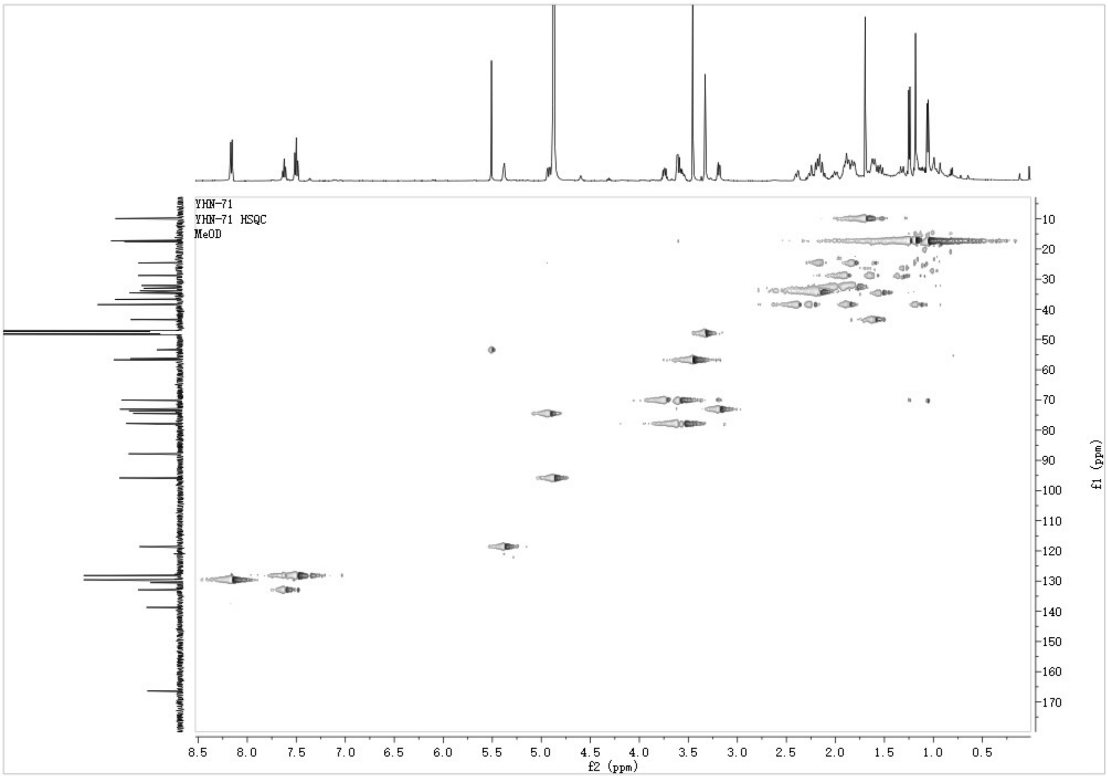

Figure S12. HSQC of **2** in CD_3OD

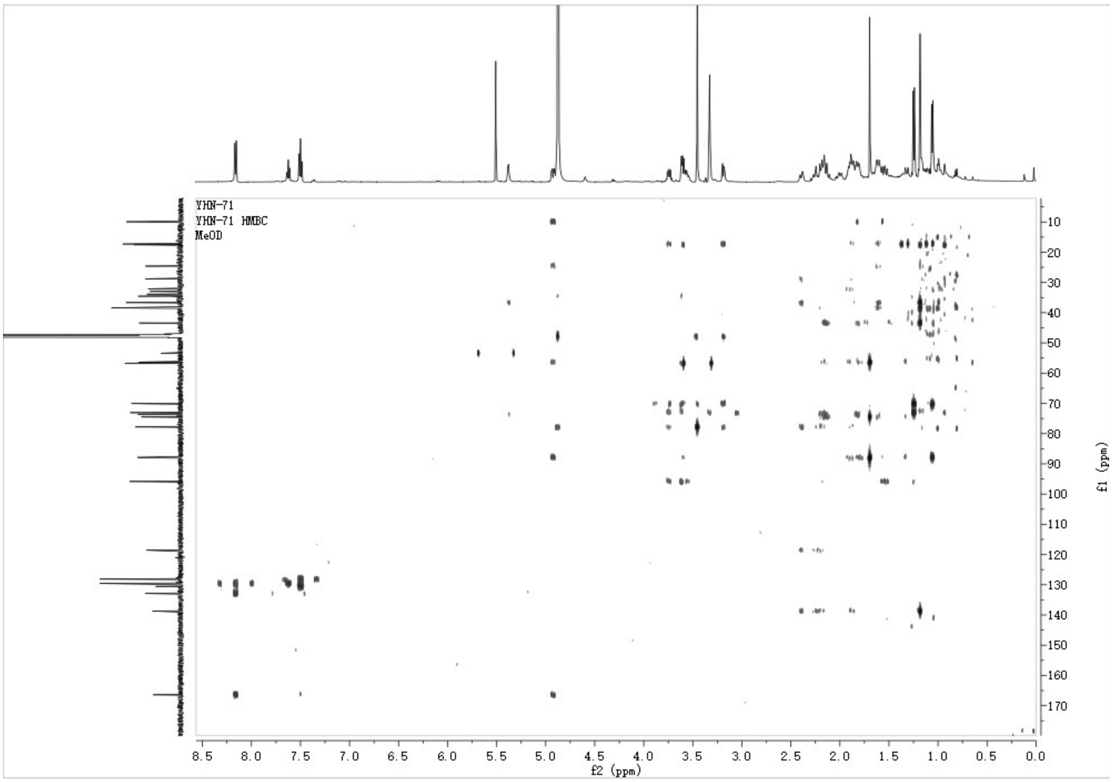

Figure S13. HMBC of **2** in CD_3OD

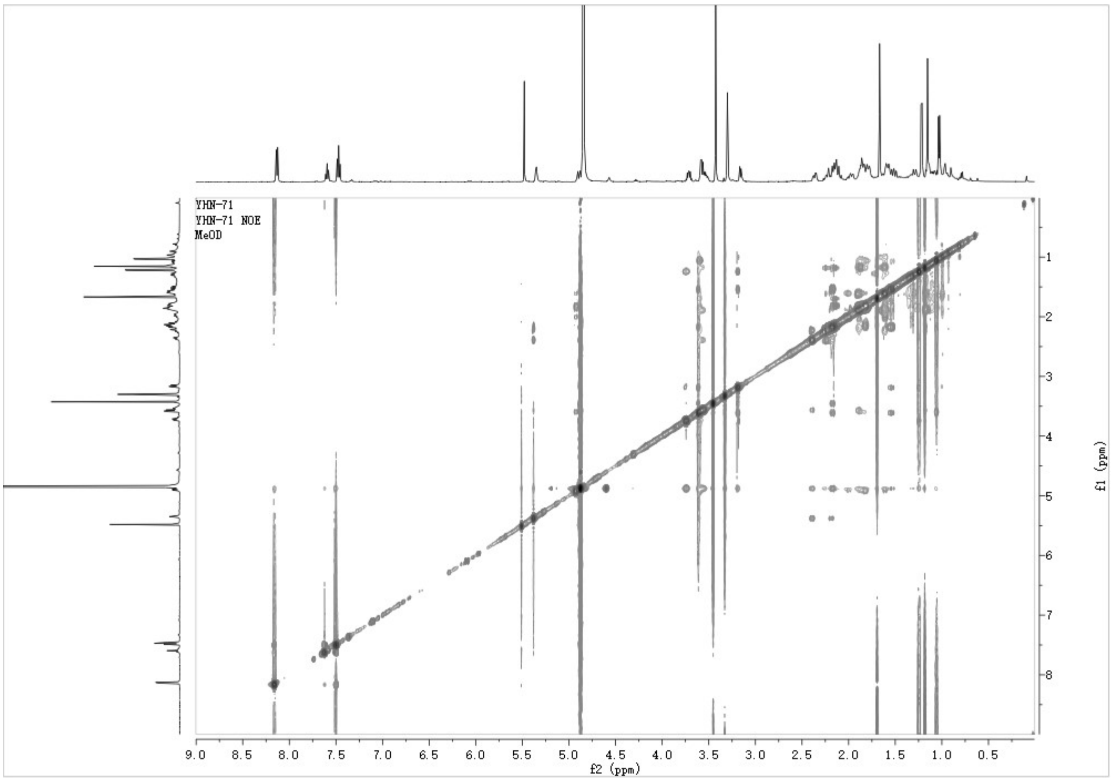

Figure S14. NOESY of **2** in CD_3OD

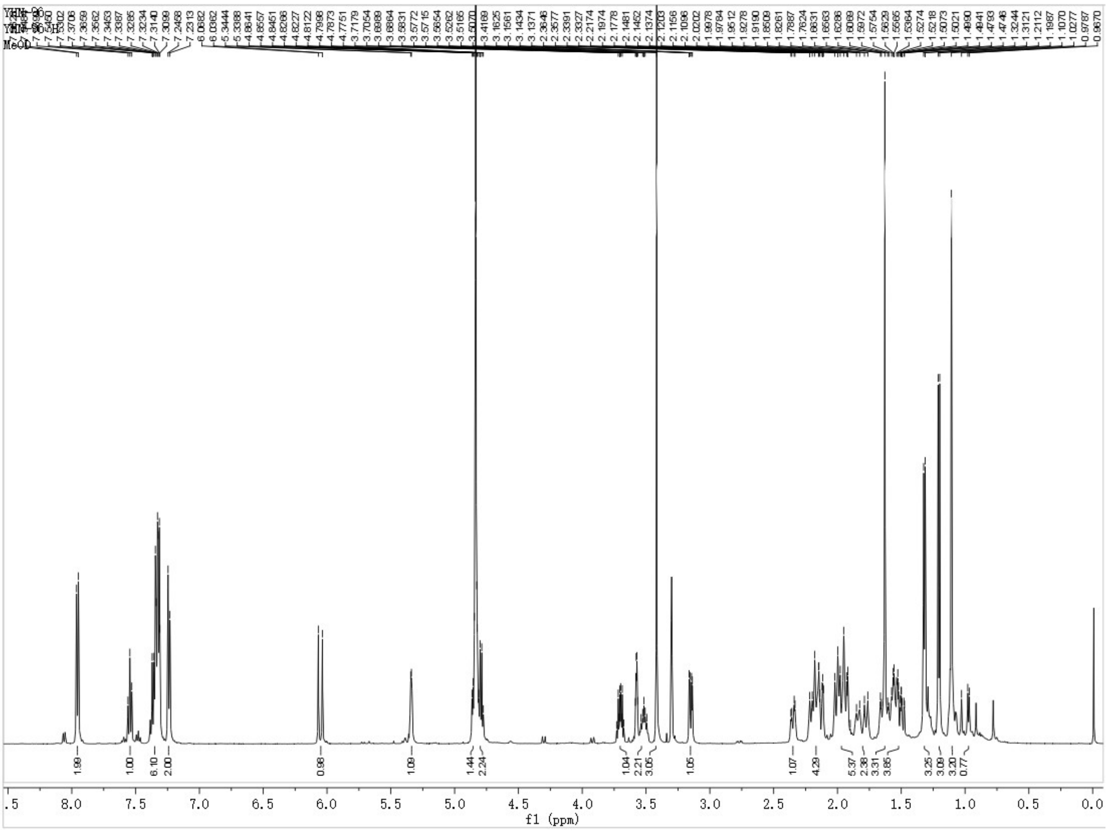

Figure S15. ^{1}H NMR spectrum (500 MHz) of **3** in CD_3OD

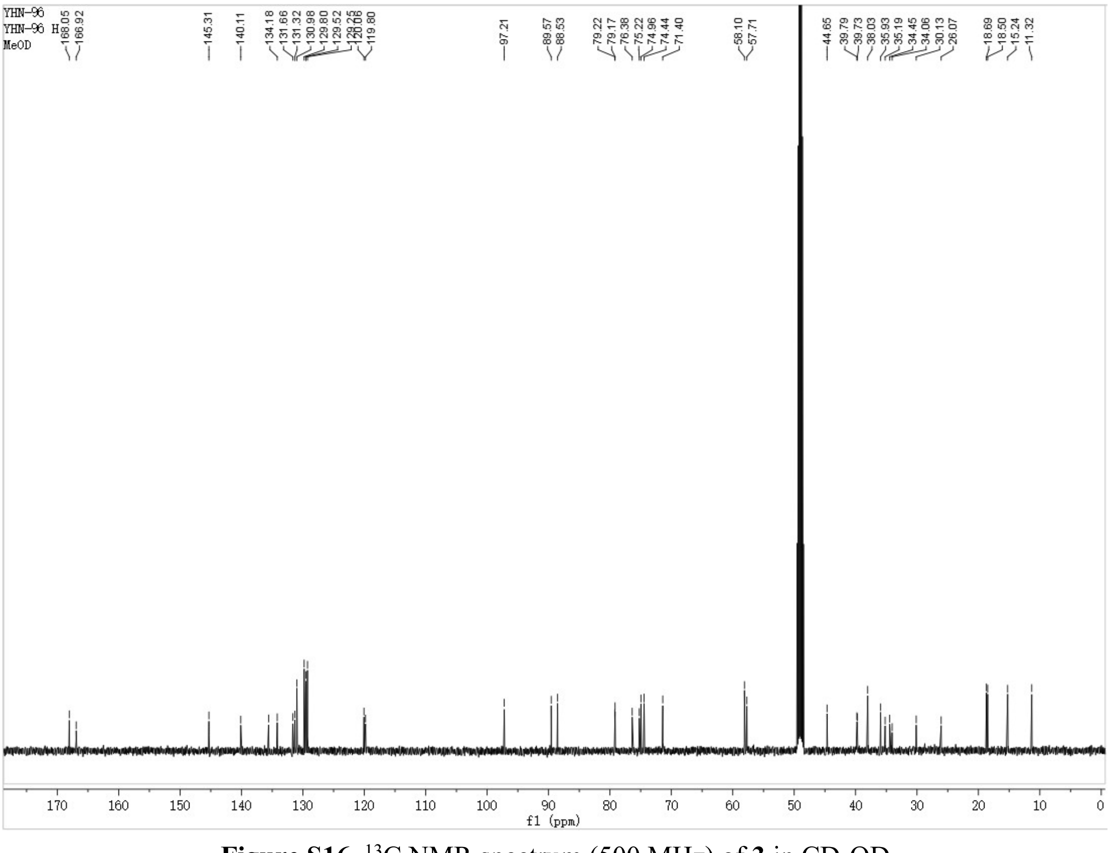

Figure S16. ^{13}C NMR spectrum (500 MHz) of **3** in CD_3OD

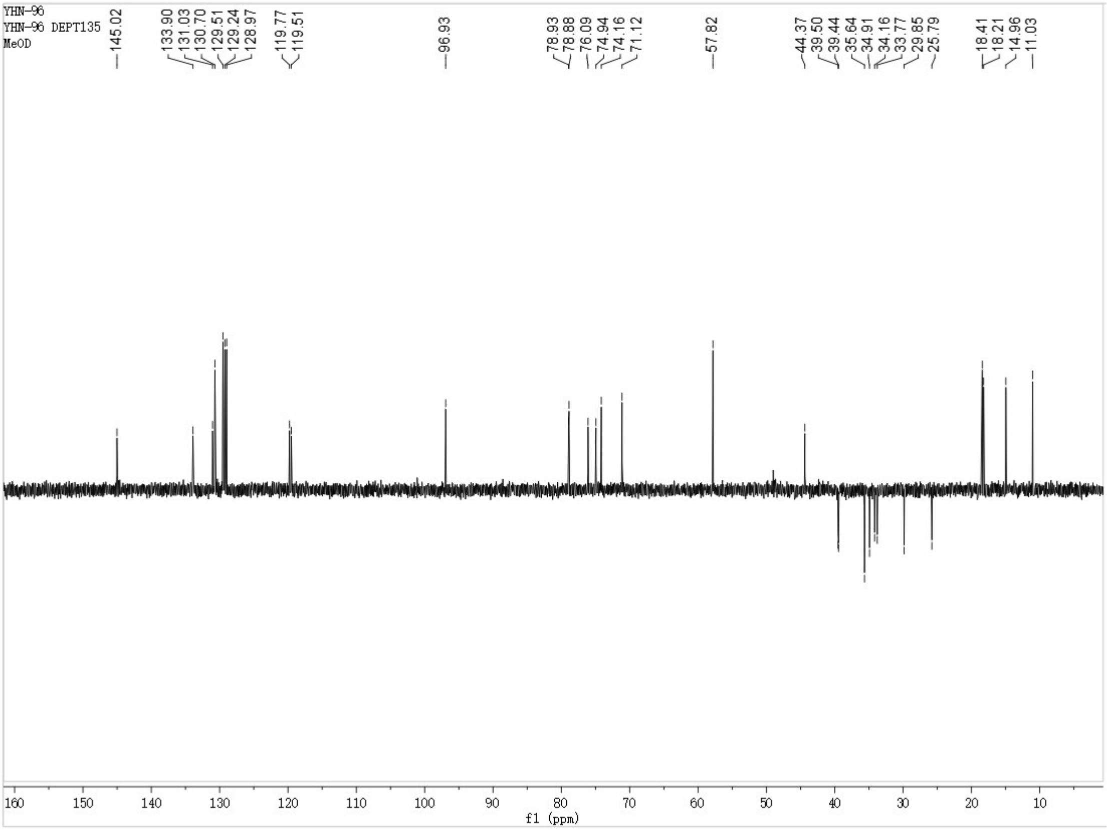

Figure S17. DEPT spectrum of **3** in CD_3OD

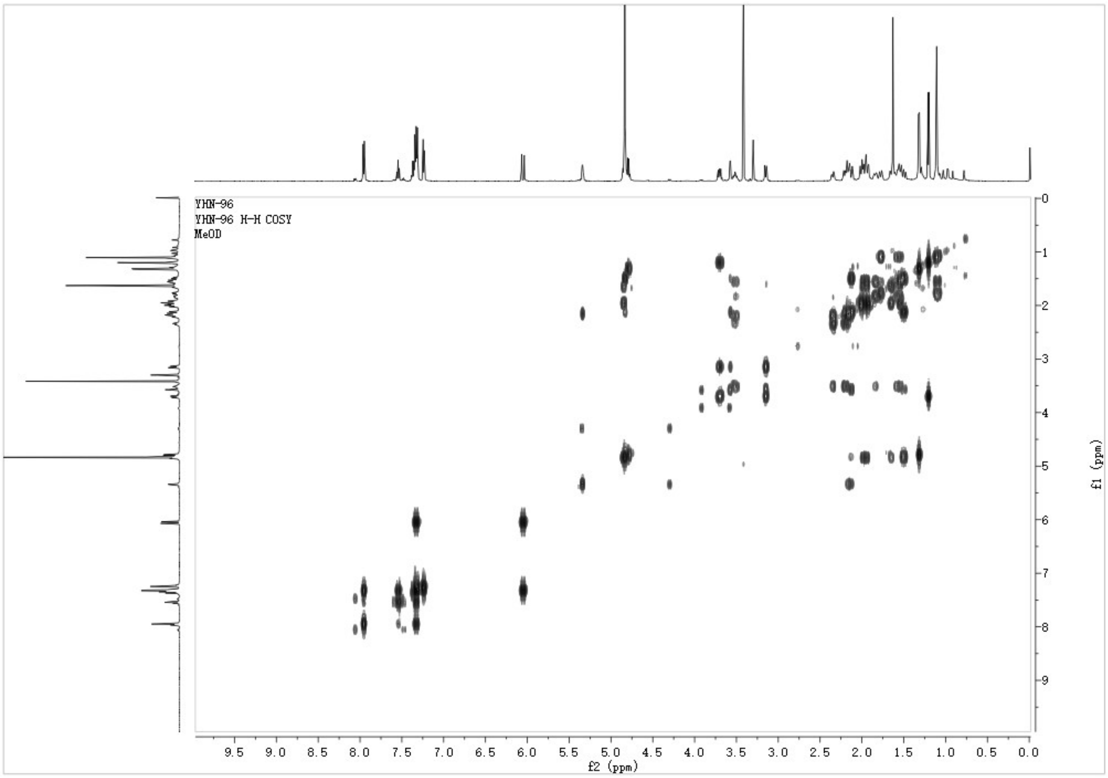

Figure S18. ^{1}H-^{1}H COSY of **3** in CD_3OD

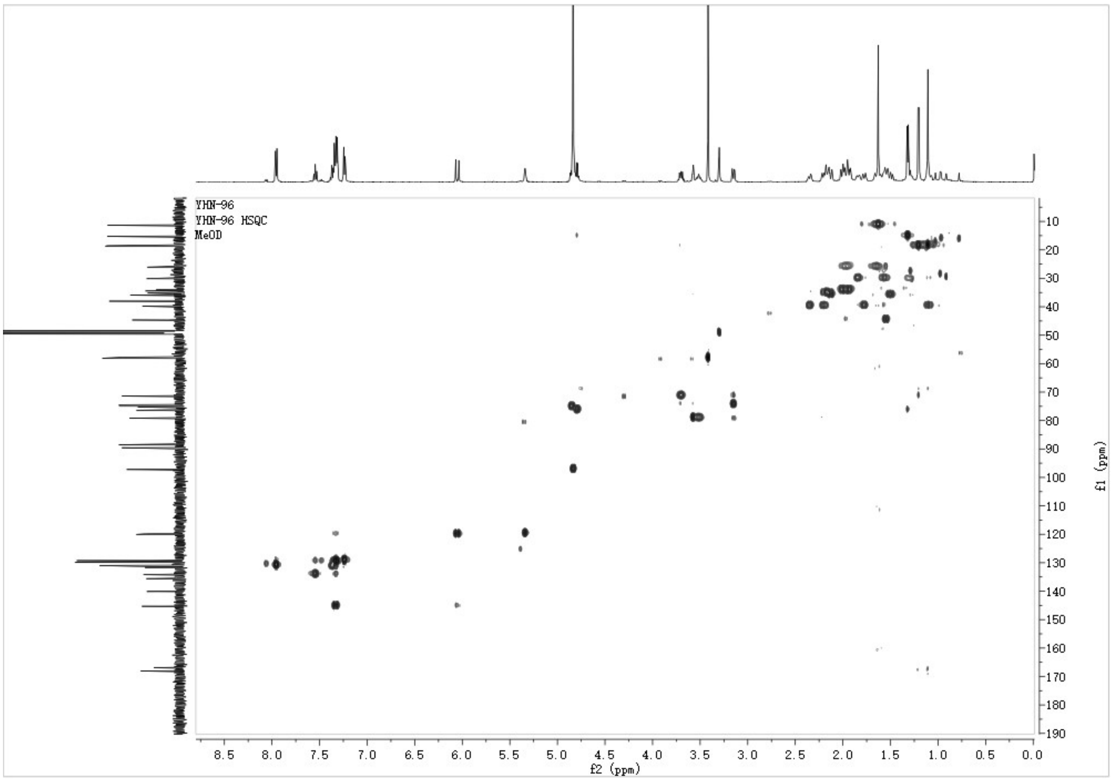

Figure S19. HSQC of **3** in CD_3OD

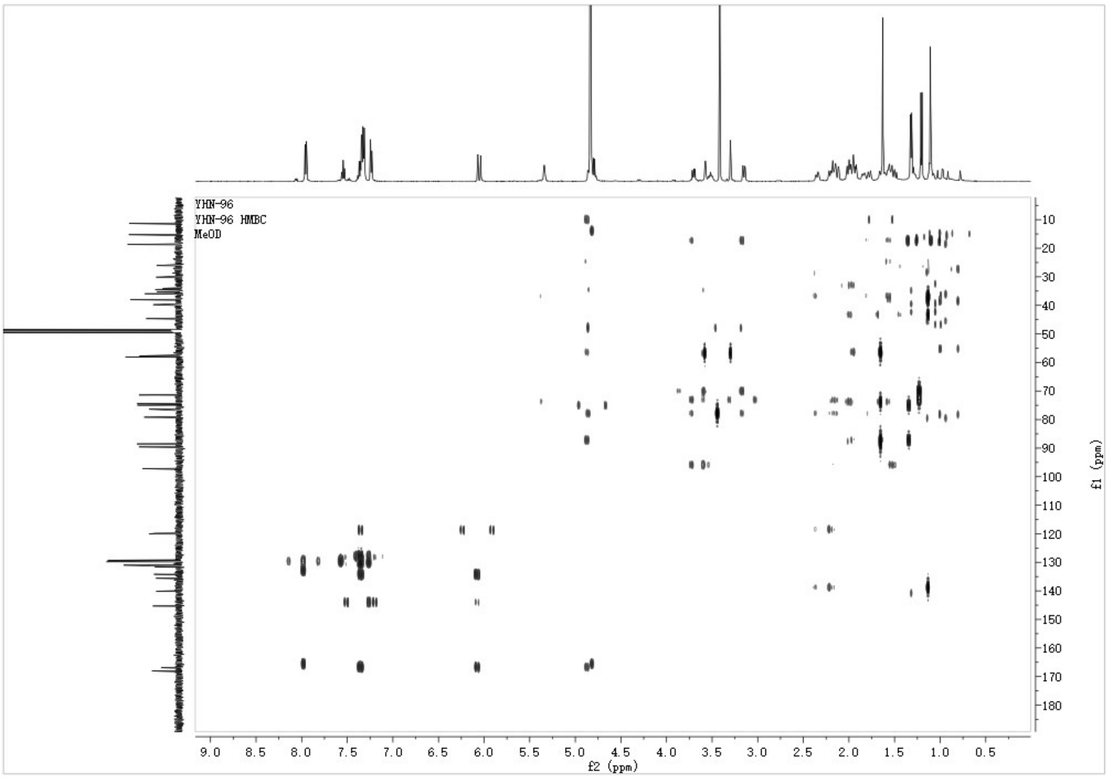

Figure S20. HMBC of **3** in CD_3OD

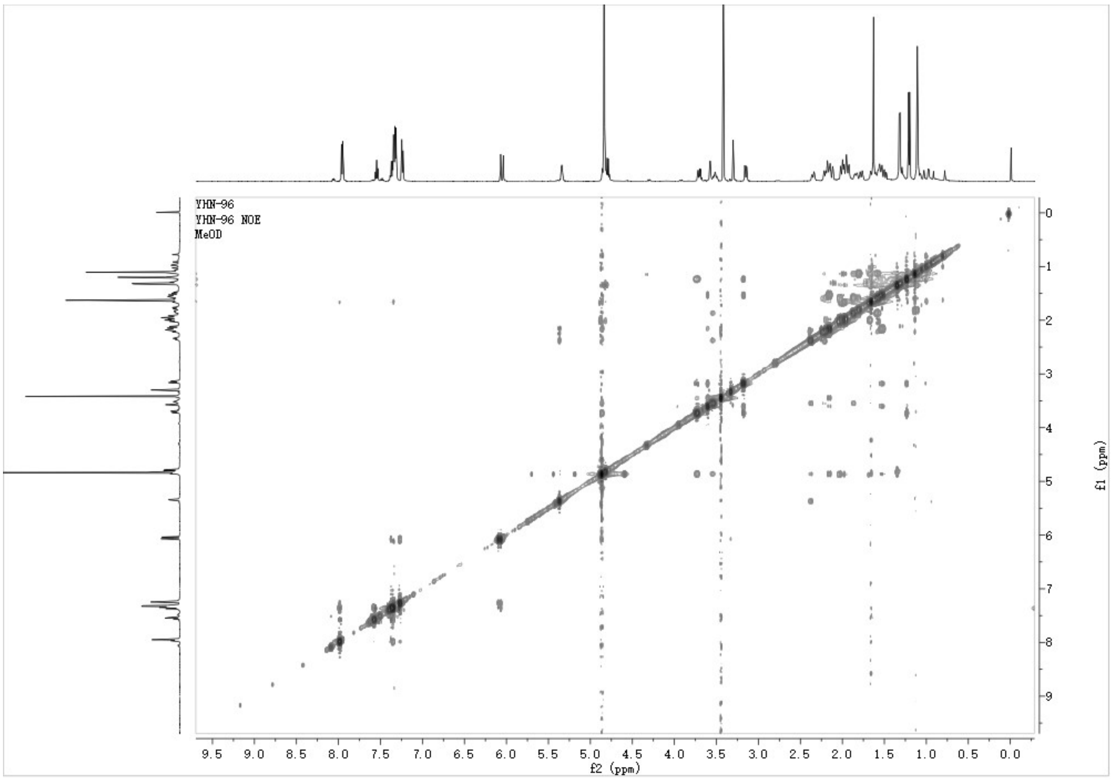

Figure S21. NOESY of **3** in CD_3OD

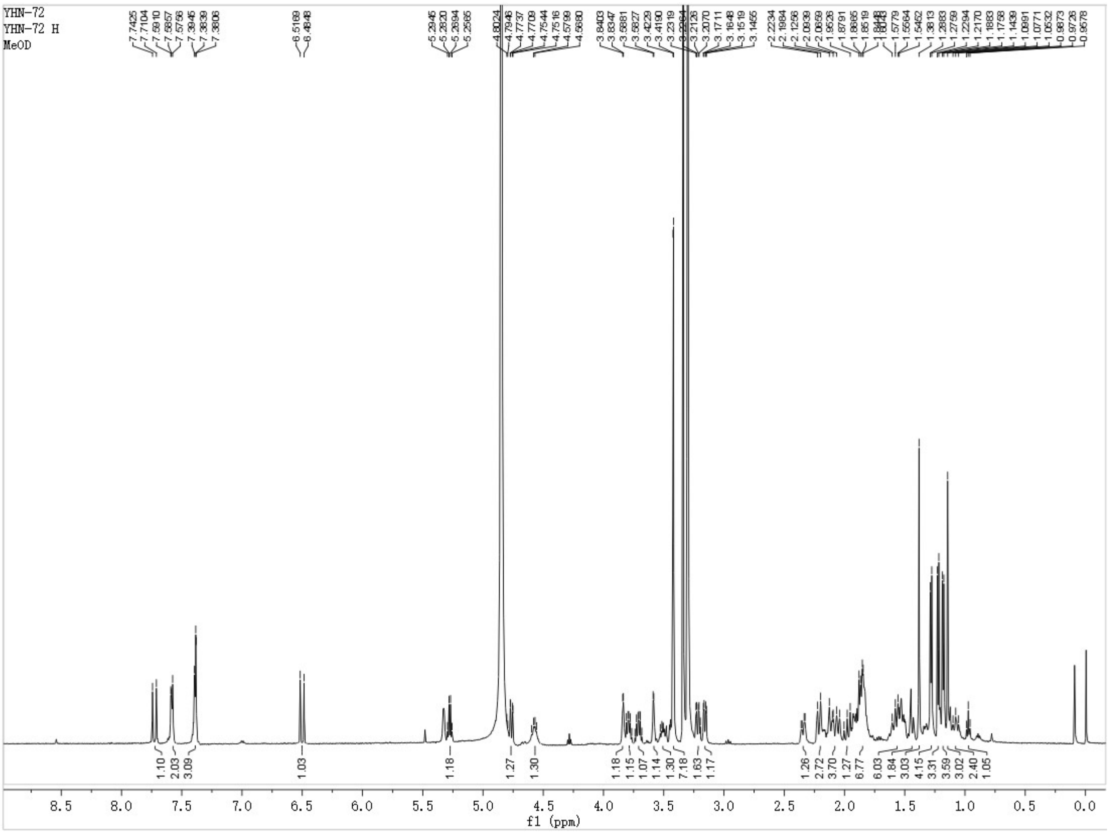

Figure S22. ^1H NMR spectrum (500 MHz) of **4** in CD_3OD

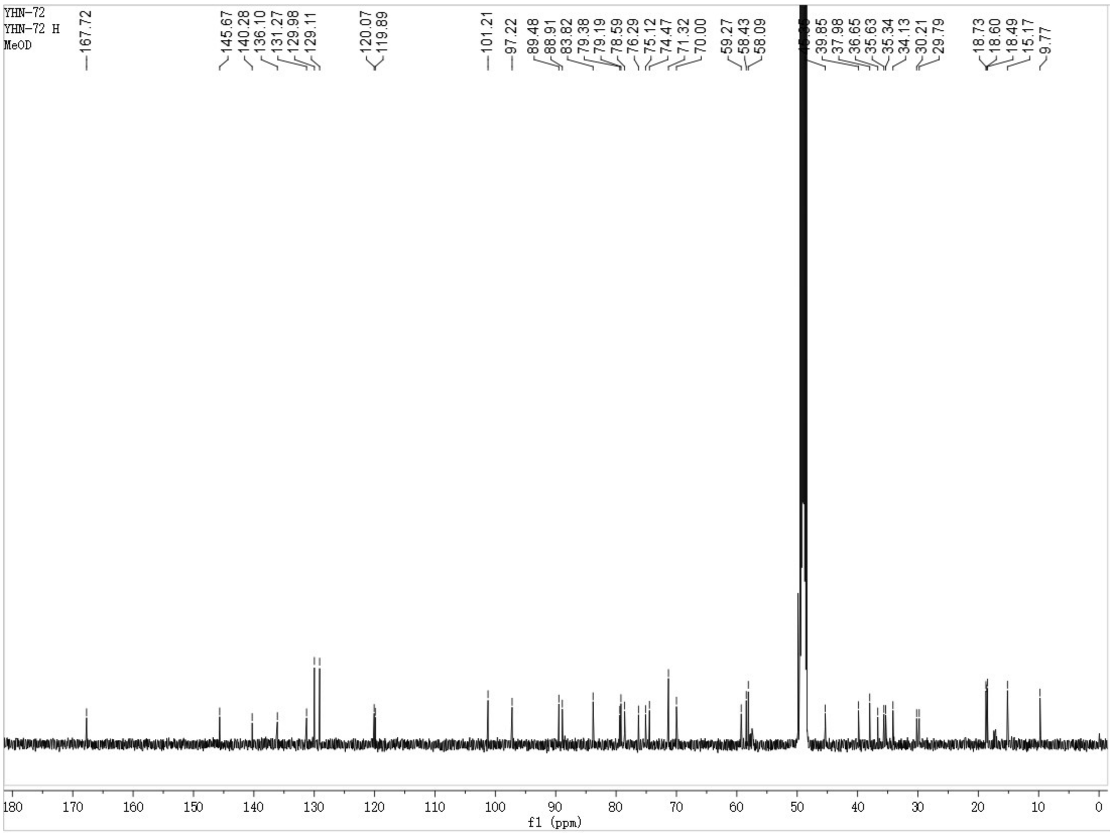

Figure S23. ^{13}C NMR spectrum (125 MHz) of **4** in CD_3OD

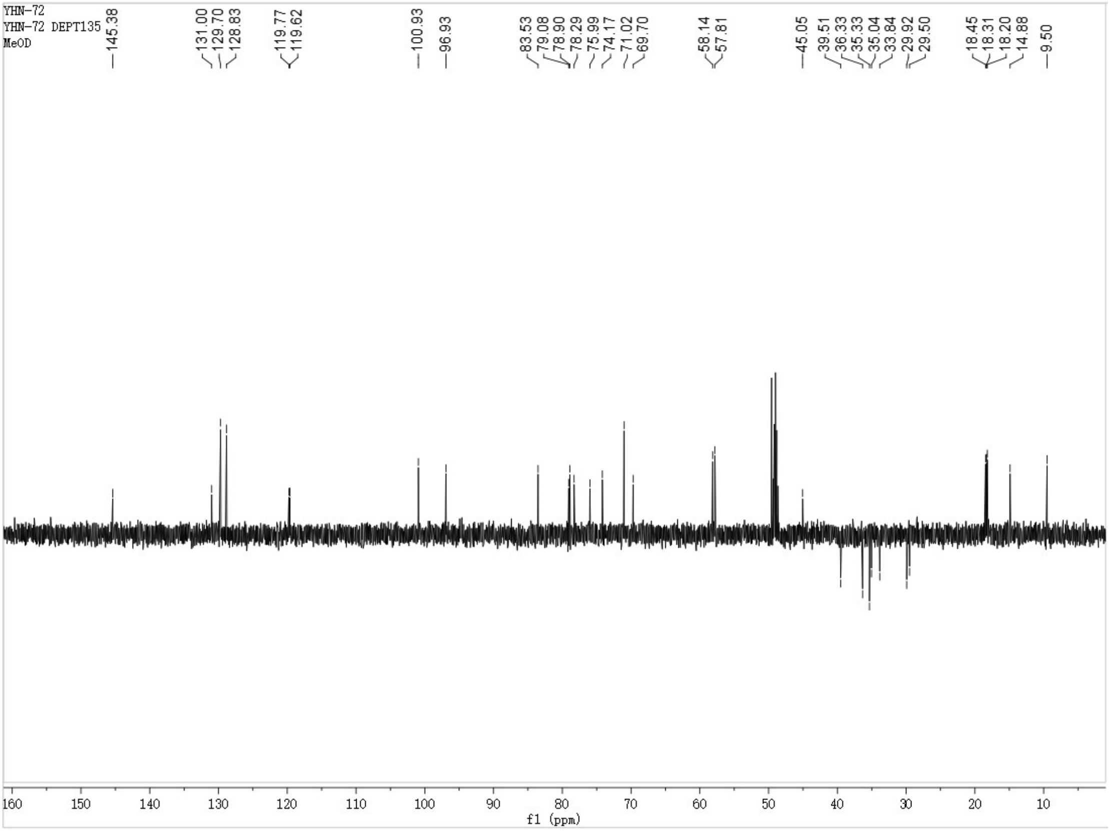

Figure S24. DEPT spectrum of **4** in CD_3OD

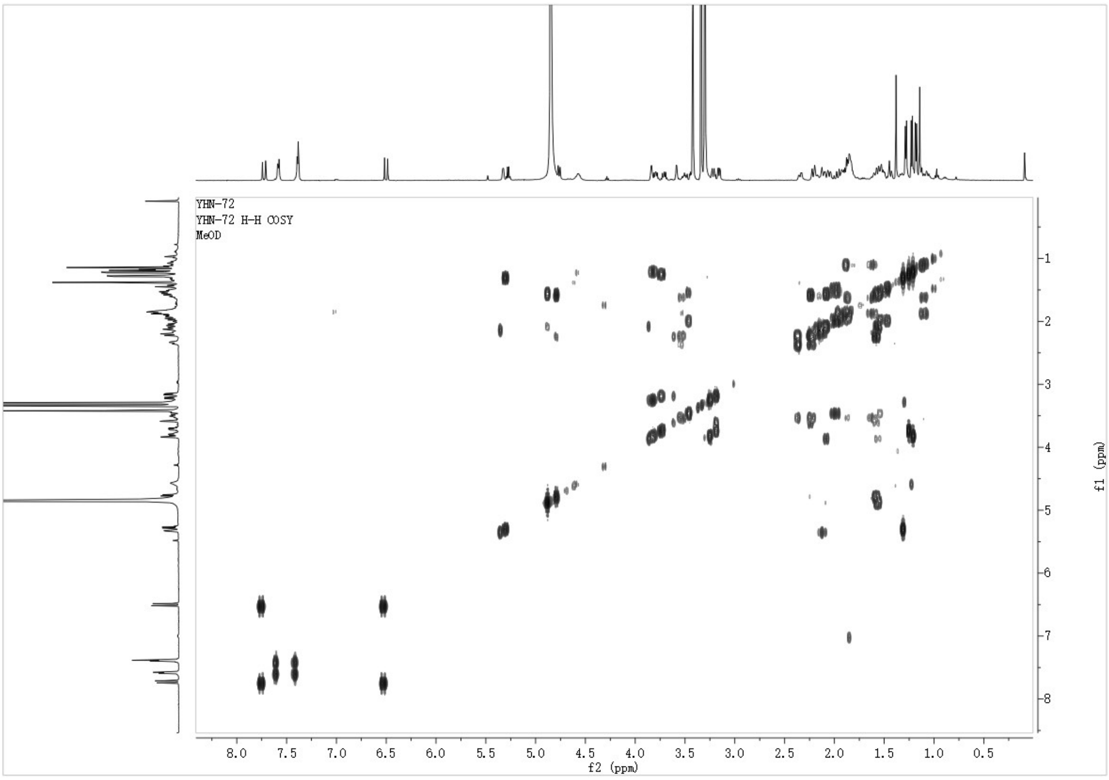

Figure S25. ^{1}H-^{1}H COSY of **4** in CD_3OD

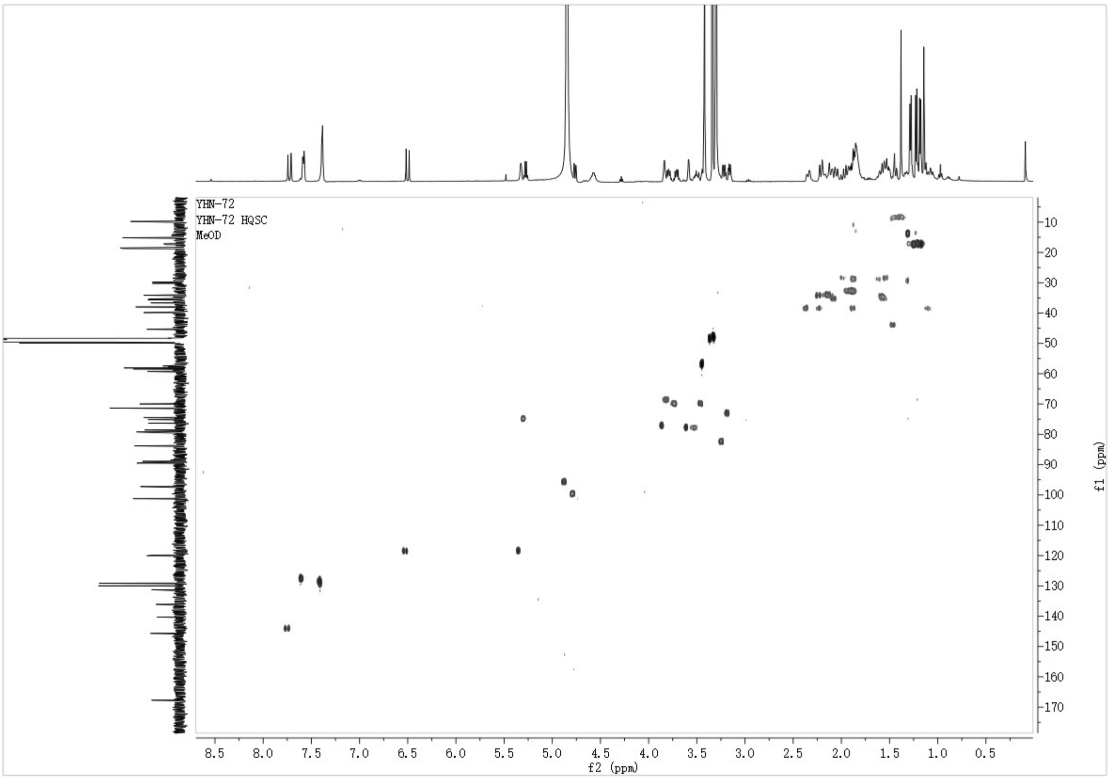

Figure S26. HSQC of **4** in CD_3OD

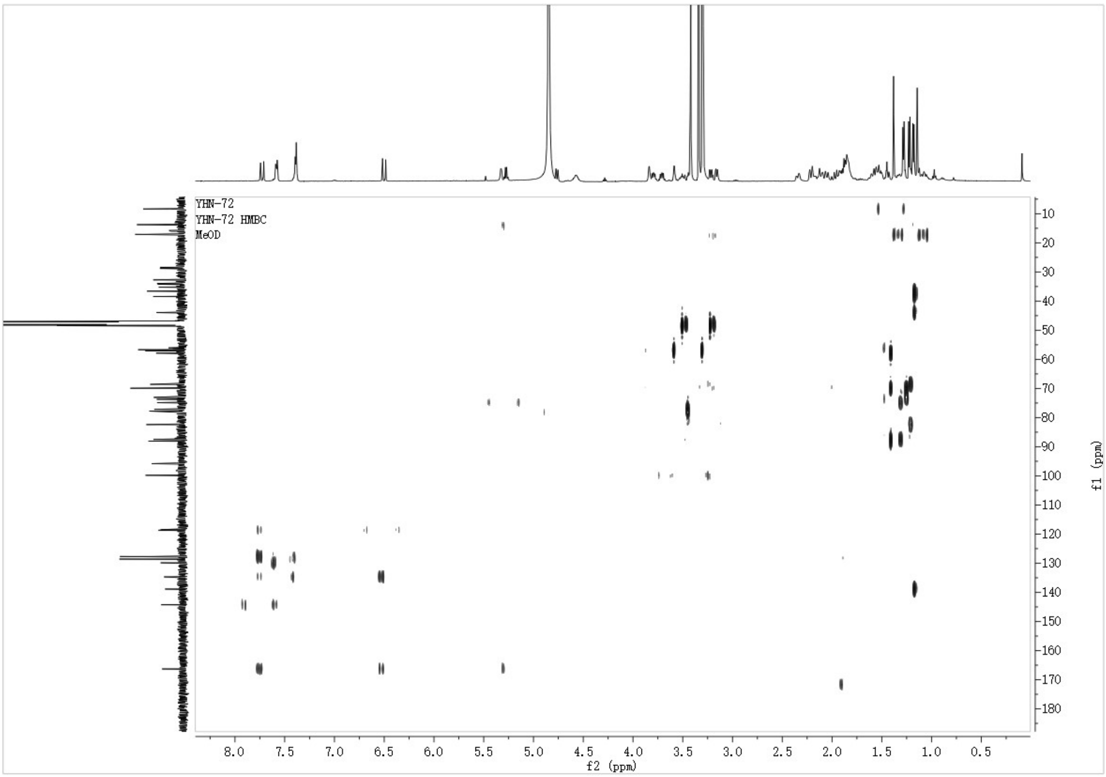

Figure S27. HMBC of **4** in CD_3OD

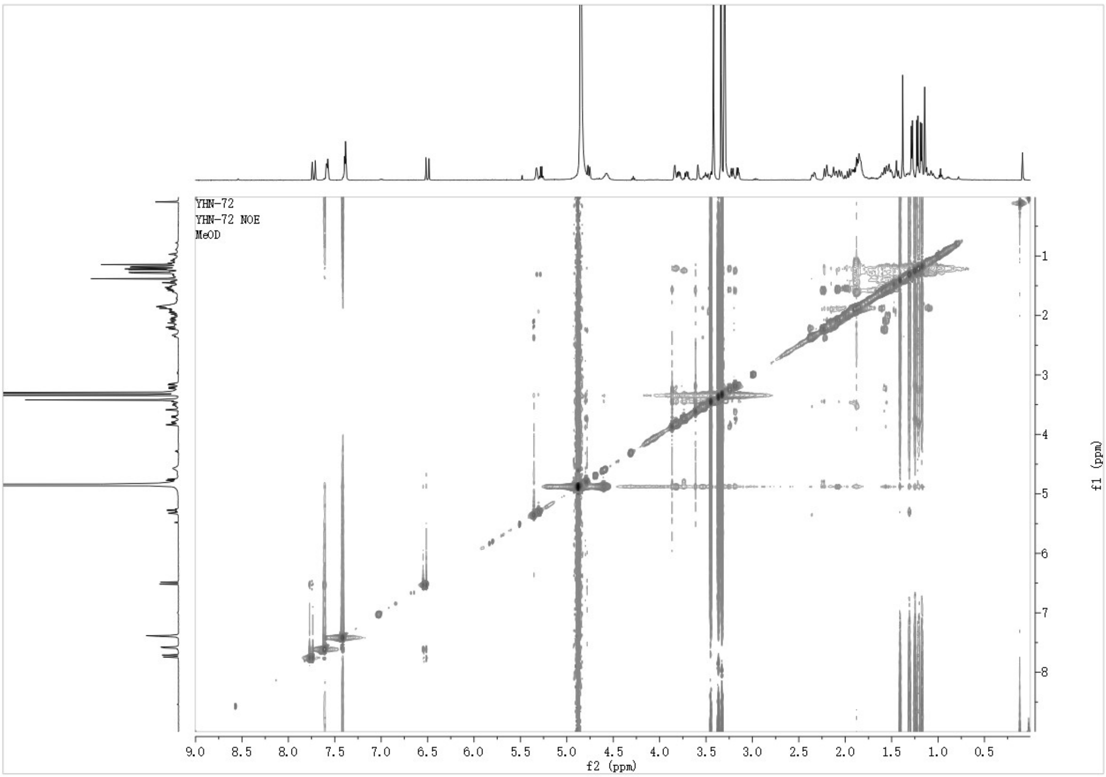

Figure S28. NOESY of **4** in CD_3OD

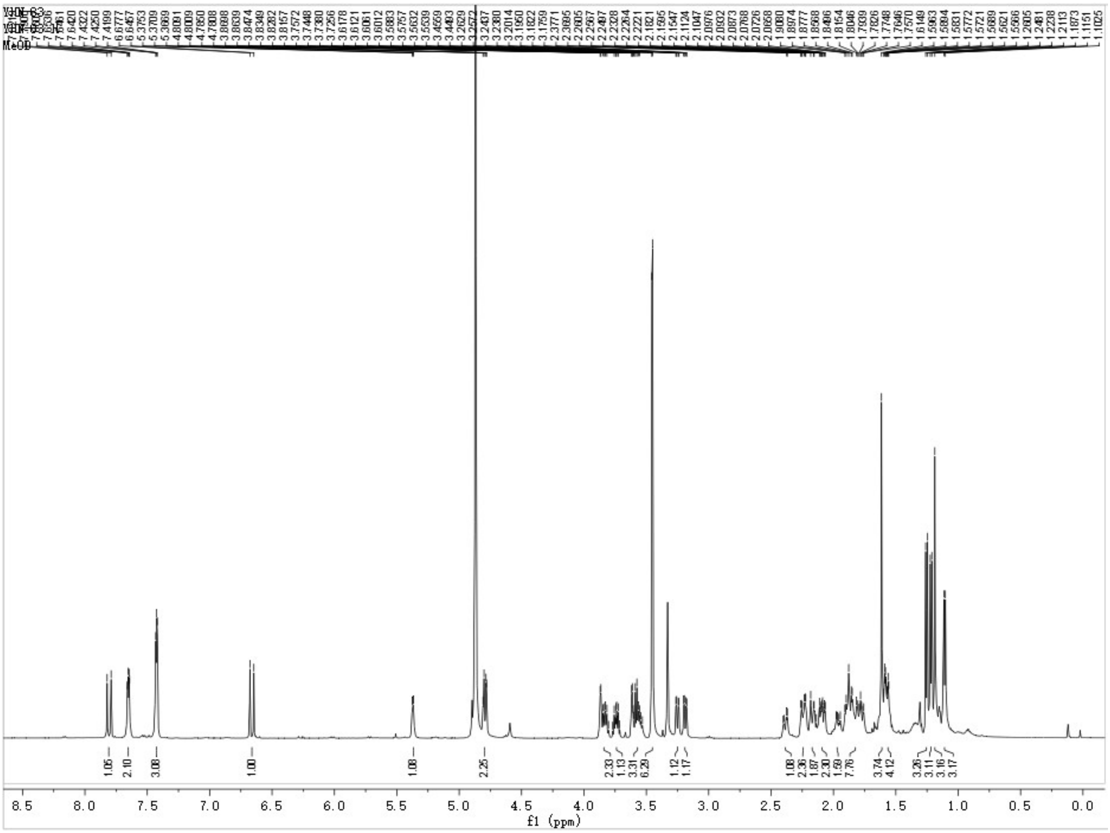

Figure S29. ^{1}H NMR spectrum (500 MHz) of **5** in CD_3OD

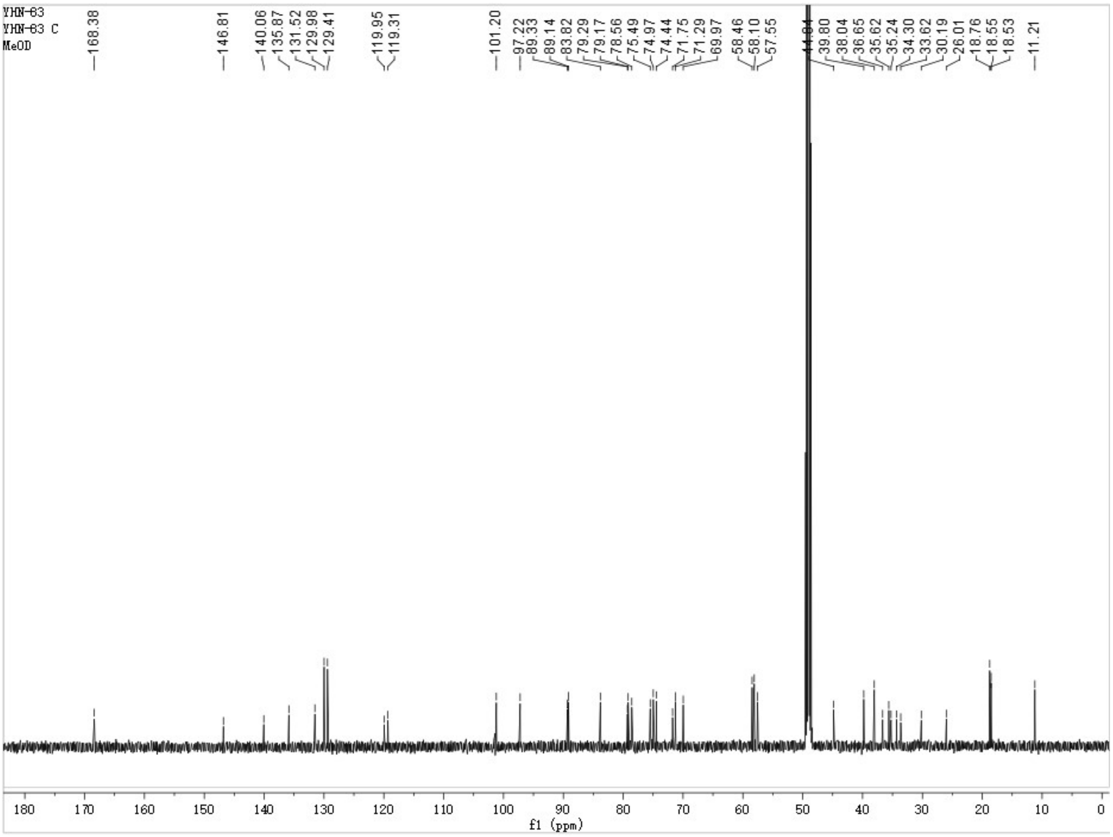

Figure S30. ^{13}C NMR spectrum (125 MHz) of **5** in CD_3OD

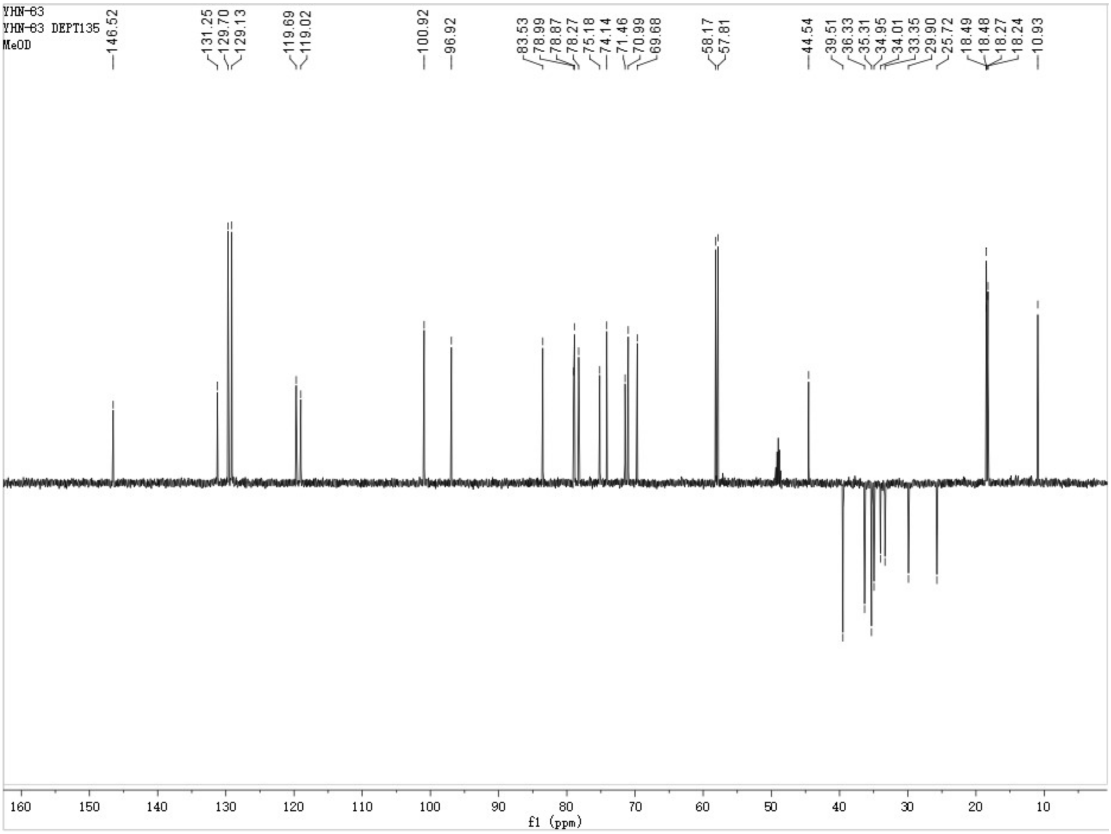

Figure S31. DEPT spectrum of **5** in CD_3OD

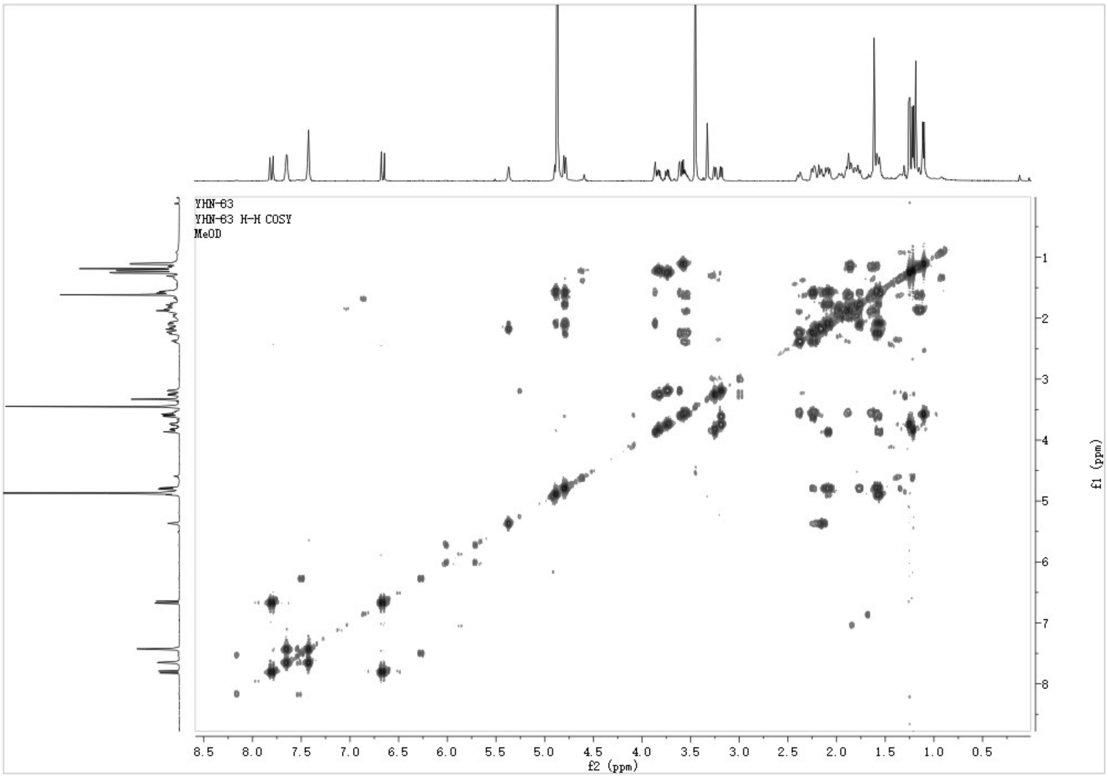

Figure S32. ^1H-^1H COSY of **5** in CD_3OD

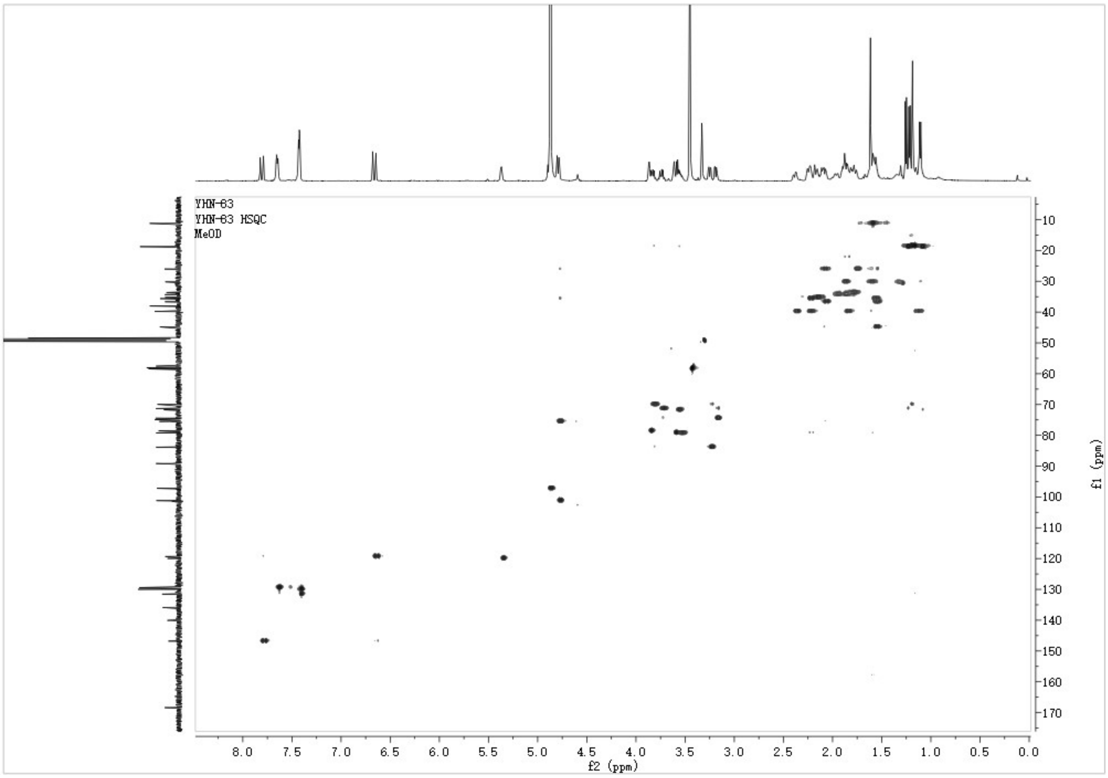

Figure S33. HSQC of **5** in CD_3OD

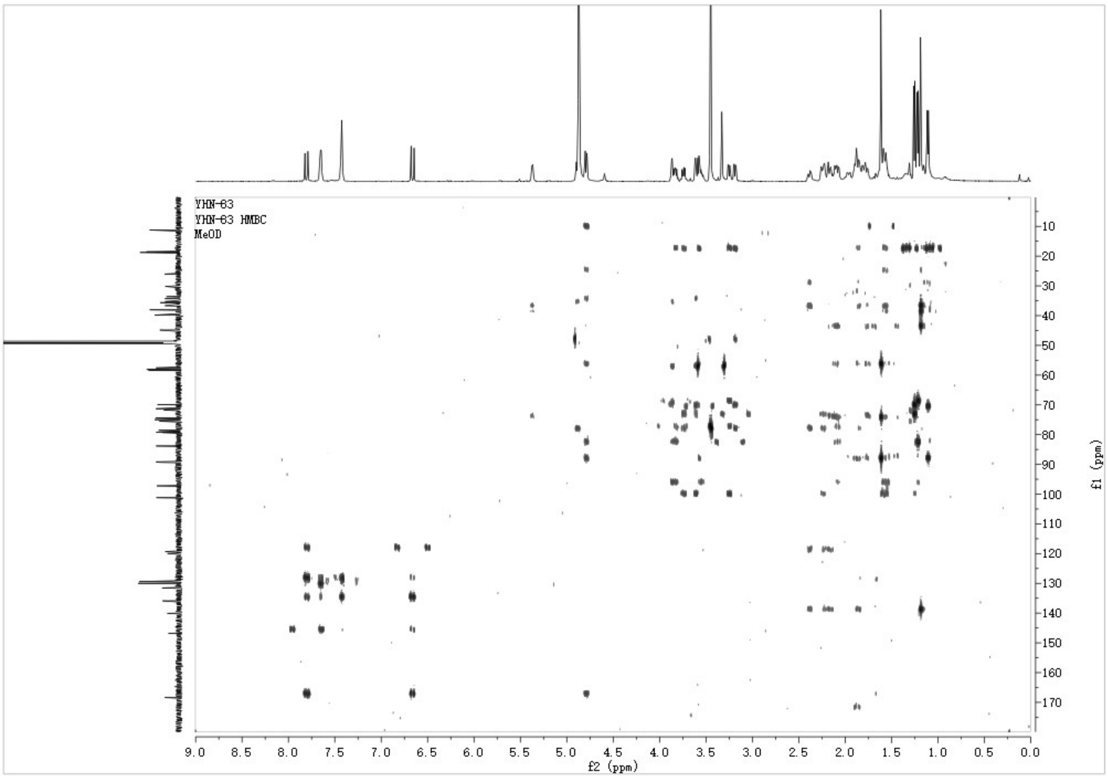

Figure S34. HMBC of **5** in CD_3OD

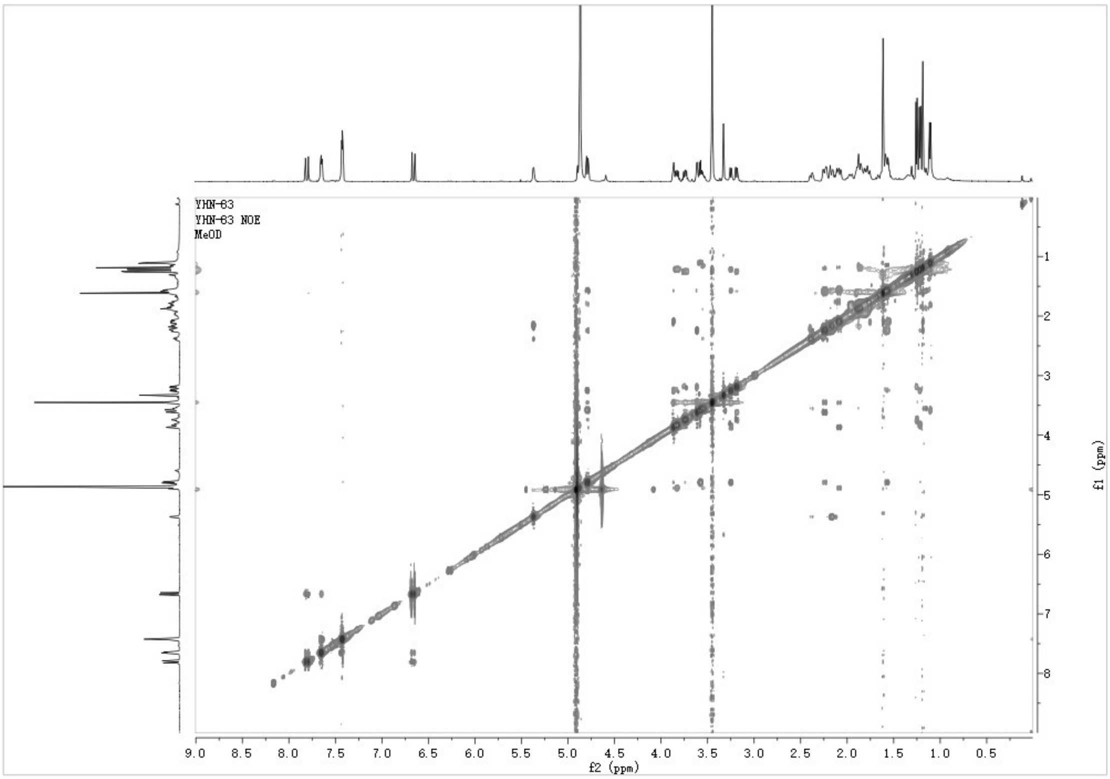

Figure S35. NOESY of **5** in CD_3OD

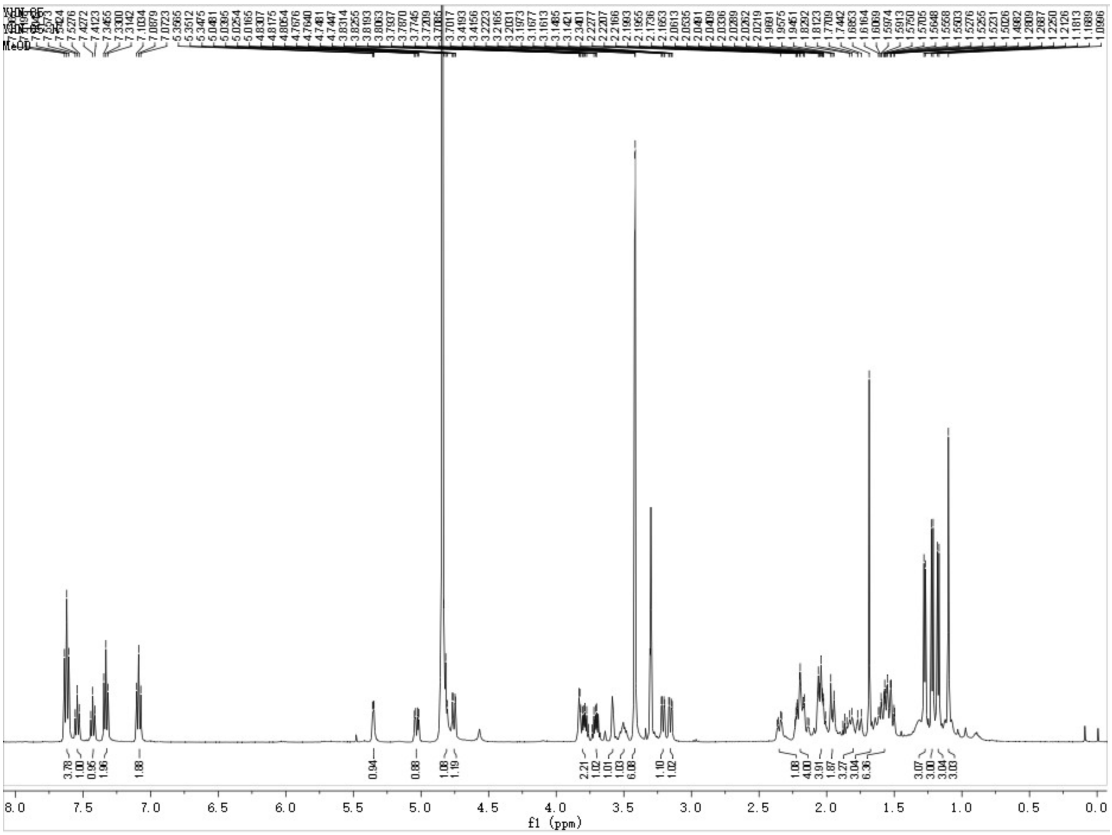

Figure S36. ^{1}H NMR spectrum (500 MHz) of **6** in CD_3OD

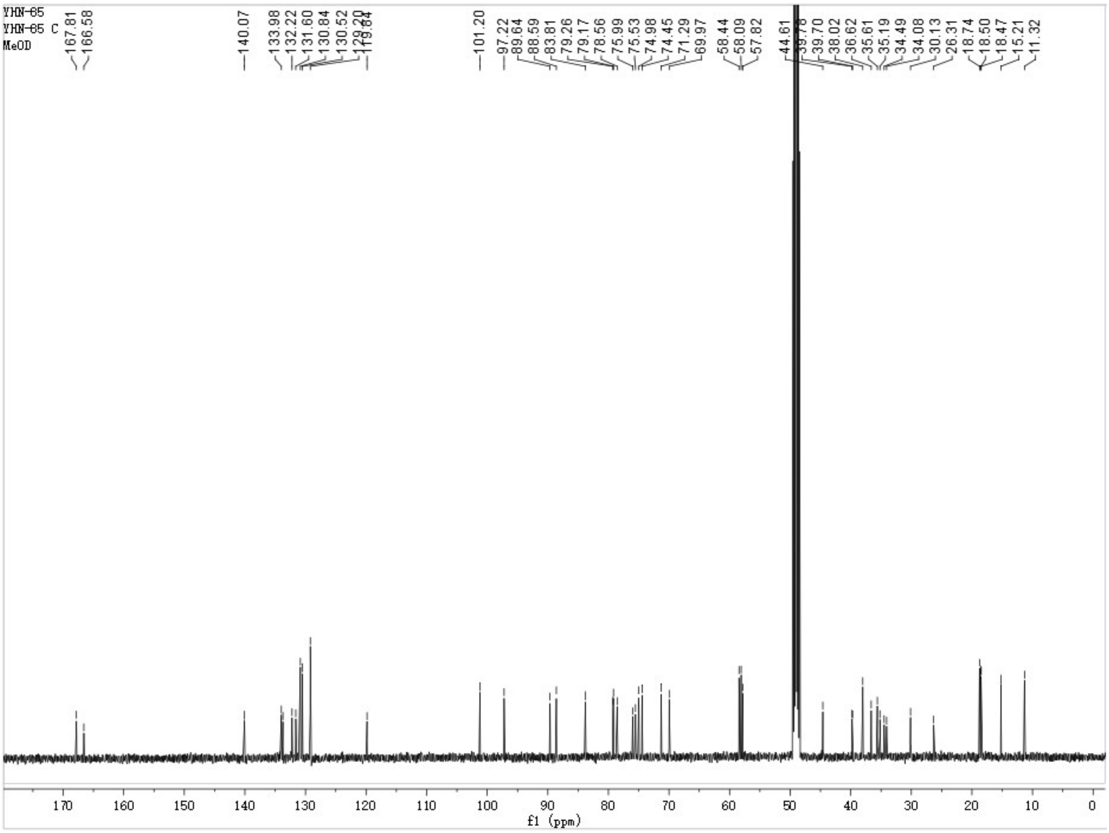

Figure S37. ^{13}C NMR spectrum (125 MHz) of **6** in CD_3OD

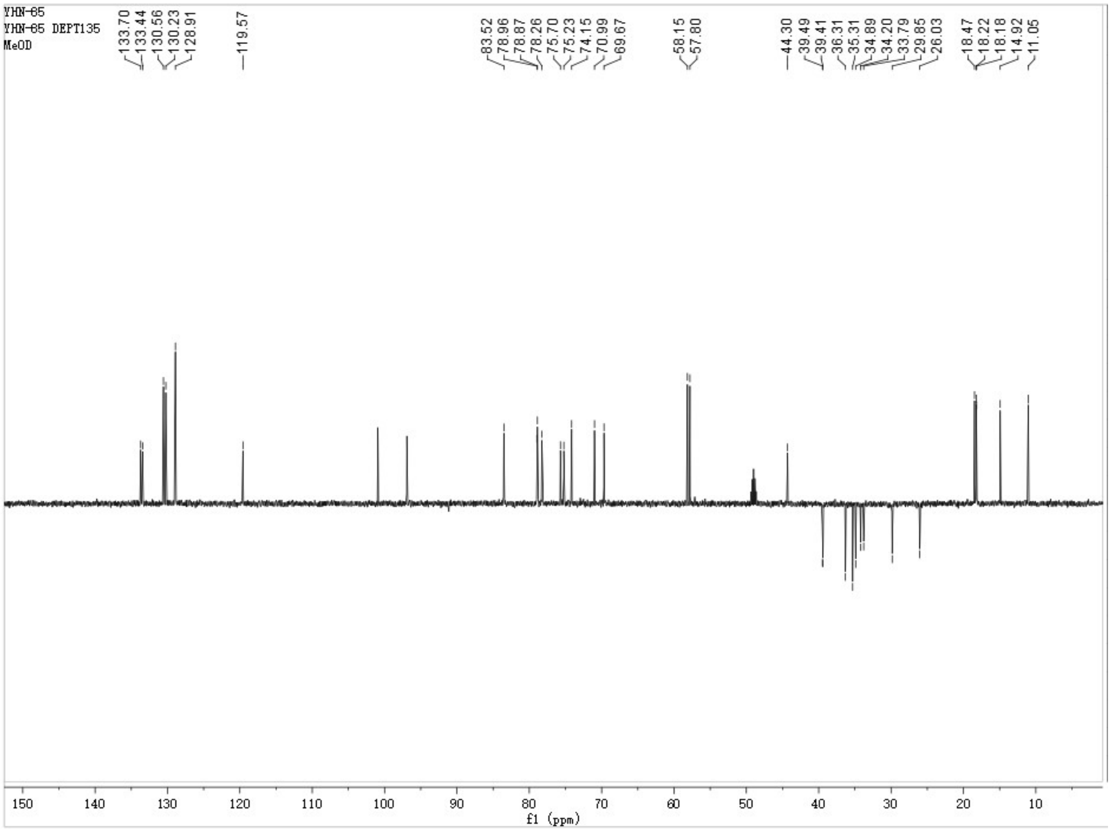

Figure S38. DEPT spectrum of **6** in CD_3OD

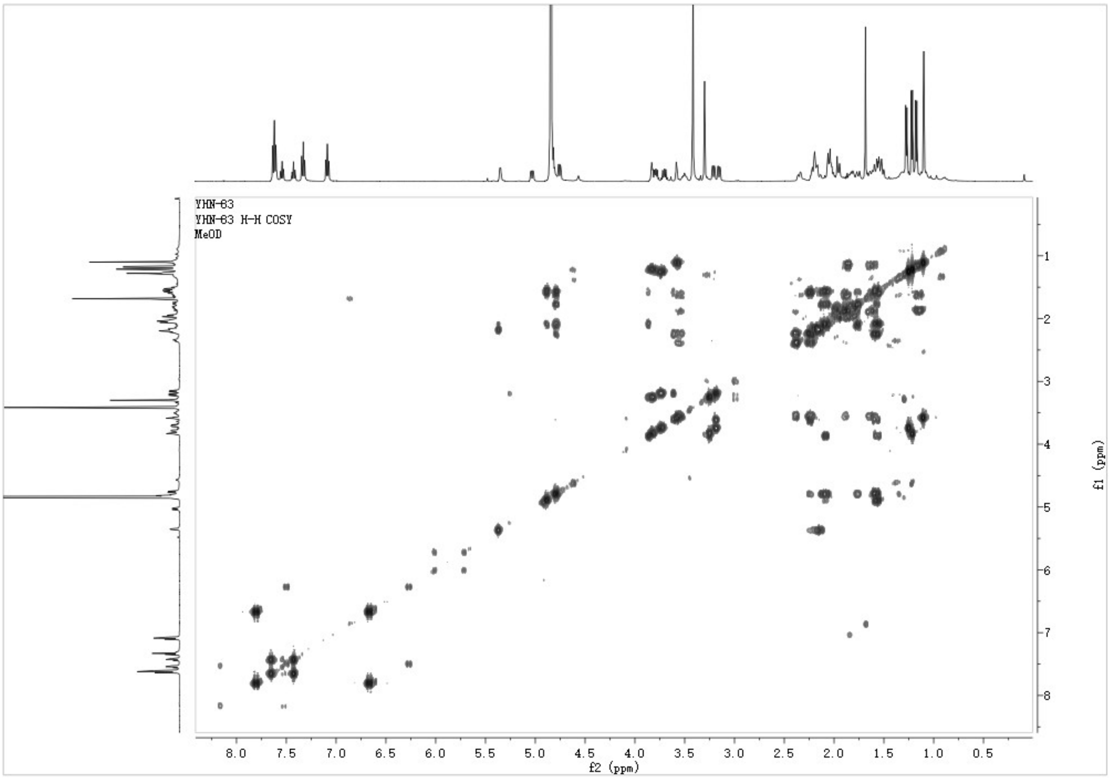

Figure S39. ^{1}H-^{1}H COSY of **6** in CD_3OD

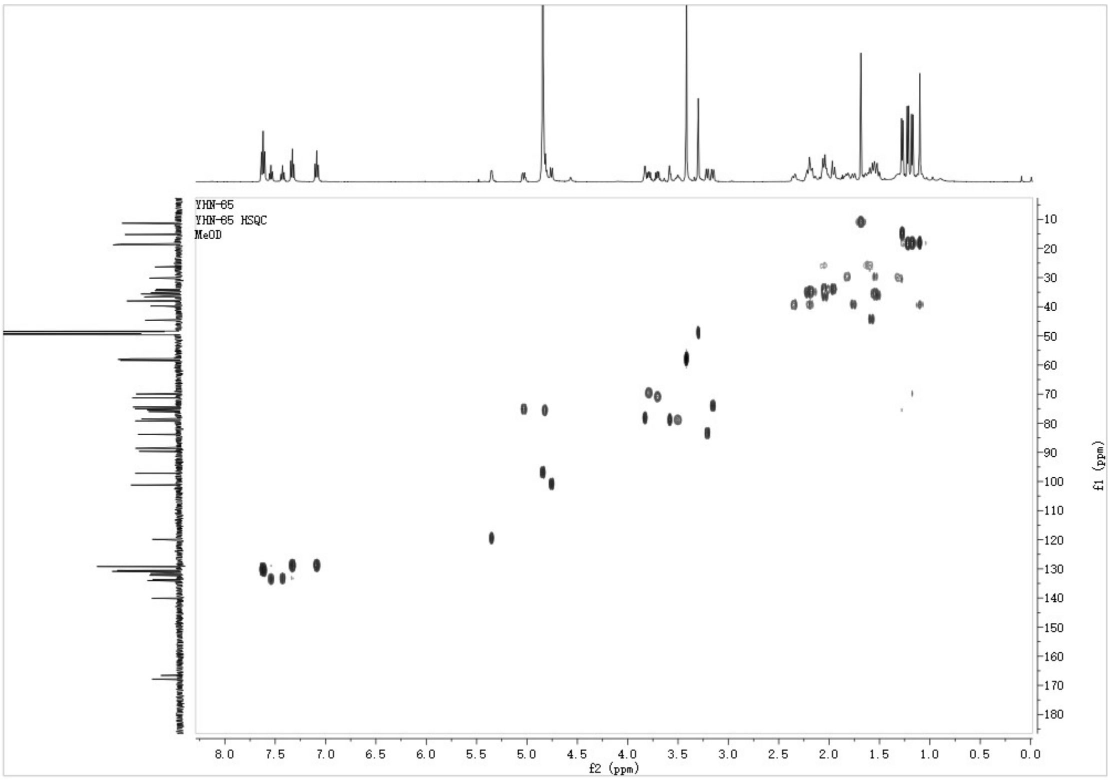

Figure S40. HSQC of **6** in CD_3OD

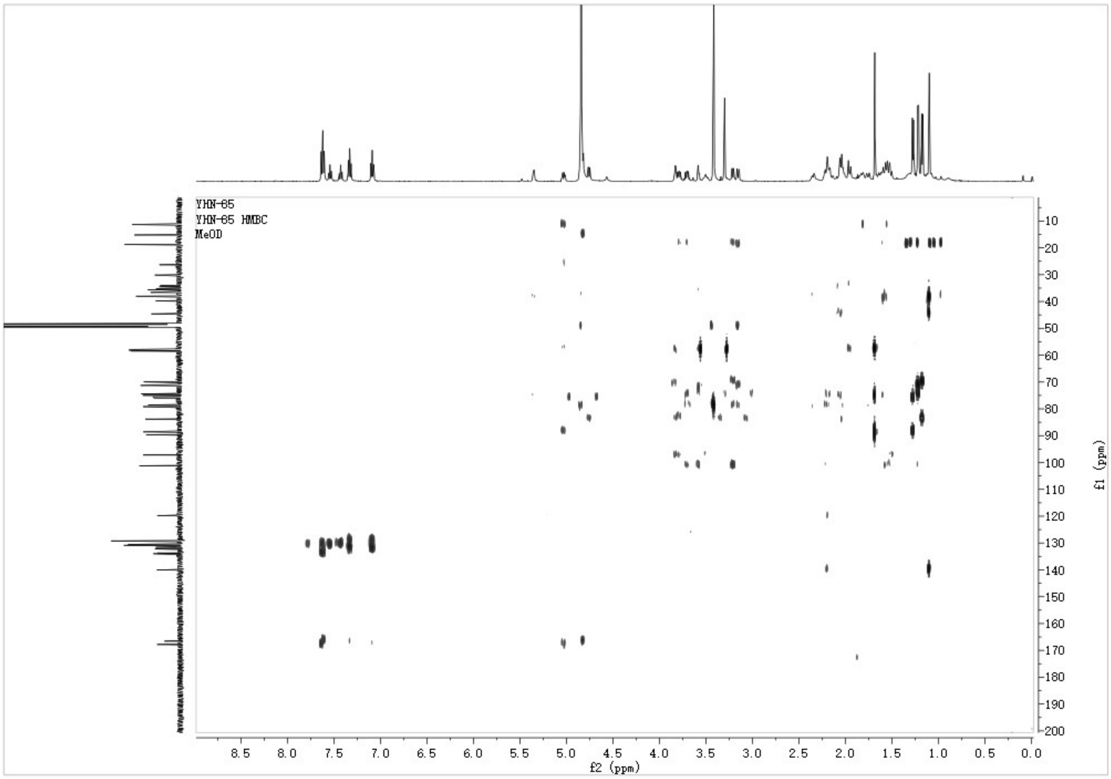

Figure S41. HMBC of **6** in CD_3OD

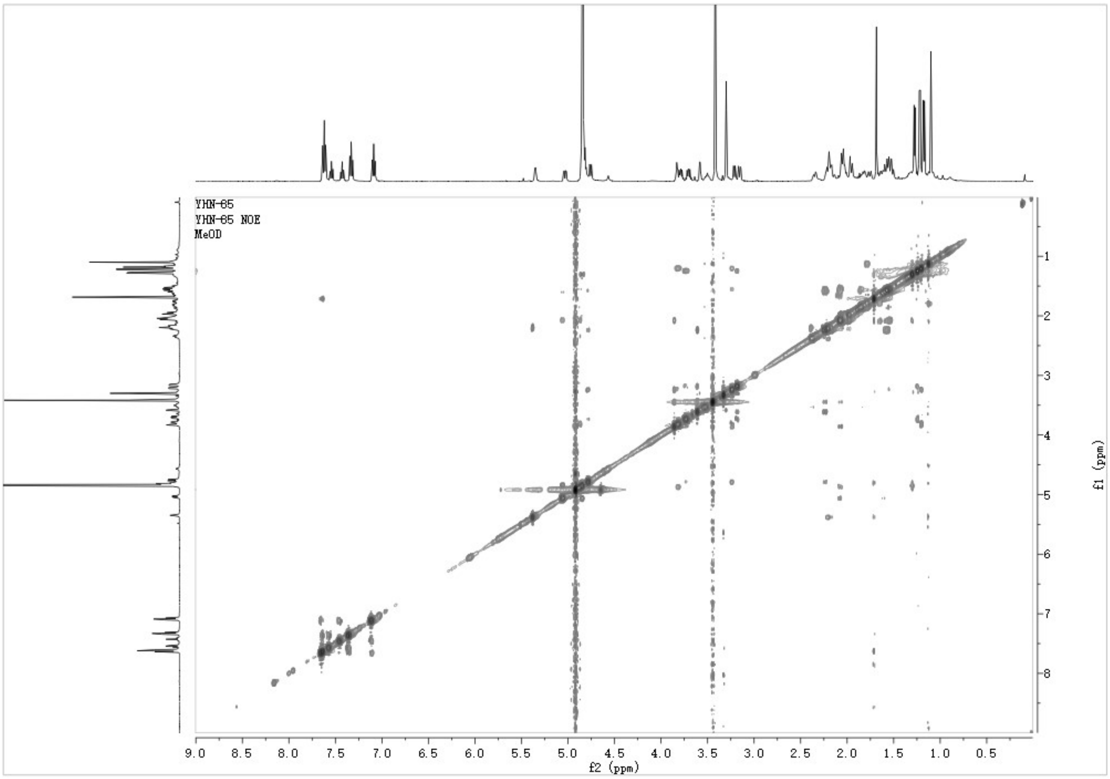

Figure S42. NOESY of **6** in CD_3OD

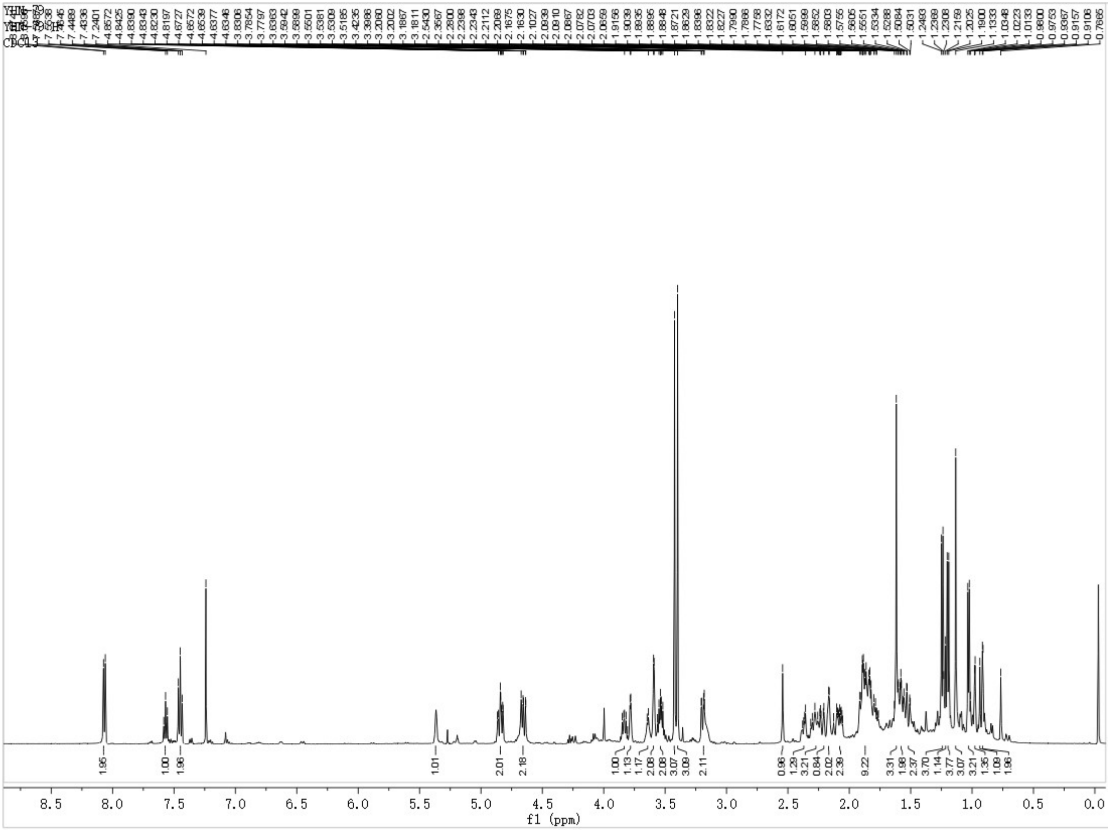

Figure S43. ^{1}H NMR spectrum (500 MHz) of **7** in $CDCl_3$

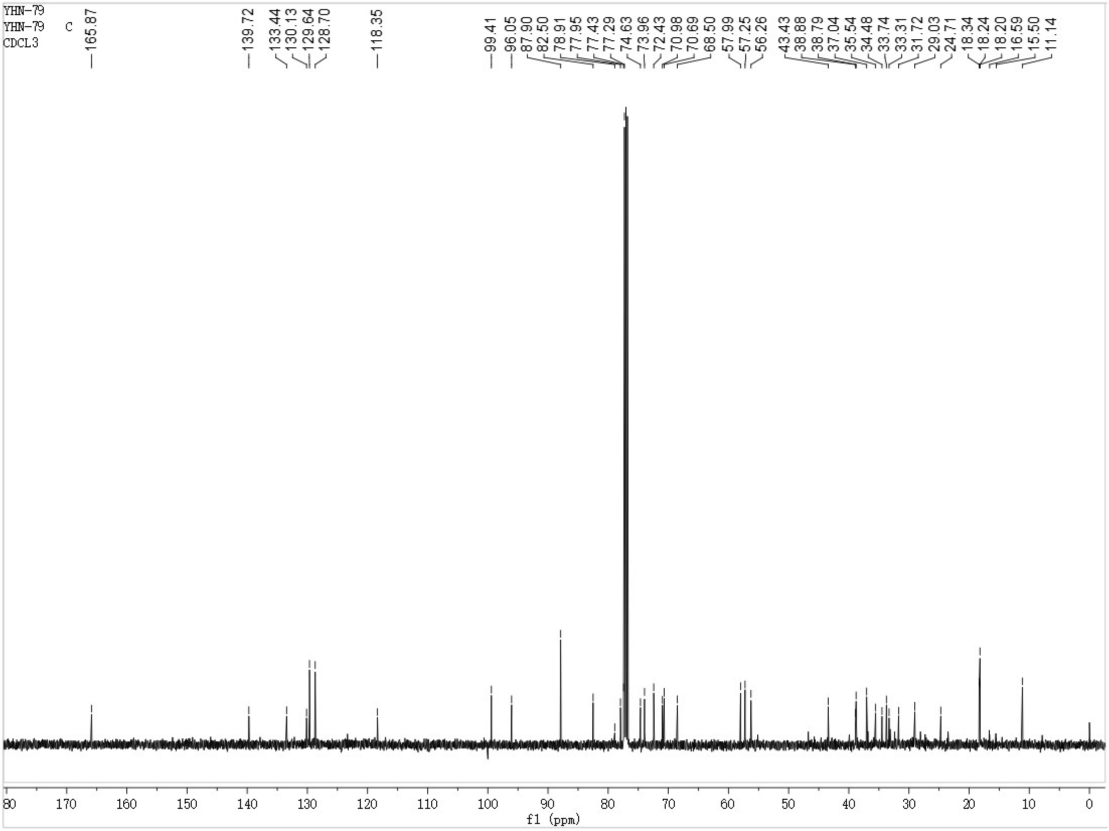

Figure S44. ^{13}C NMR spectrum (125 MHz) of **7** in$CDCl_3$

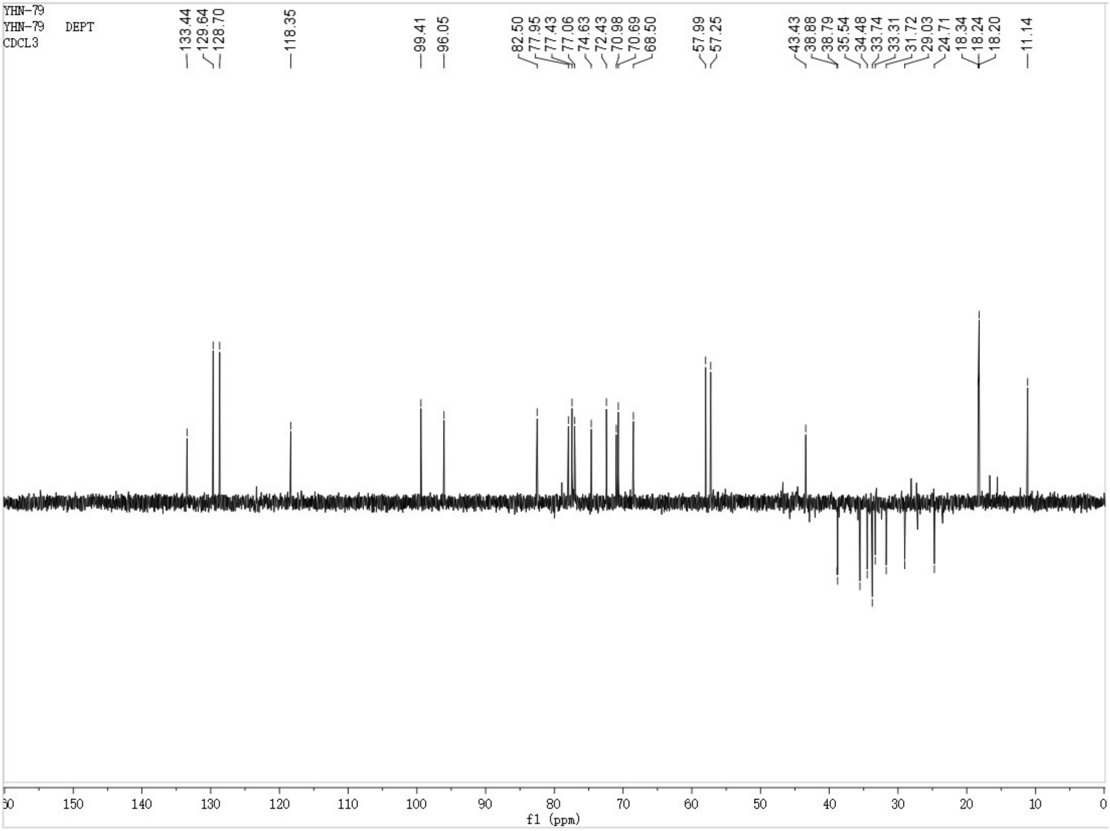

Figure S45. DEPT spectrum of **7** in $CDCl_3$

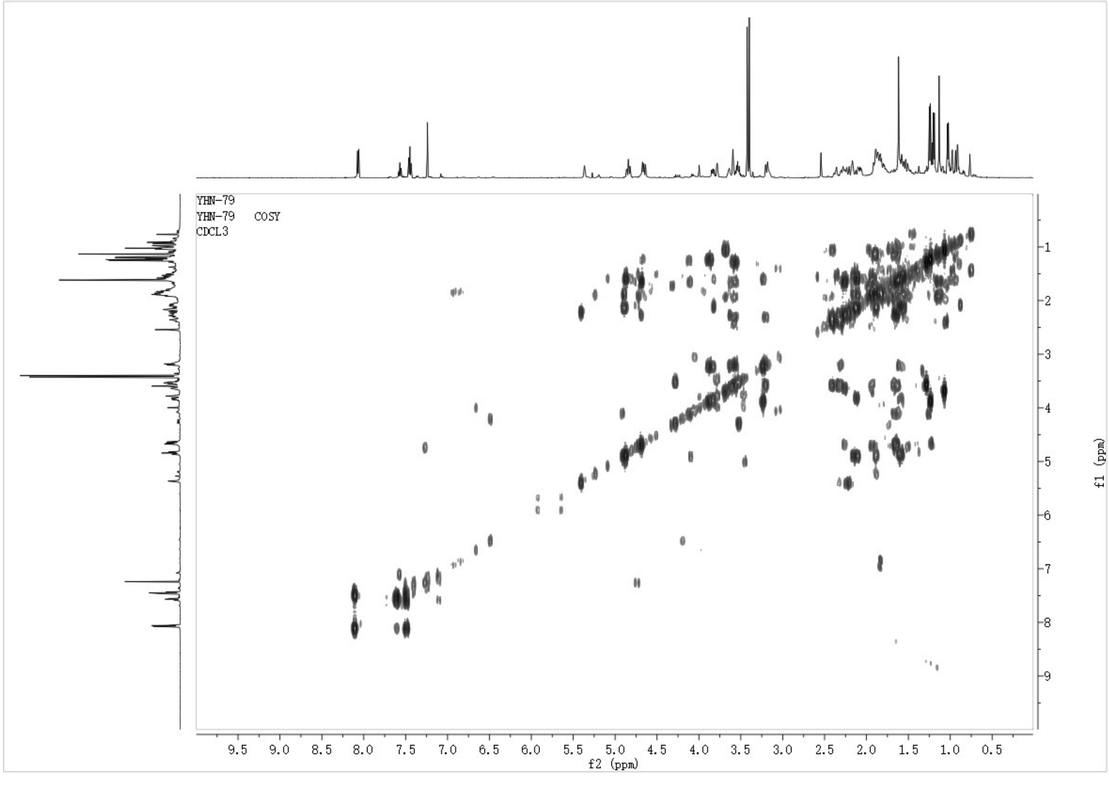

Figure S46. ^1H-^1H COSY of **7** in $CDCl_3$

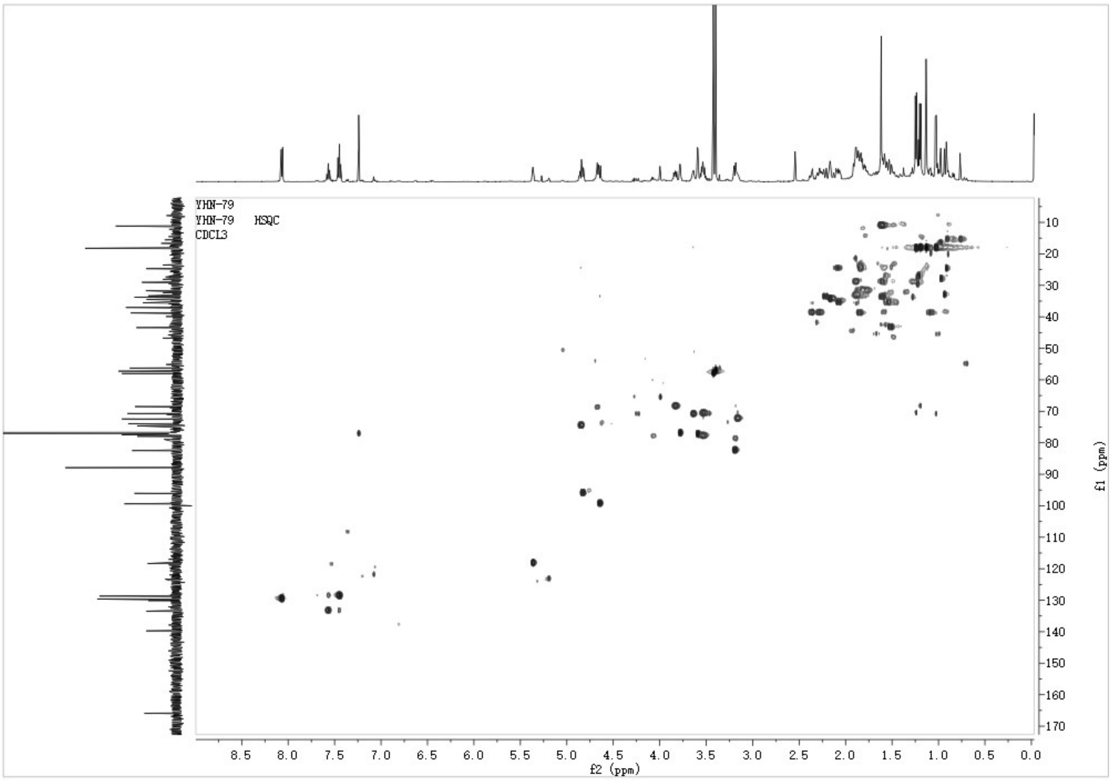

Figure S47. HSQC of **7** in $CDCl_3$

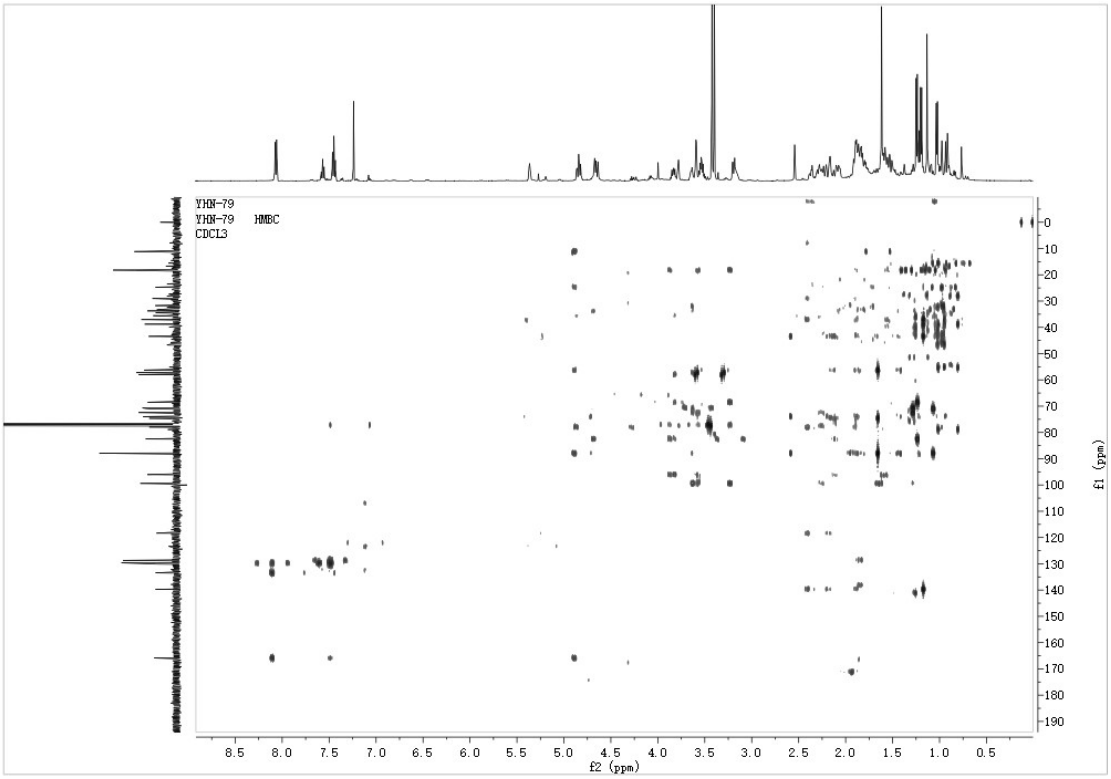

Figure S48. HMBC of **7** in $CDCl_3$

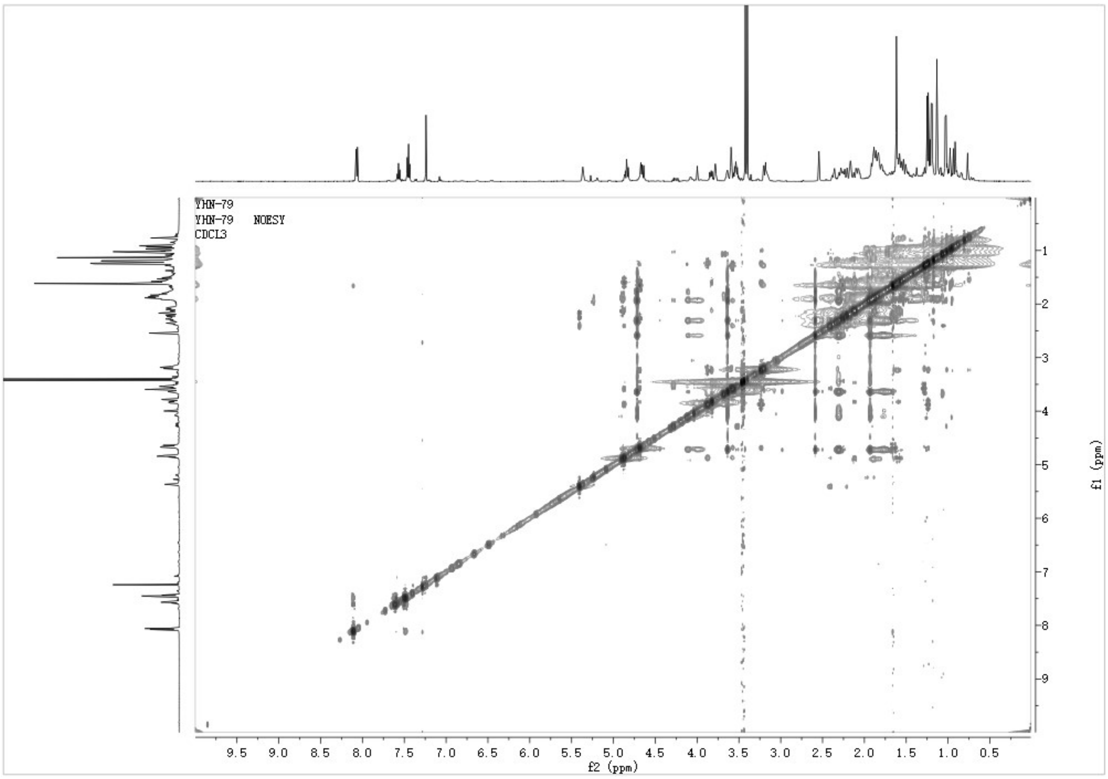

Figure S49. NOESY of **7** in $CDCl_3$

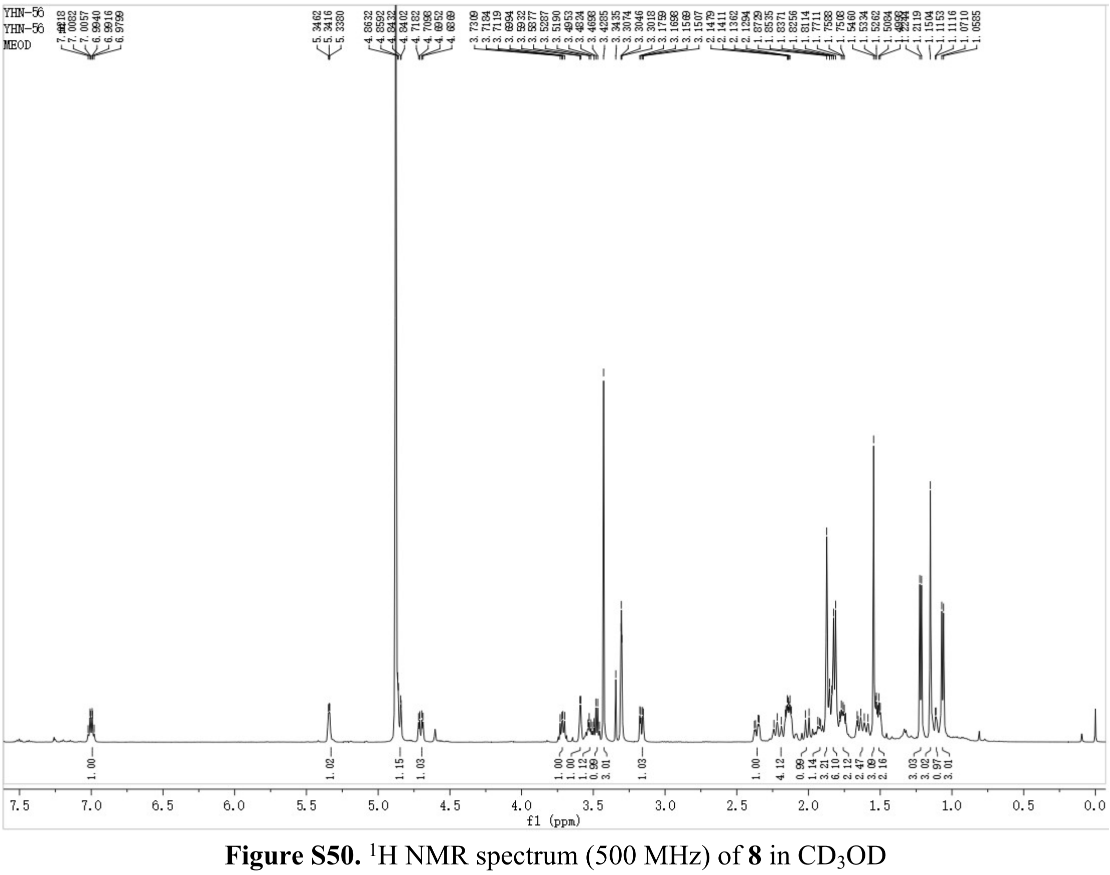

Figure S50. ^{1}H NMR spectrum (500 MHz) of **8** in CD_3OD

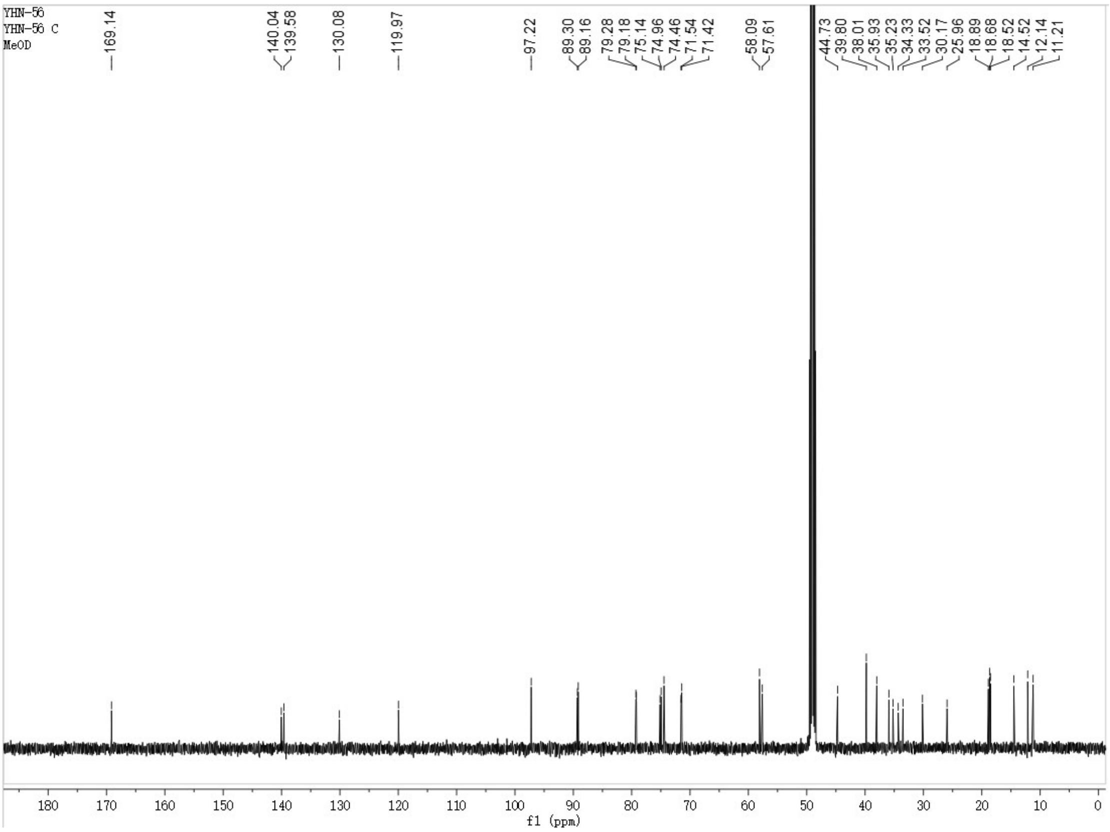

Figure S51.^{13}C NMR spectrum (125 MHz) of **8** in CD_3OD

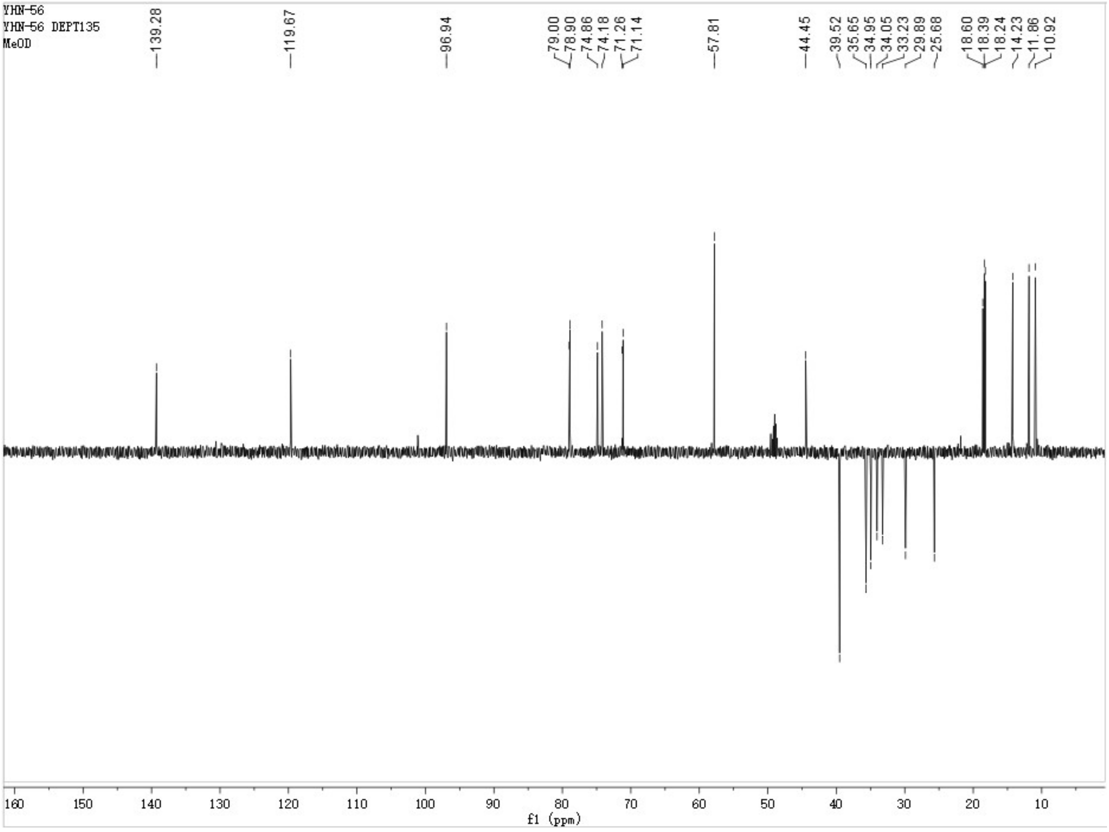

Figure S52. DEPT spectrum (125 MHz) of **8** in CD_3OD

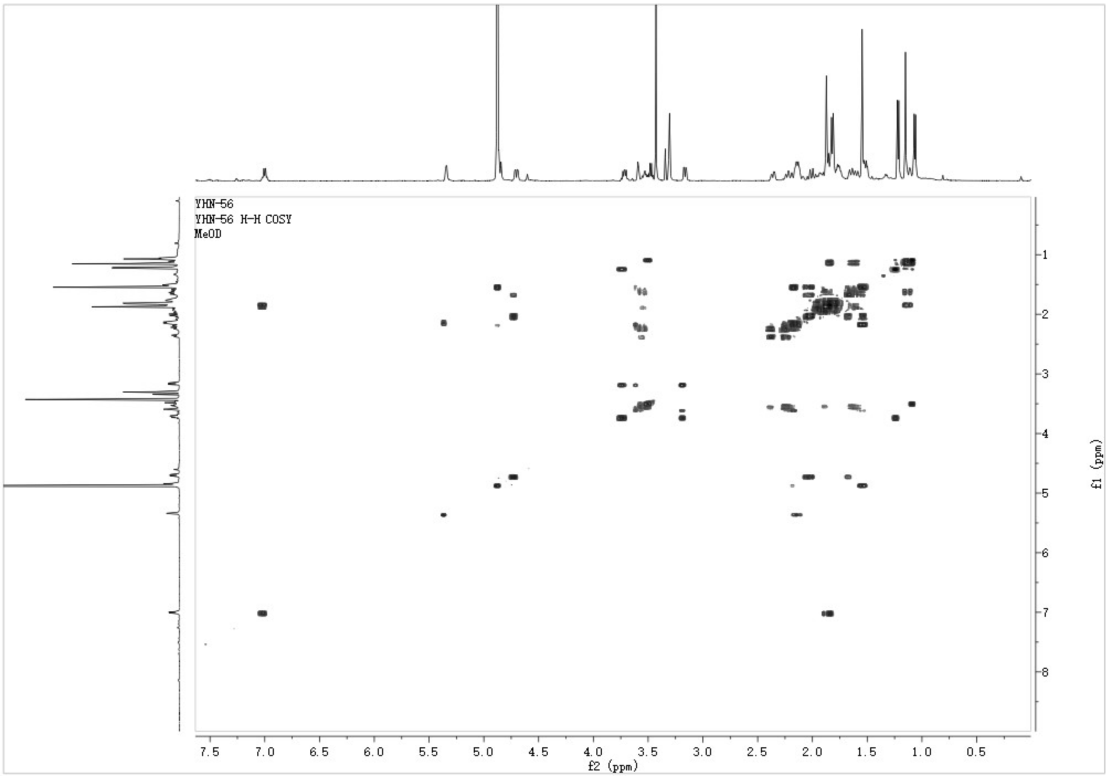

Figure S53. ^{1}H-^{1}H COSY of **8** in CD_3OD

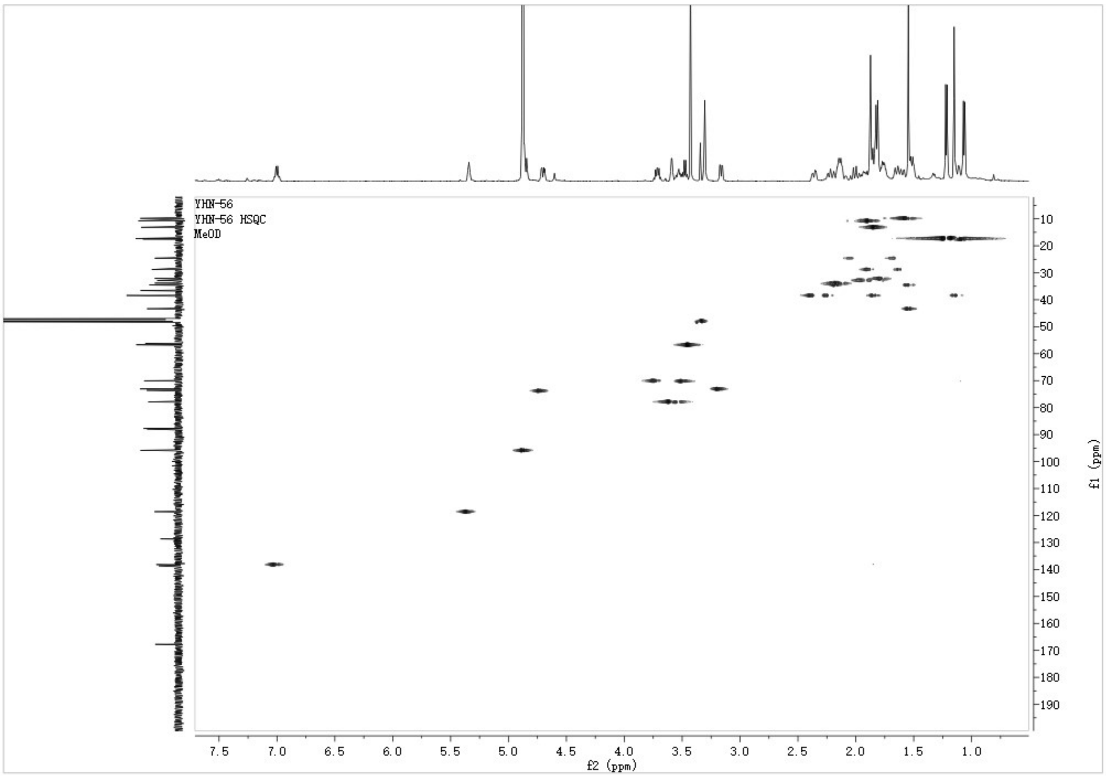

Figure S54. HSQC of **8** in CD_3OD

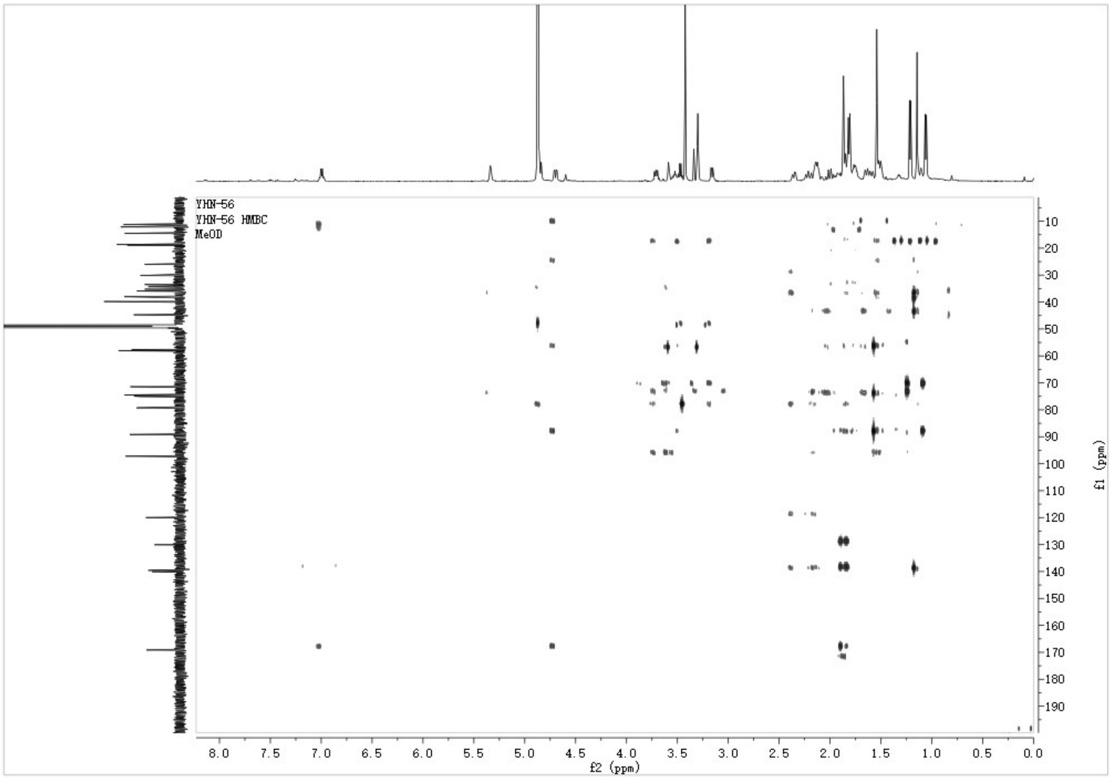

Figure S55. HMBC of **8** in CD_3OD

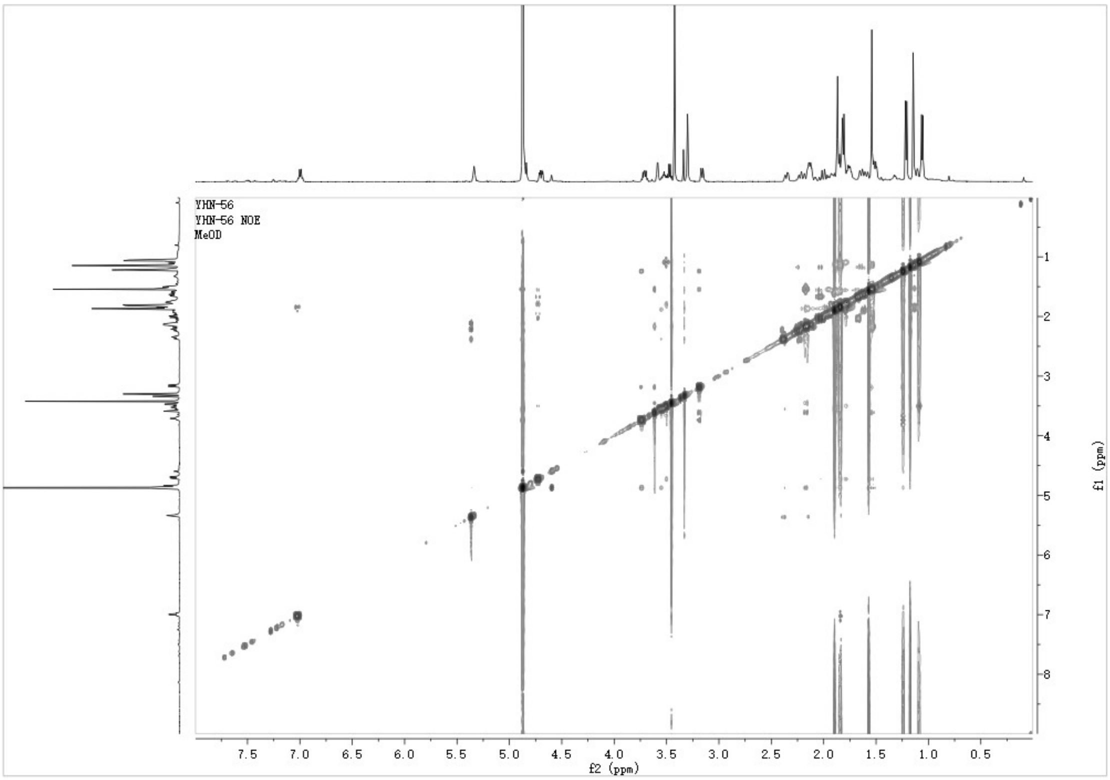

Figure S56. NOESY of **8** in CD_3OD

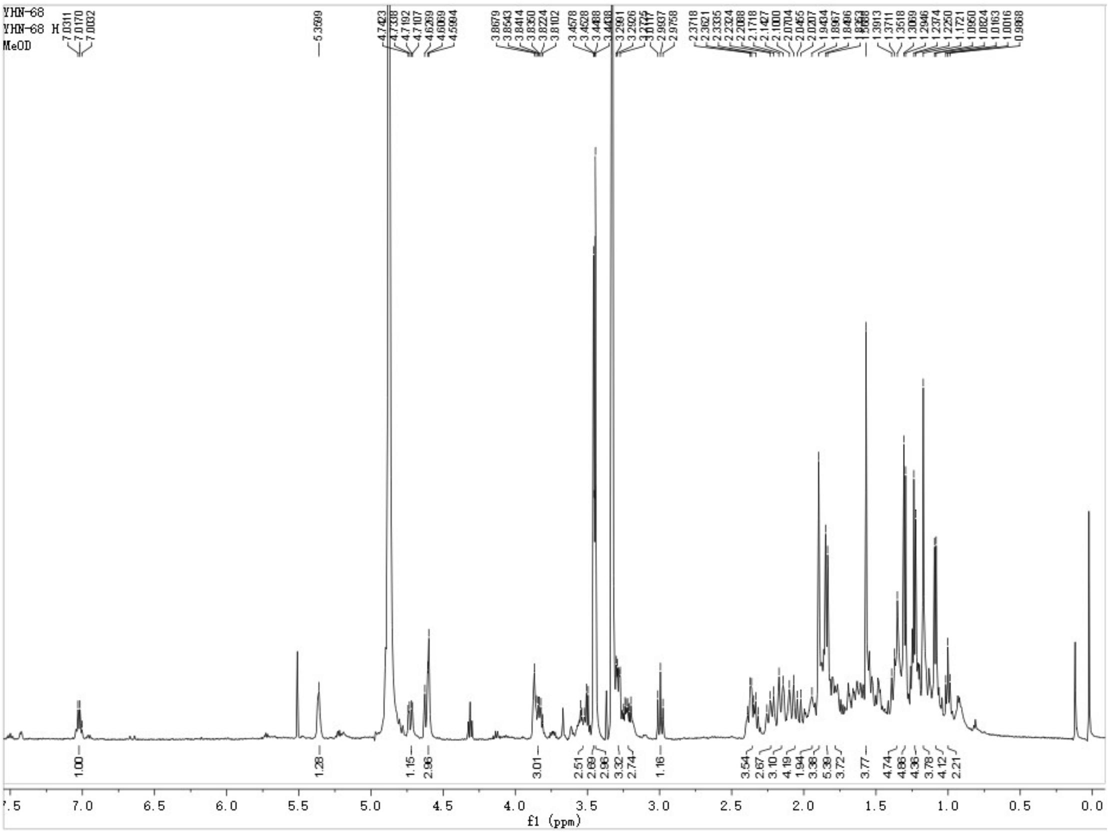

Figure S57. ^{1}H NMR spectrum (500 MHz) of **9** in CD_3OD

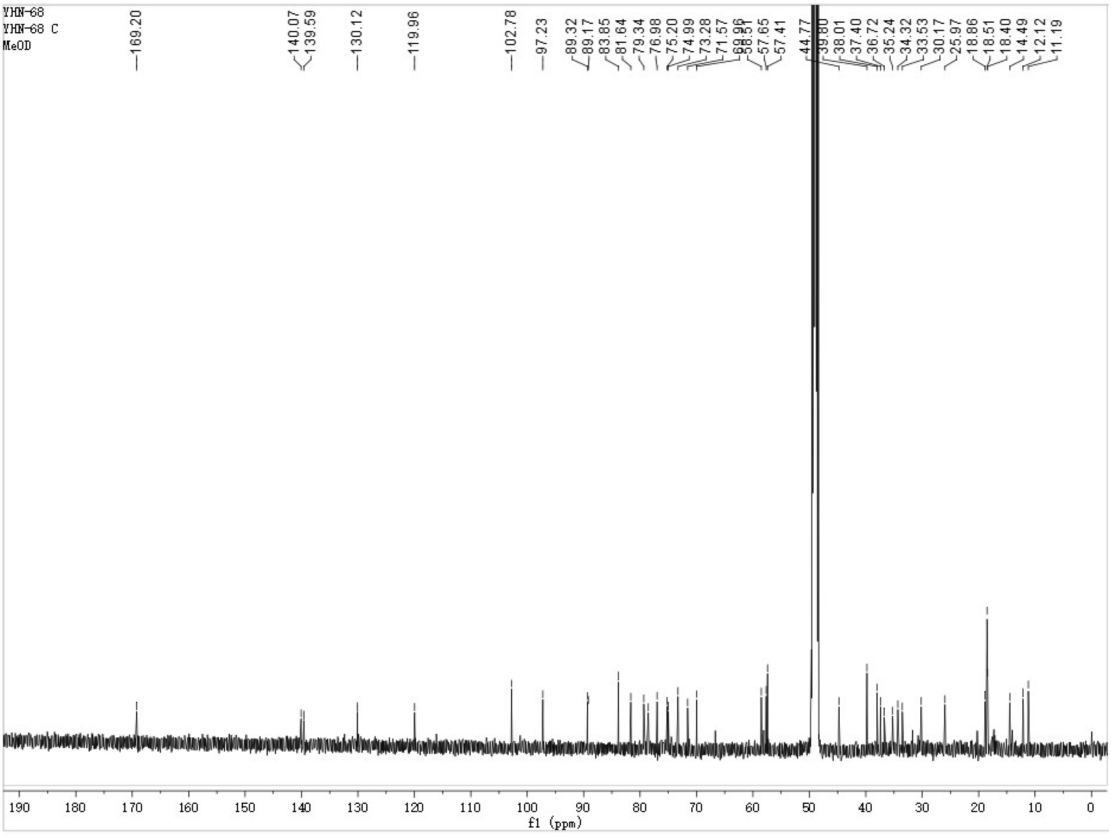

Figure S58. ^{13}C NMR spectrum (125 MHz) of **9** in CD_3OD

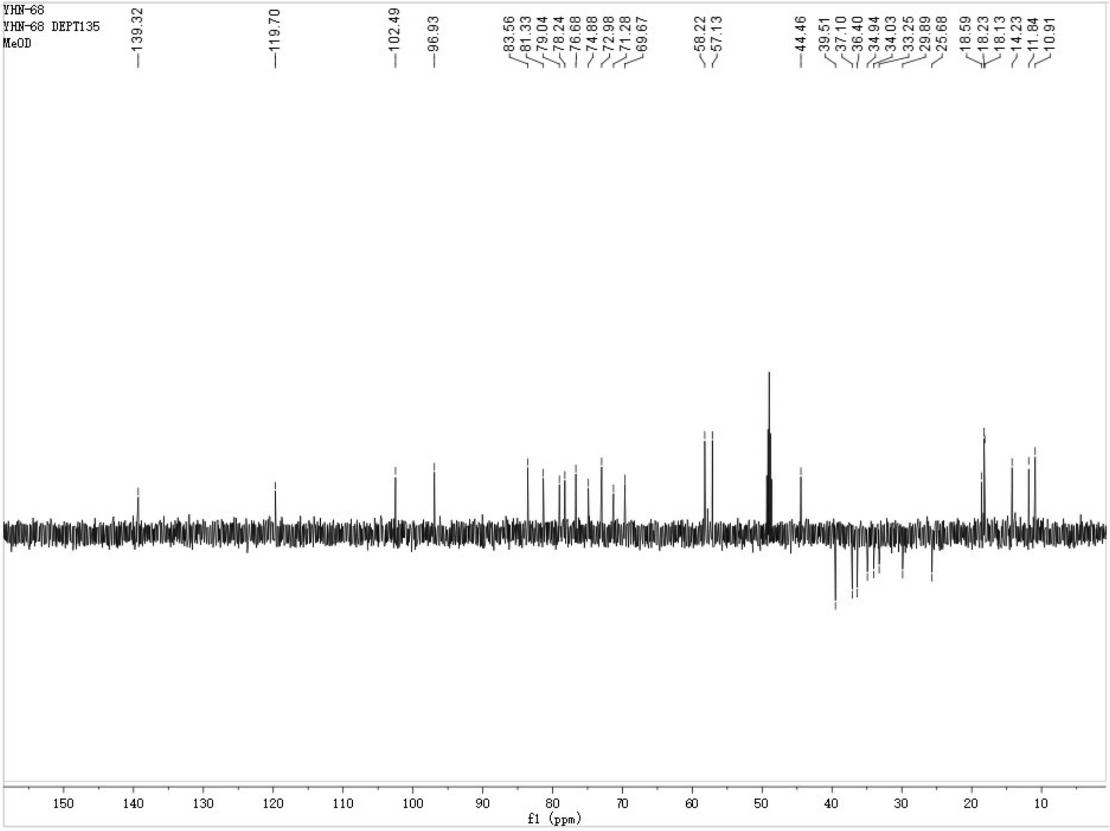

Figure S59. DEPT spectrum of **9** in CD_3OD

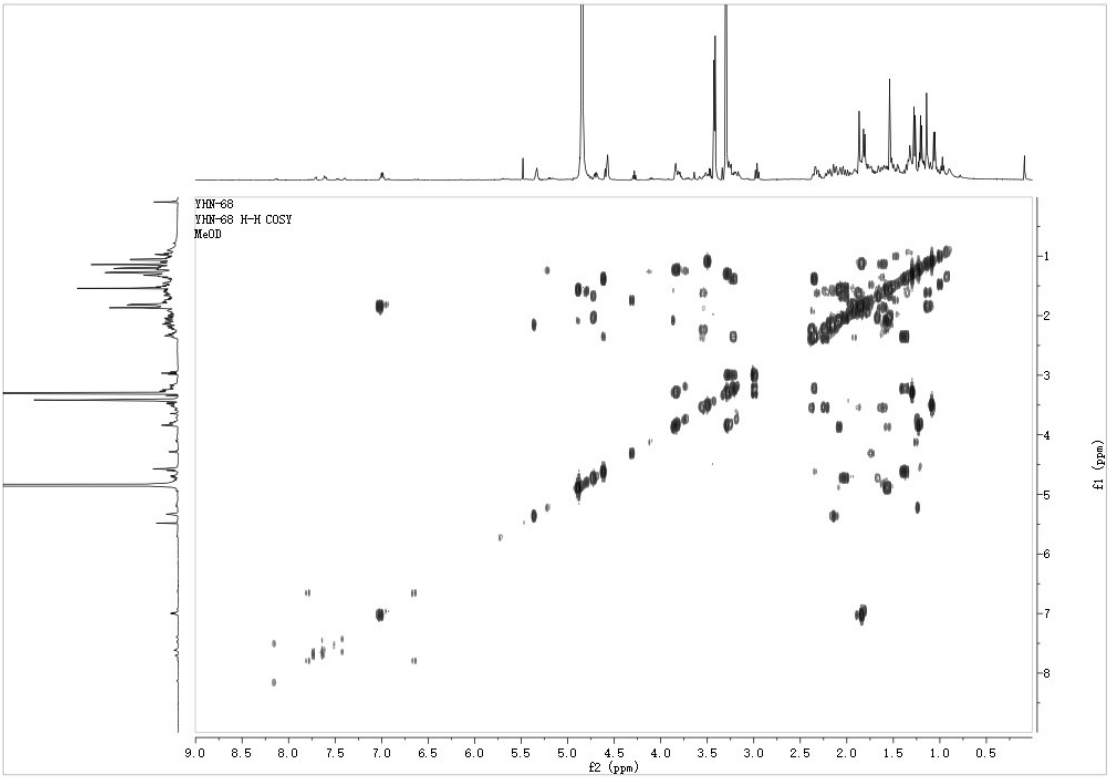

Figure S60. ^{1}H-^{1}H COSY of **9** in CD_3OD

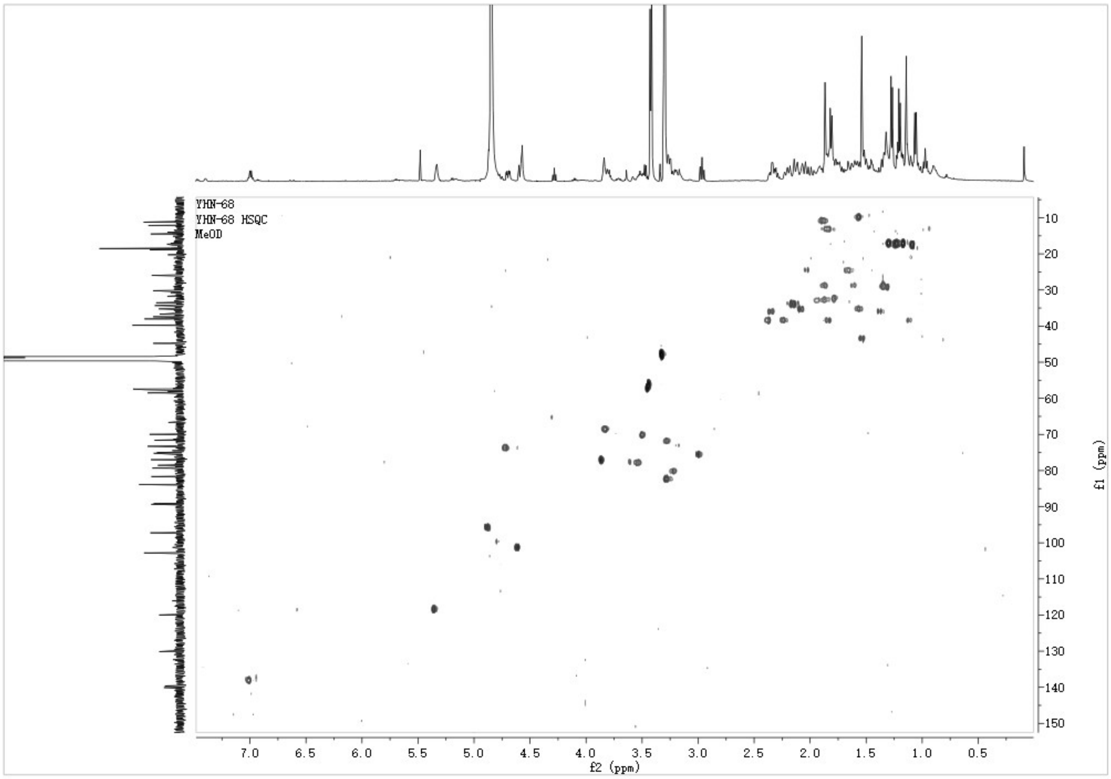

Figure S61. HSQC of **9** in CD_3OD

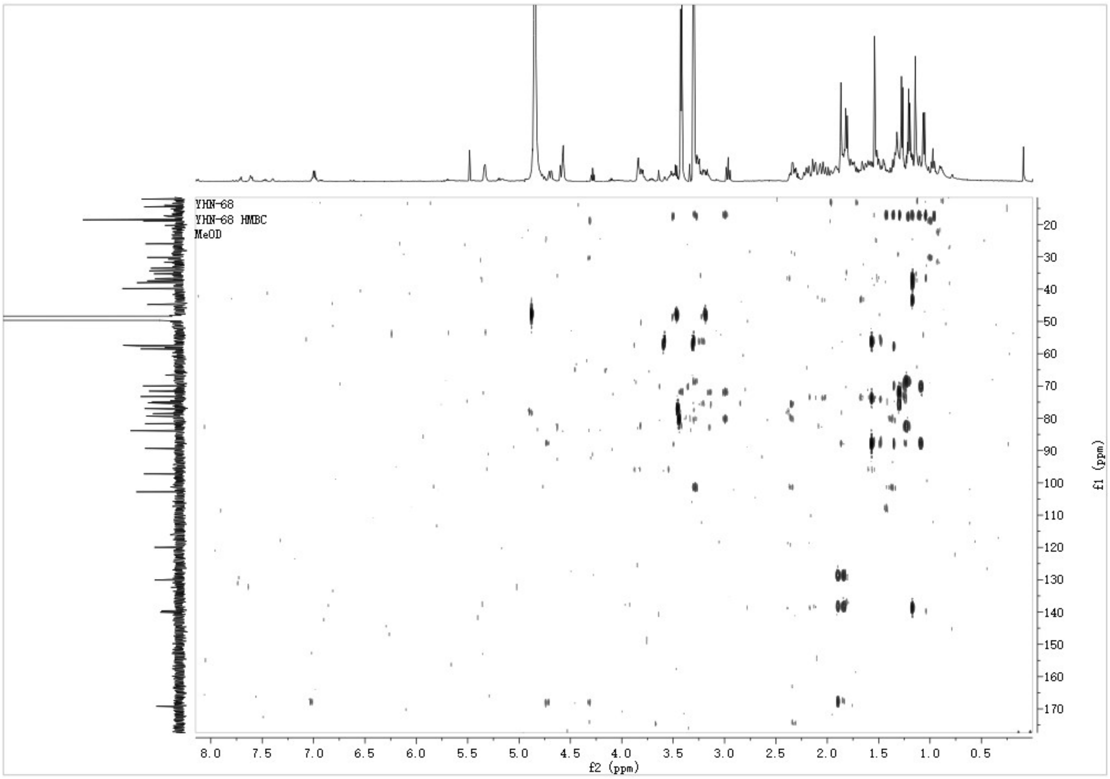

Figure S62. HMBC of **9** in CD_3OD

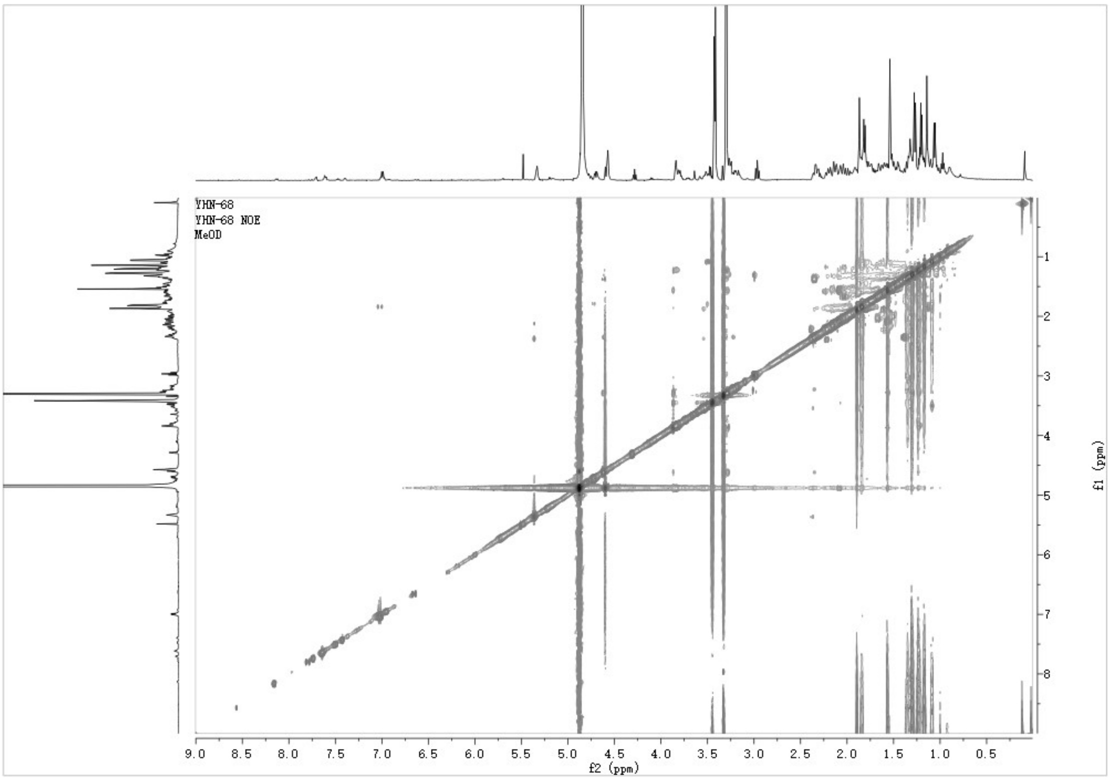

Figure S63. NOESY of **9** in CD_3OD

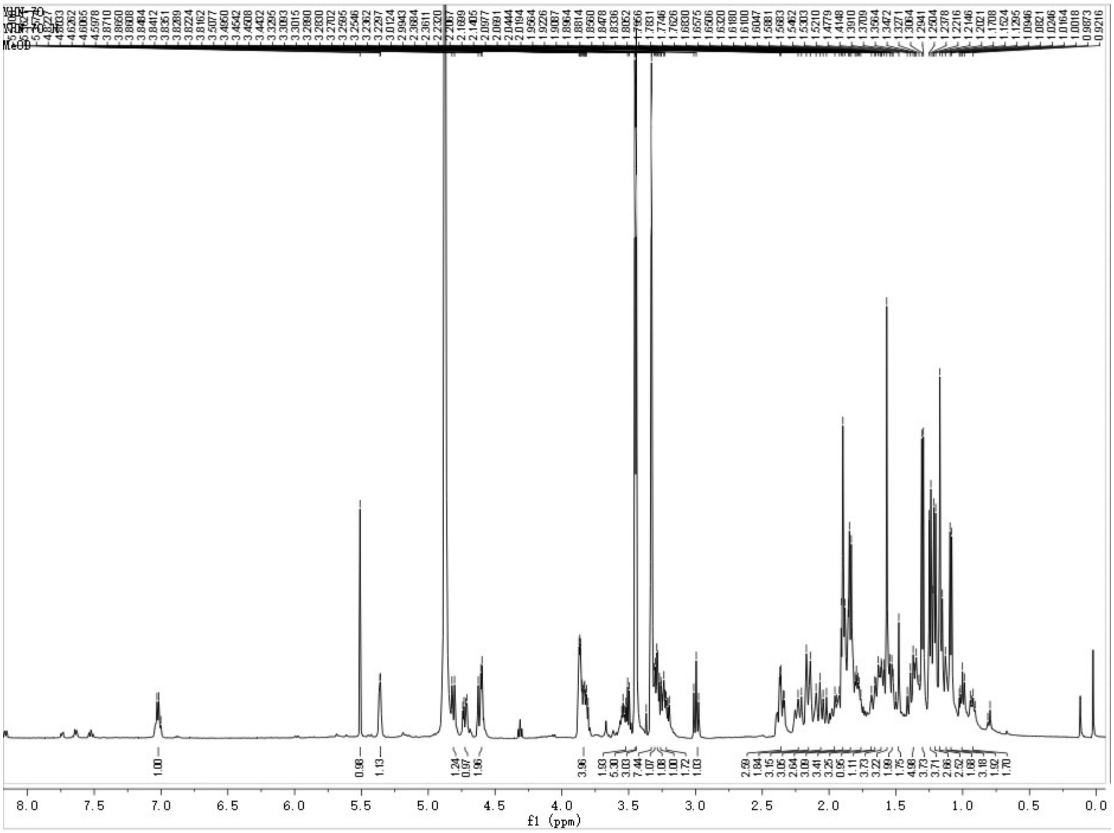

Figure S64. ^{1}H NMR spectrum (500 MHz) of **10** in CD_3OD

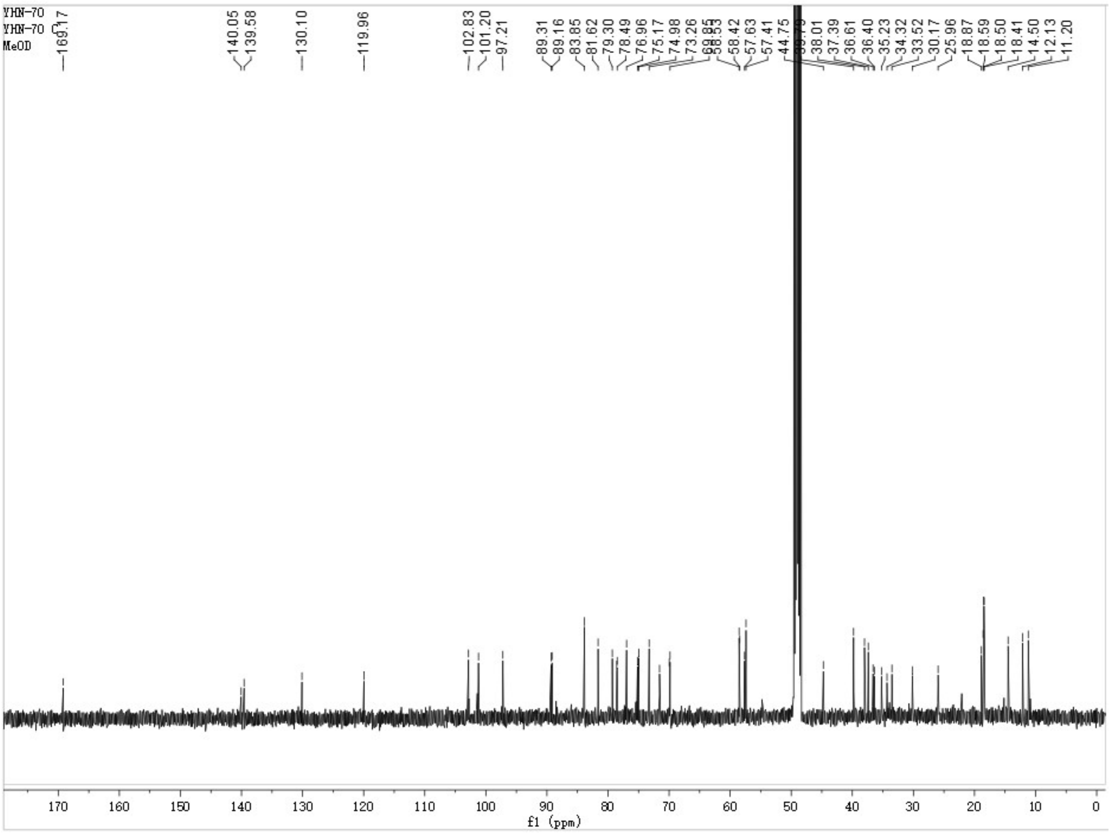

Figure S65. ^{13}C NMR spectrum (125 MHz) of **10** in CD_3OD

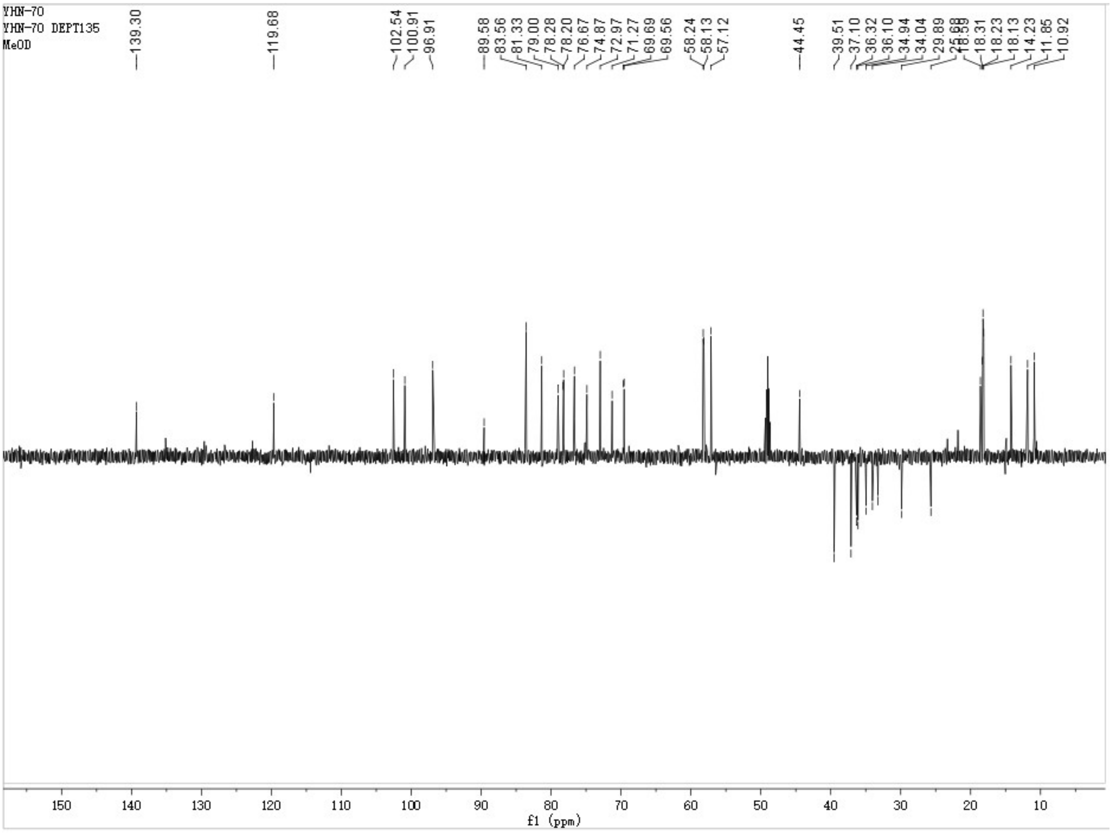

Figure S66. DEPT spectrum of **10** in CD_3OD

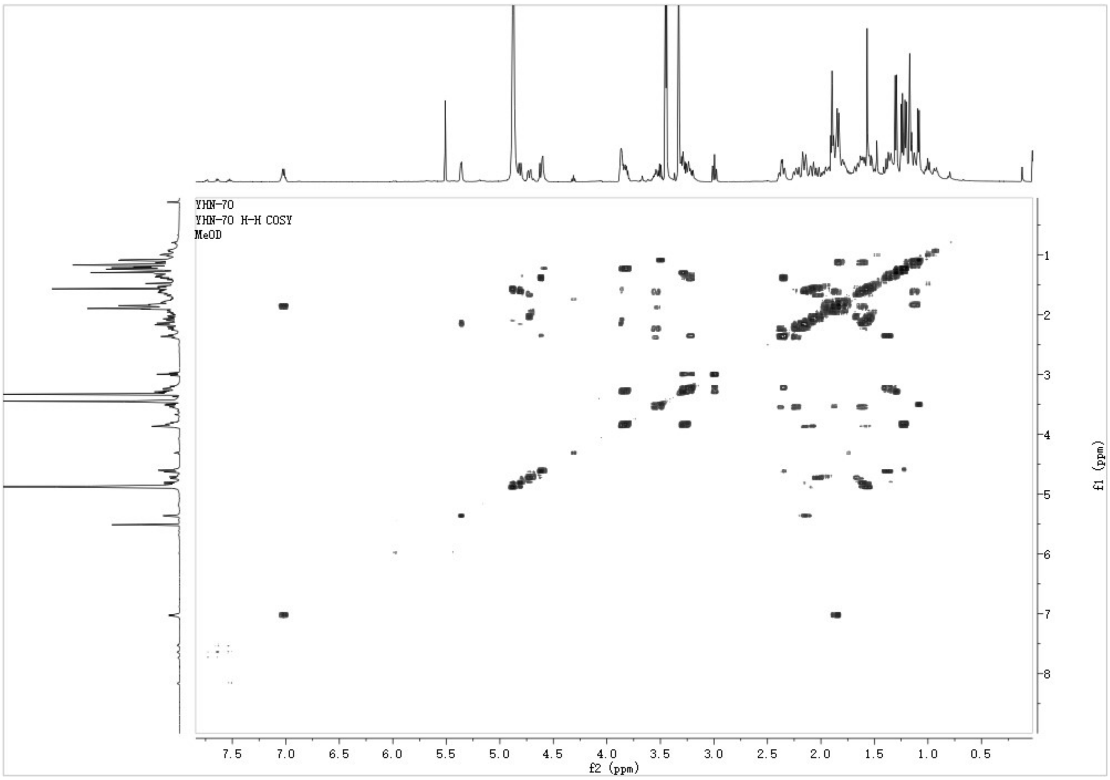

Figure S67. ^{1}H-^{1}H COSY of **10** in CD_3OD

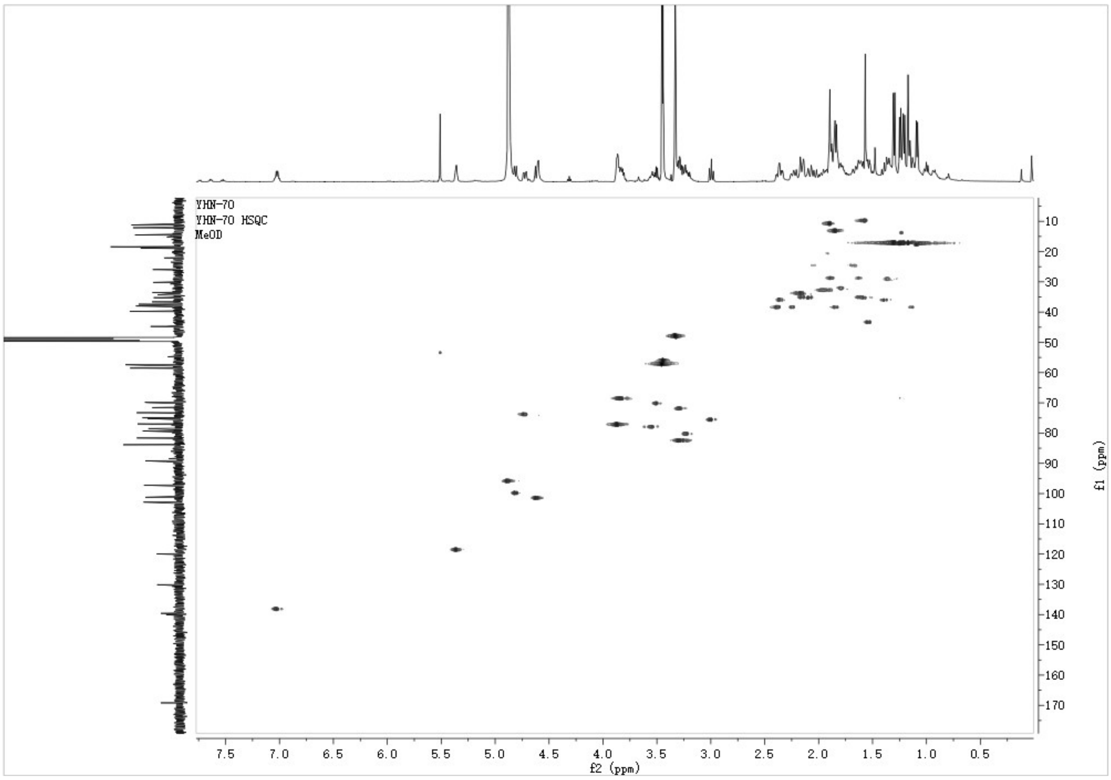

Figure S68. HSQC of **10** in CD_3OD

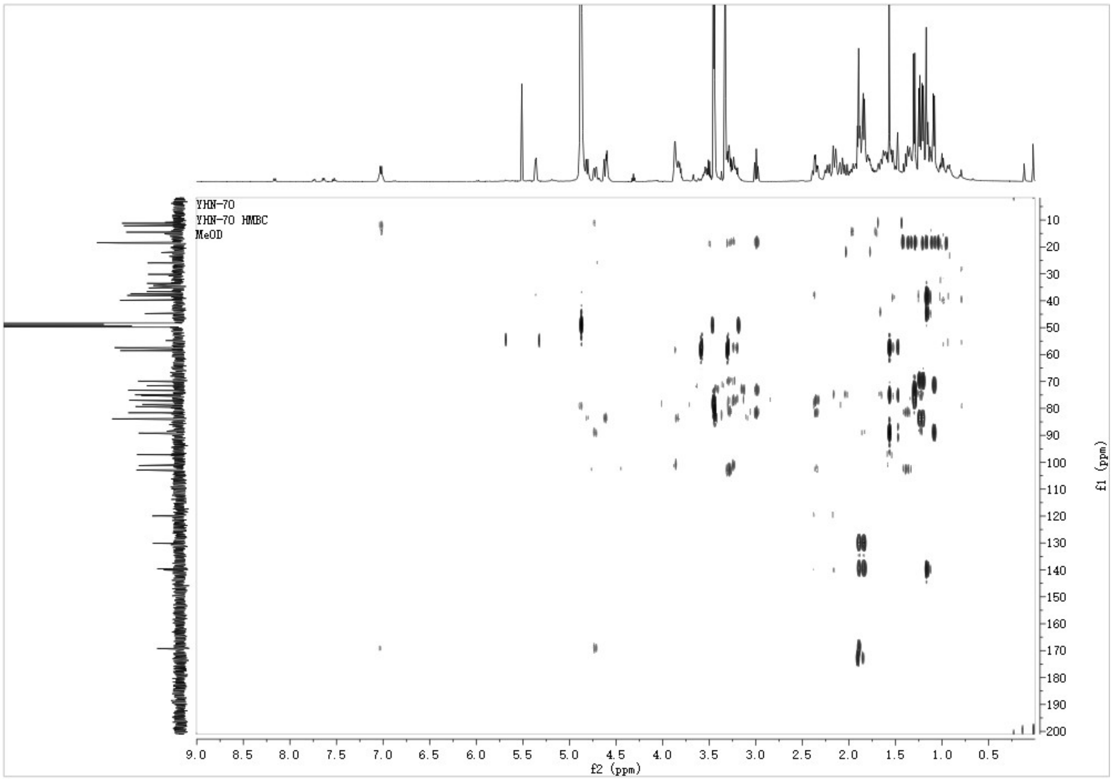

Figure S69. HMBC of **10** in CD_3OD

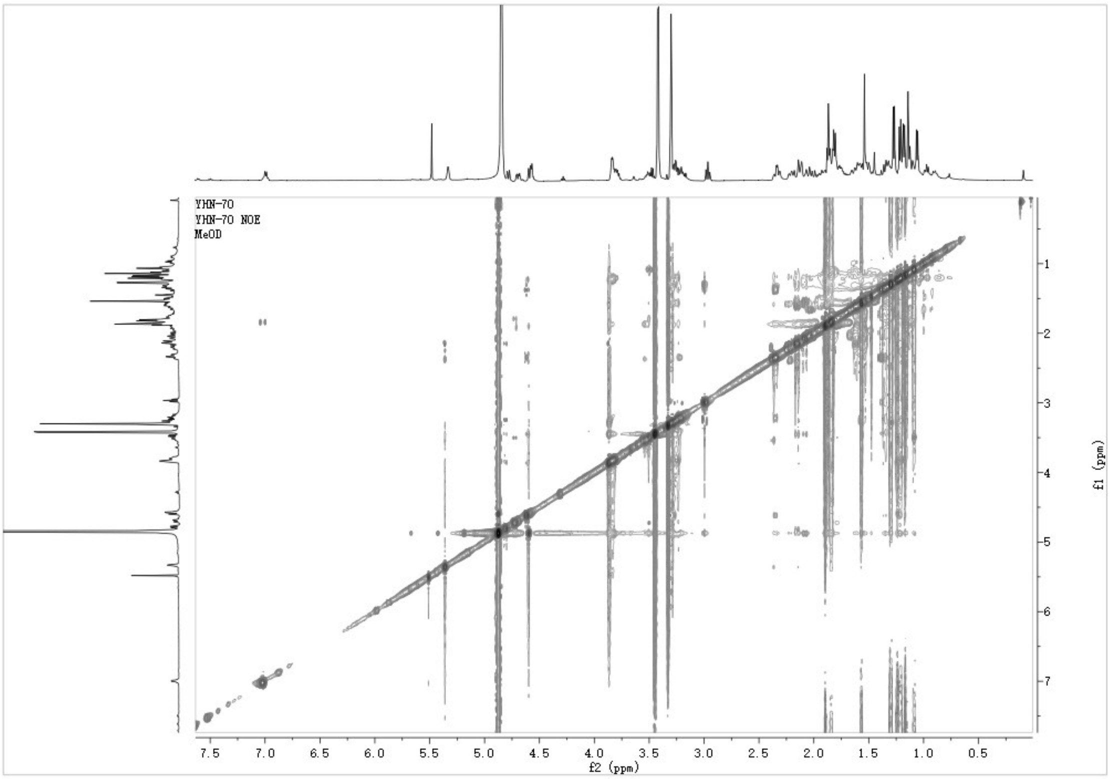

Figure S70. NOESY of **10** in CD_3OD

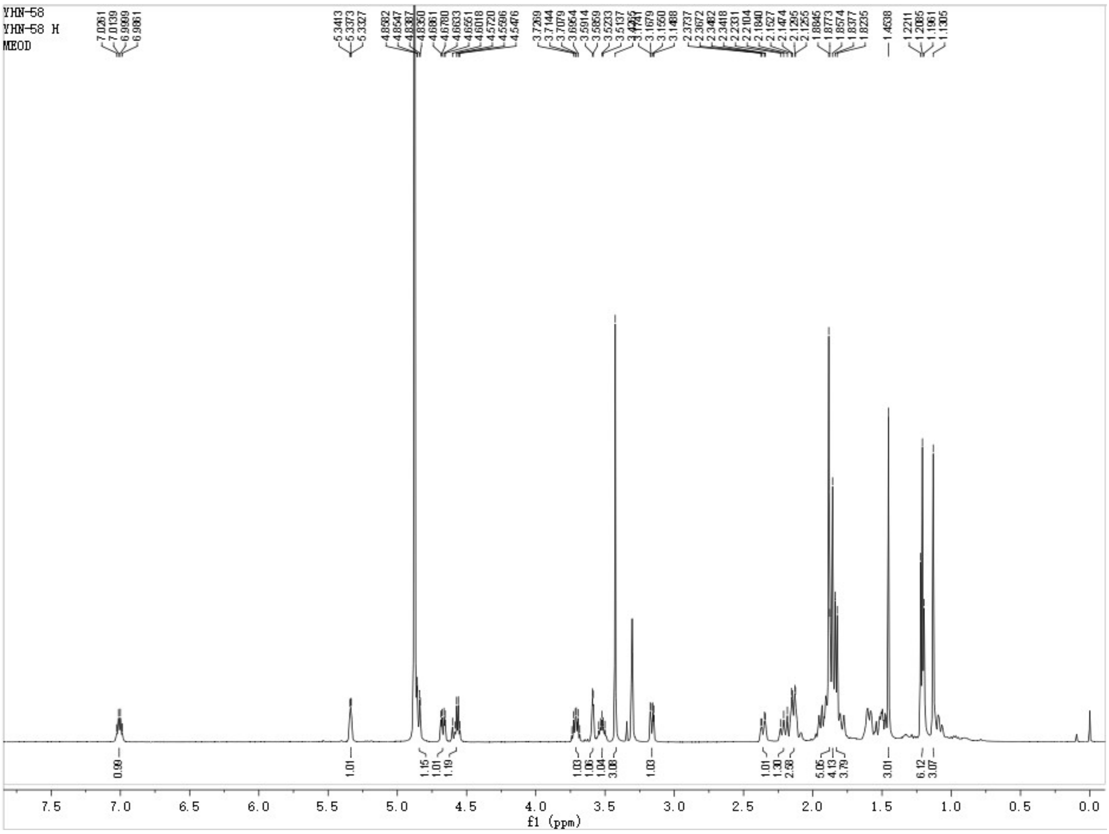

Figure S71. ^{1}H NMR spectrum (500 MHz) of **11** in CD_3OD

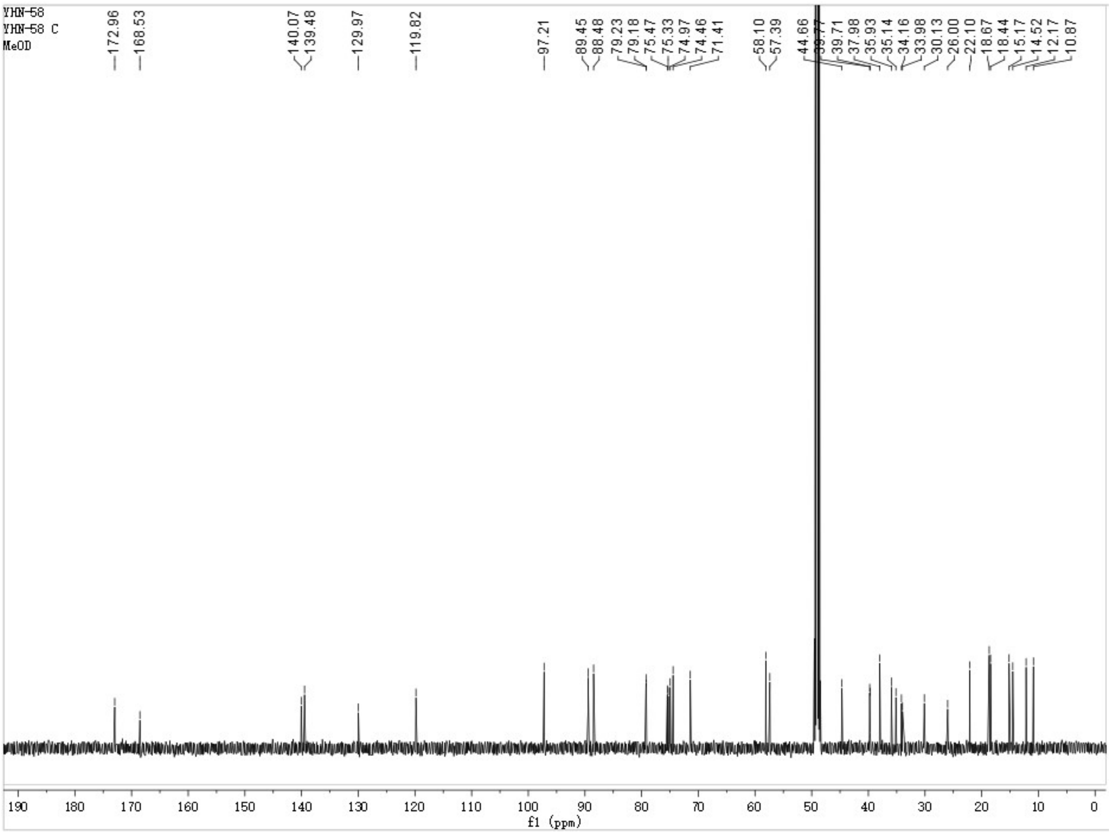

Figure S72. ^{13}C NMR spectrum (125 MHz) of **11** in CD_3OD

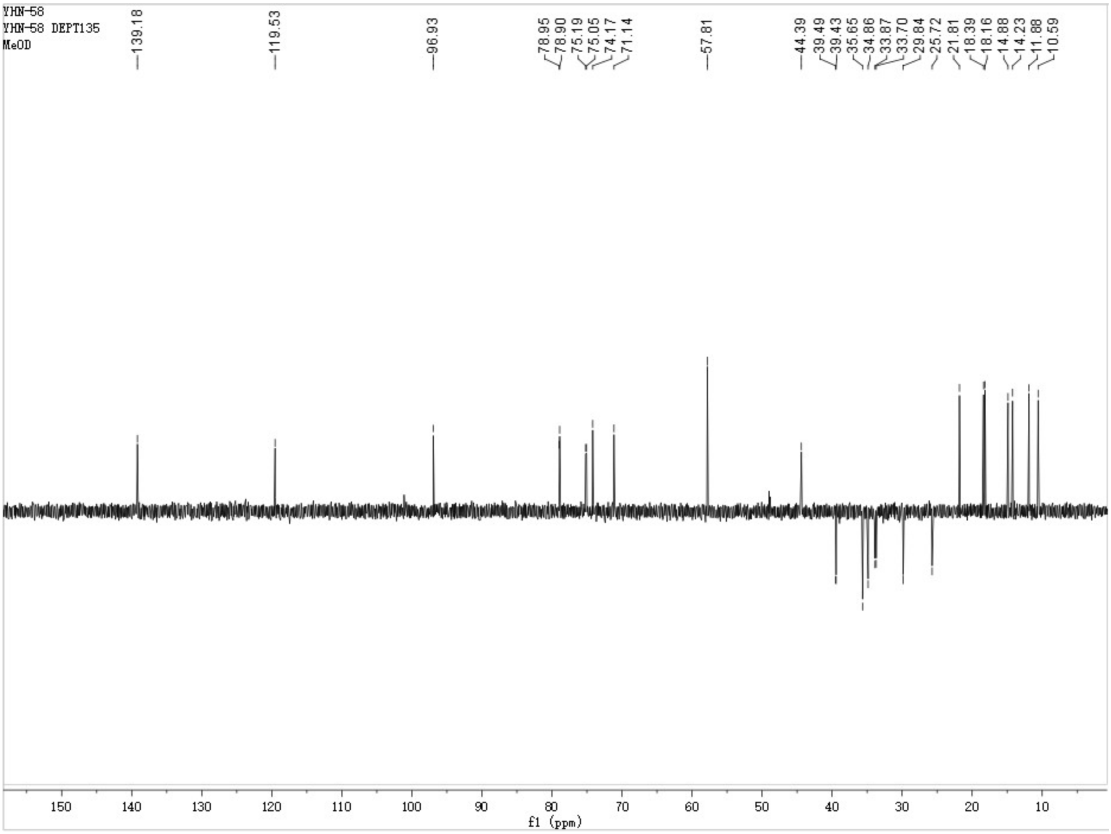

Figure S73. DEPT spectrum (125 MHz) of **11** in CD_3OD

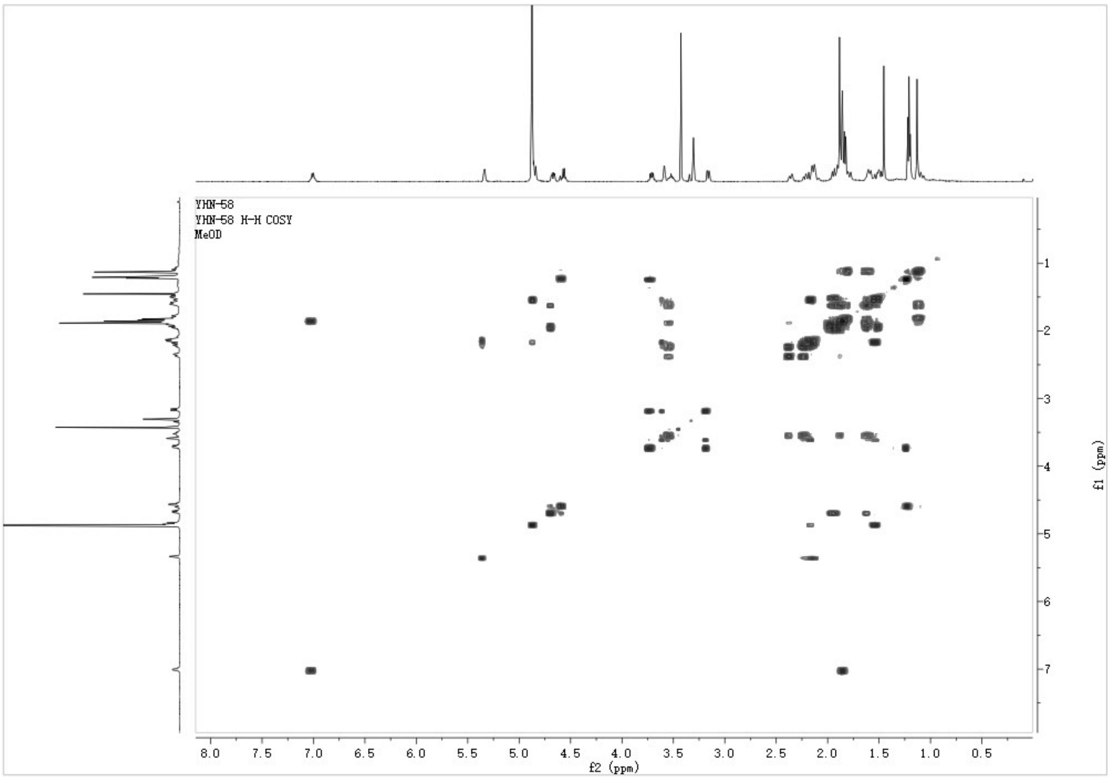

Figure S74. ^{1}H-^{1}H COSY (500 MHz) of **11** in CD_3OD

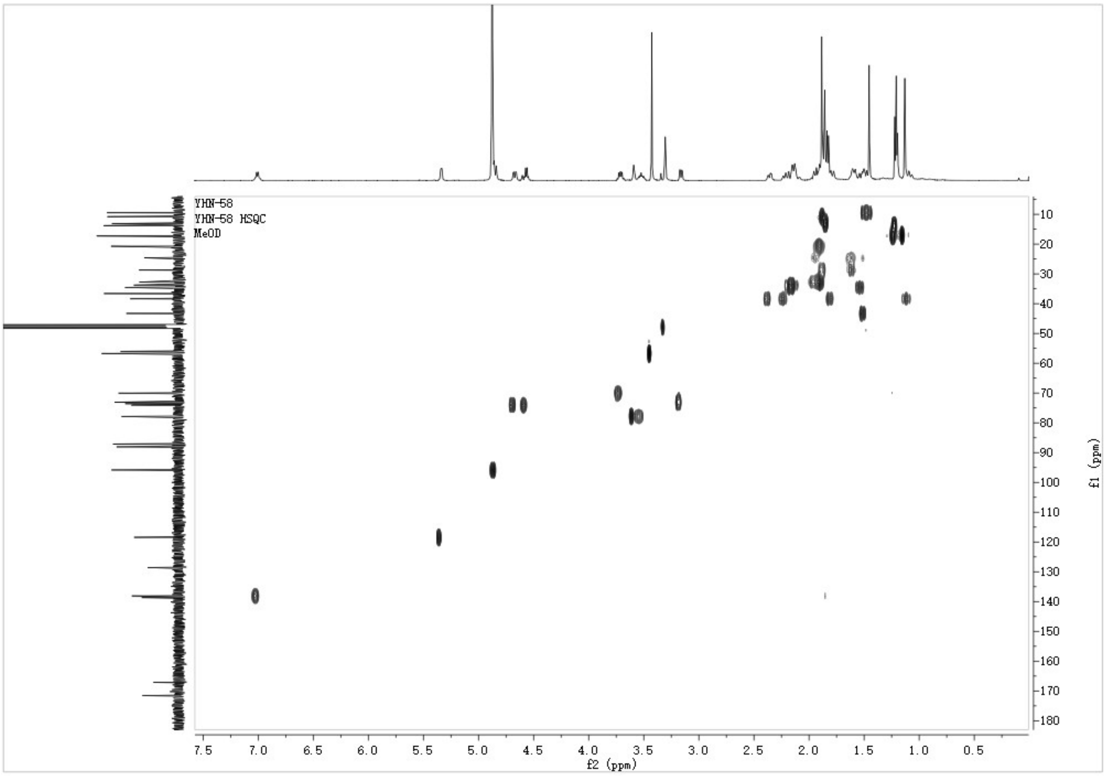

Figure S75. HSQC of **11** in CD_3OD

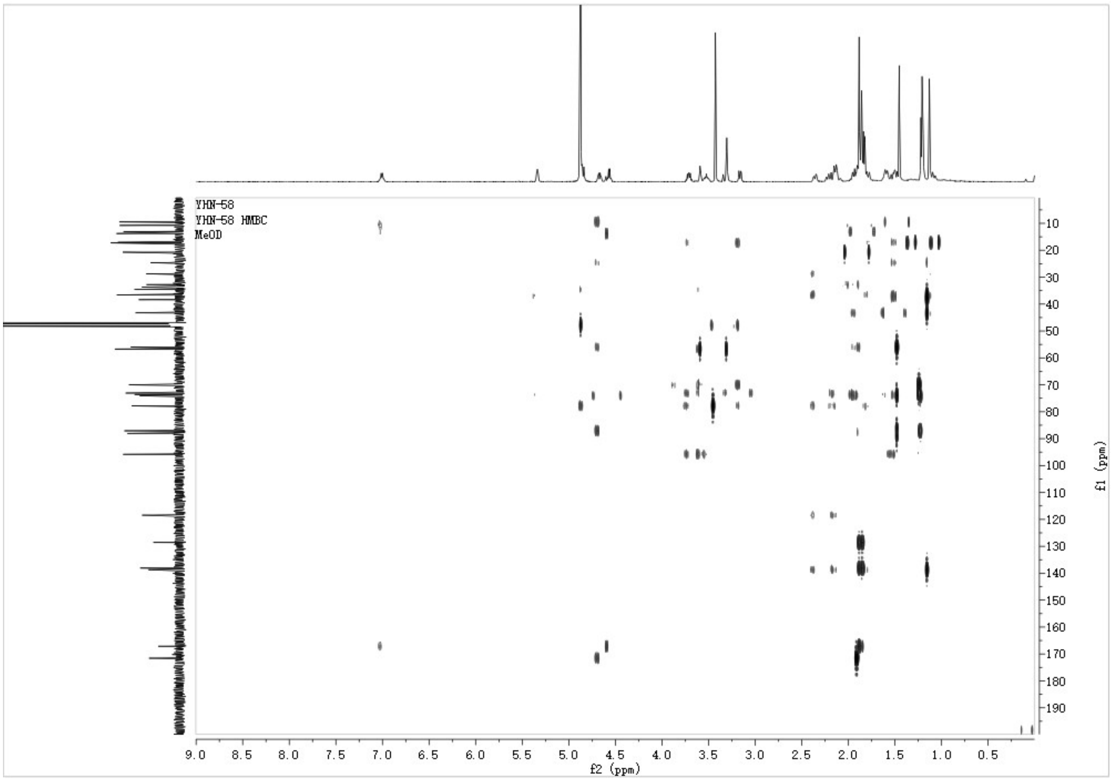

Figure S76. HMBC of **11** in CD_3OD

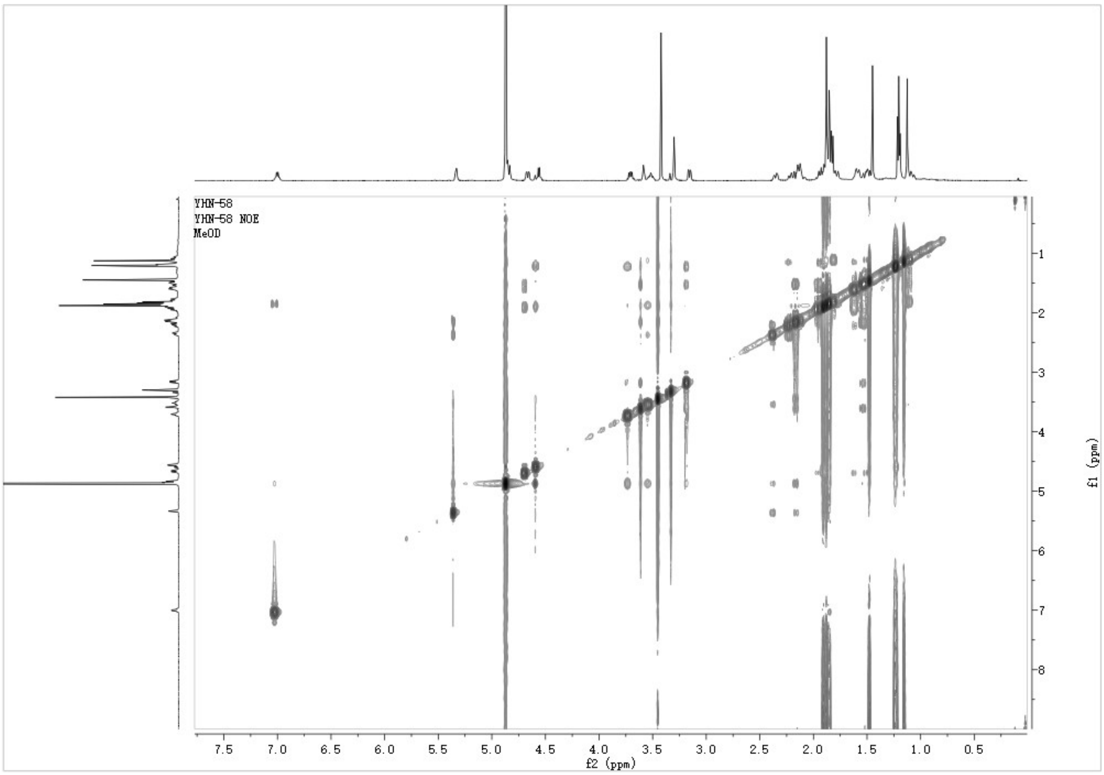

Figure S77. NOESY of **11** in CD_3OD

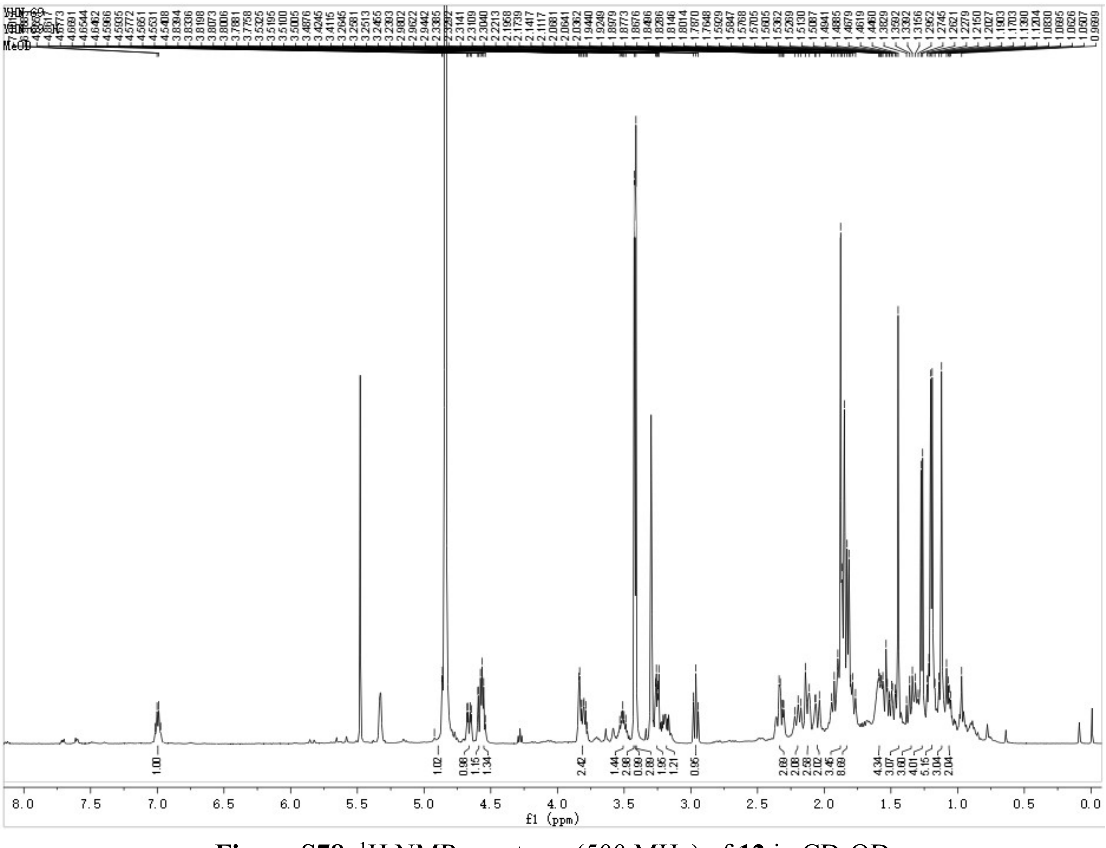

Figure S78. ^{1}H NMR spectrum (500 MHz) of **12** in CD_3OD

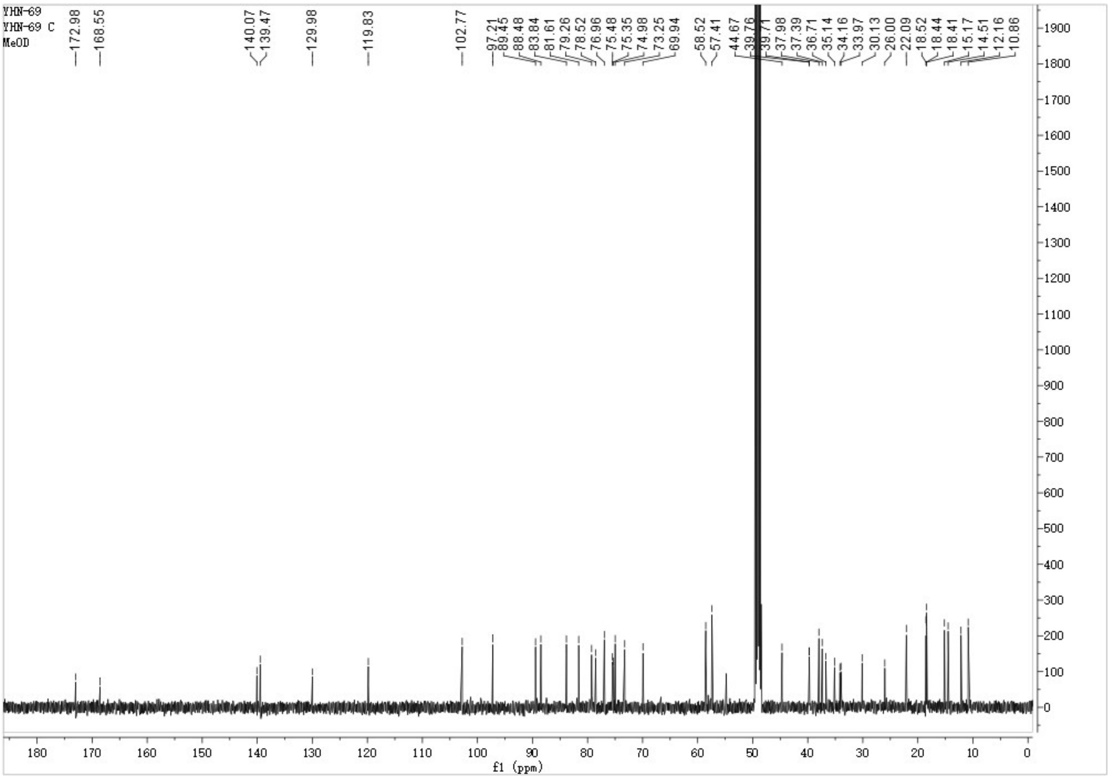

Figure S79. ^{13}C NMR spectrum (125 MHz) of **12** in CD_3OD

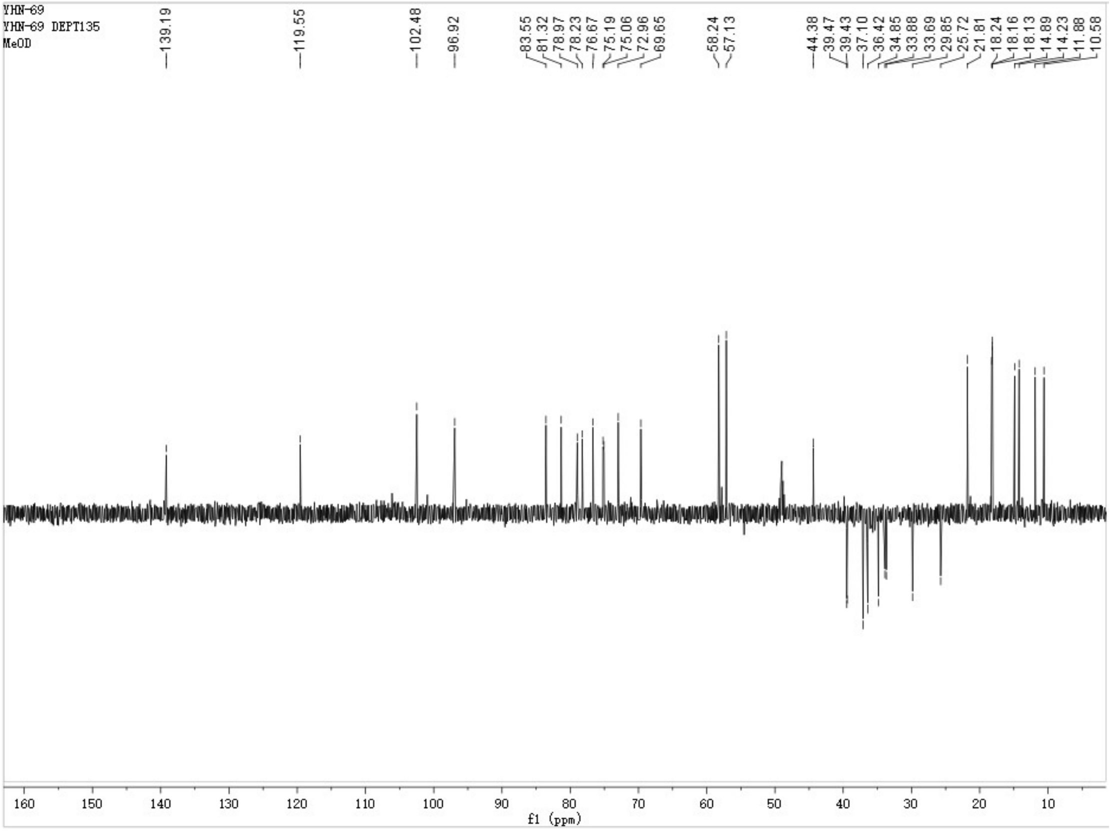

Figure S80. DEPT spectrum of **12** in CD_3OD

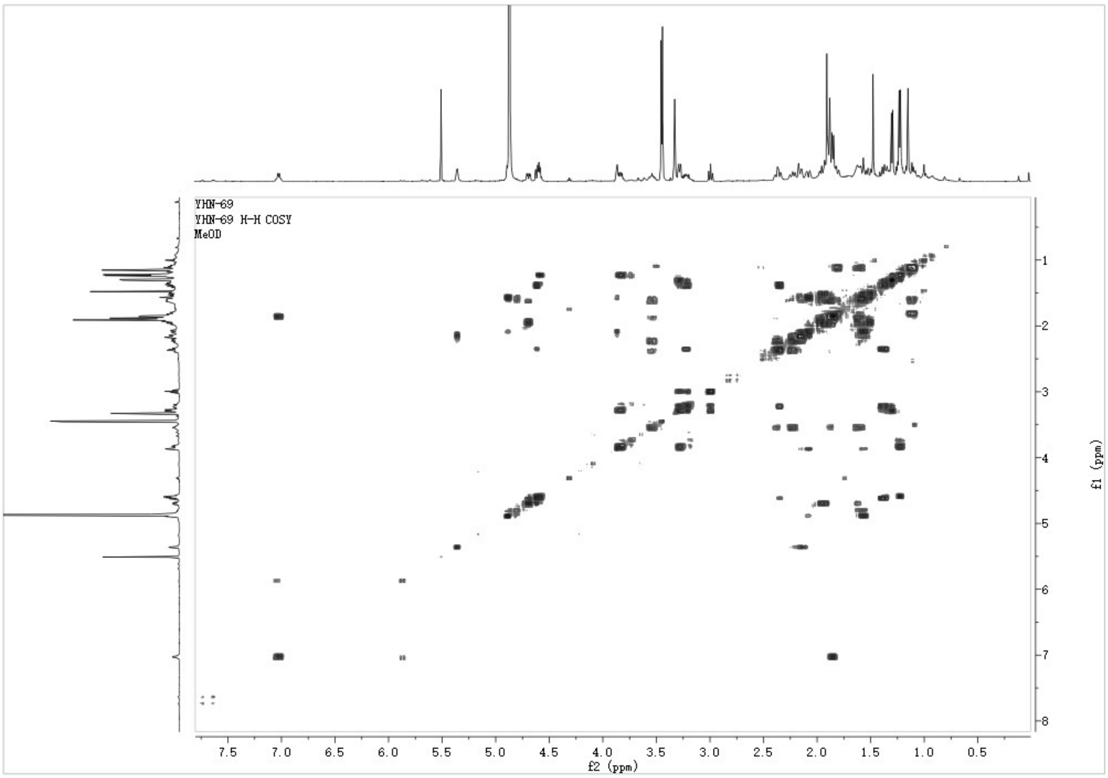

Figure S81. ^{1}H-^{1}H COSY of **12** in CD_3OD

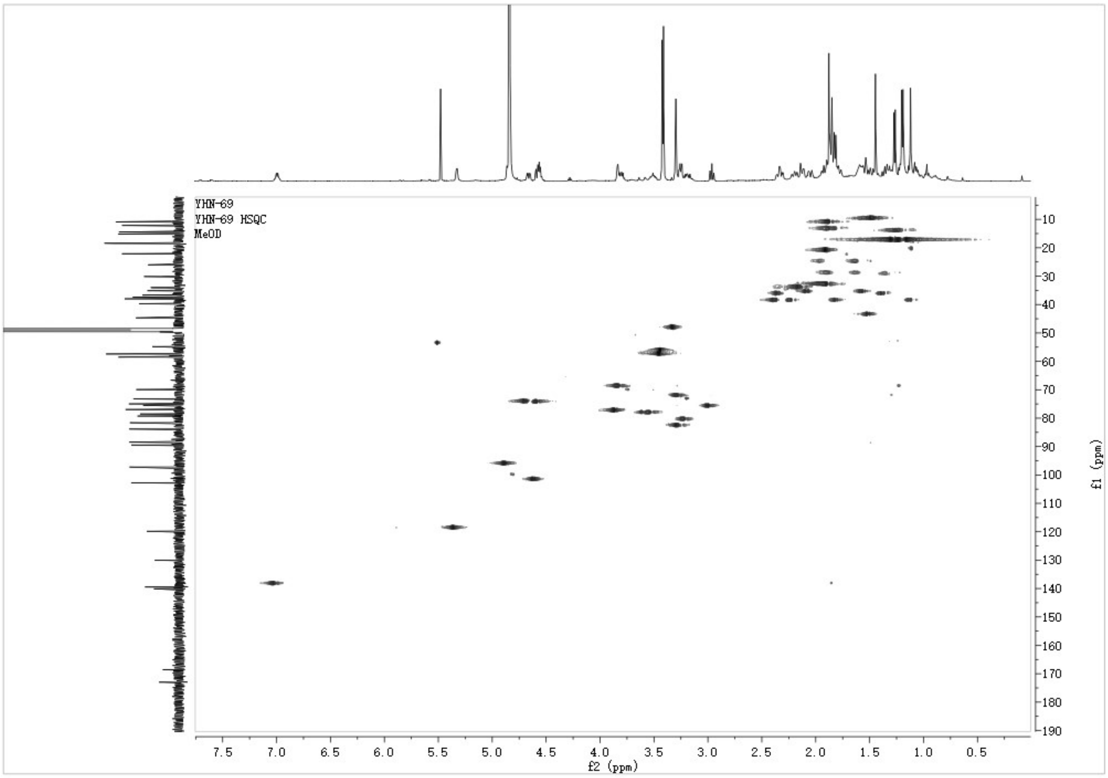

Figure S82. HSQC of **12** in CD_3OD

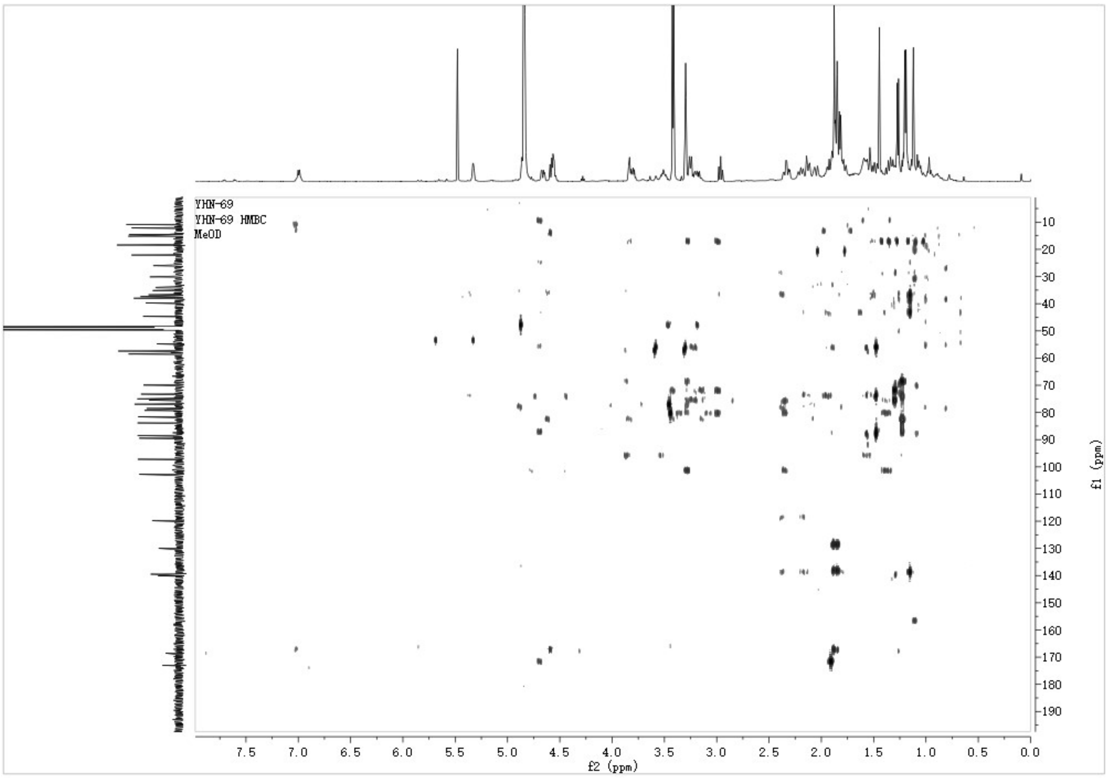

Figure S83. HMBC of **12** in CD_3OD

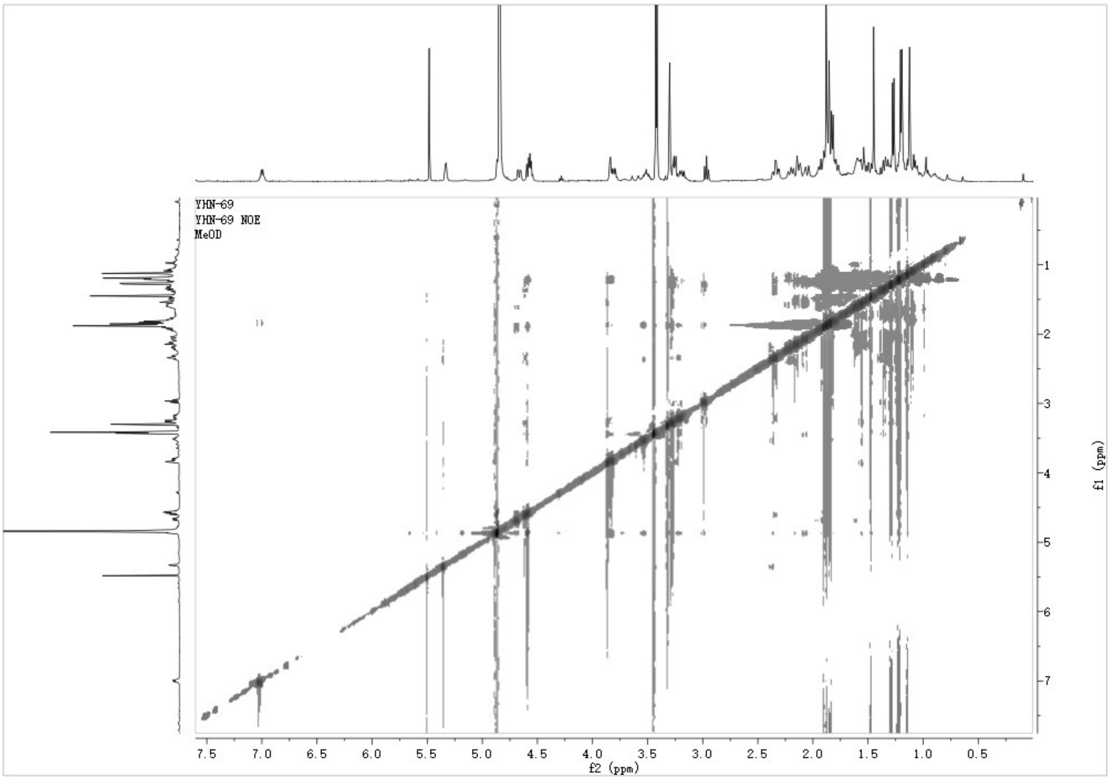

Figure S84. NOESY of **12** in CD_3OD

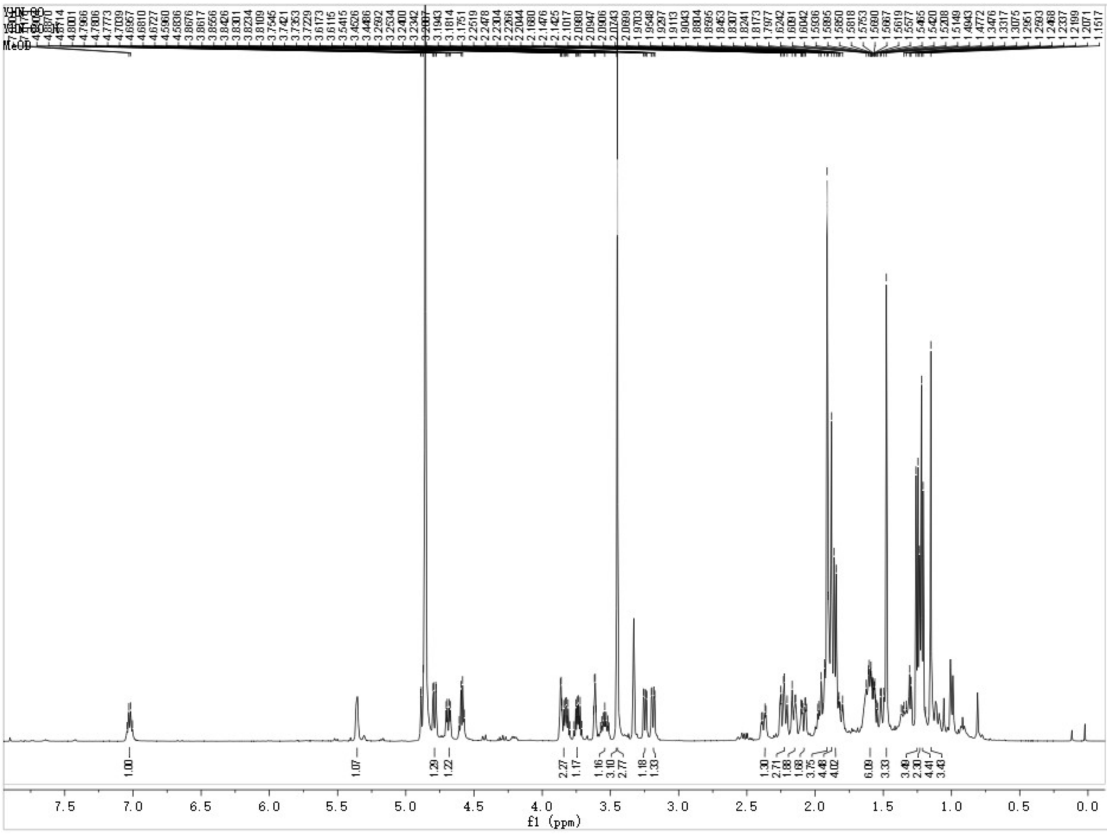

Figure S85. ^{1}H NMR spectrum (500 MHz) of **13** in CD_3OD

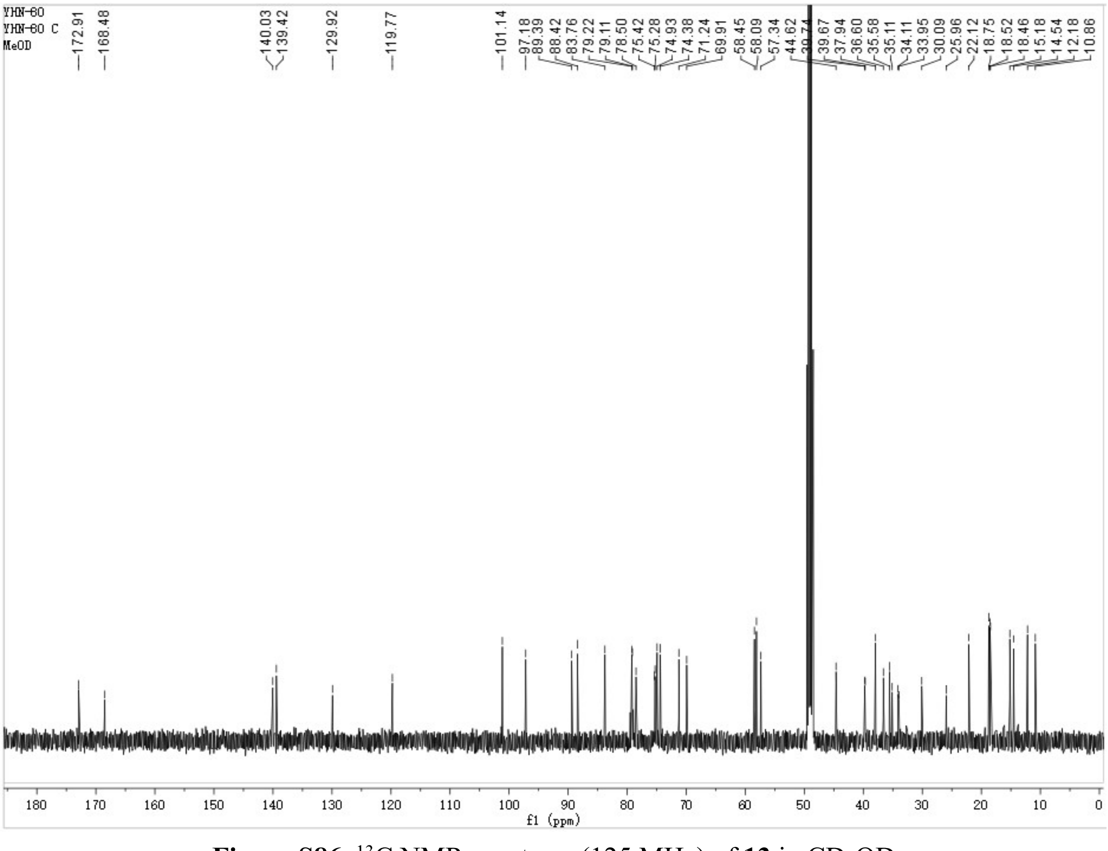

Figure S86. ^{13}C NMR spectrum (125 MHz) of **13** in CD_3OD

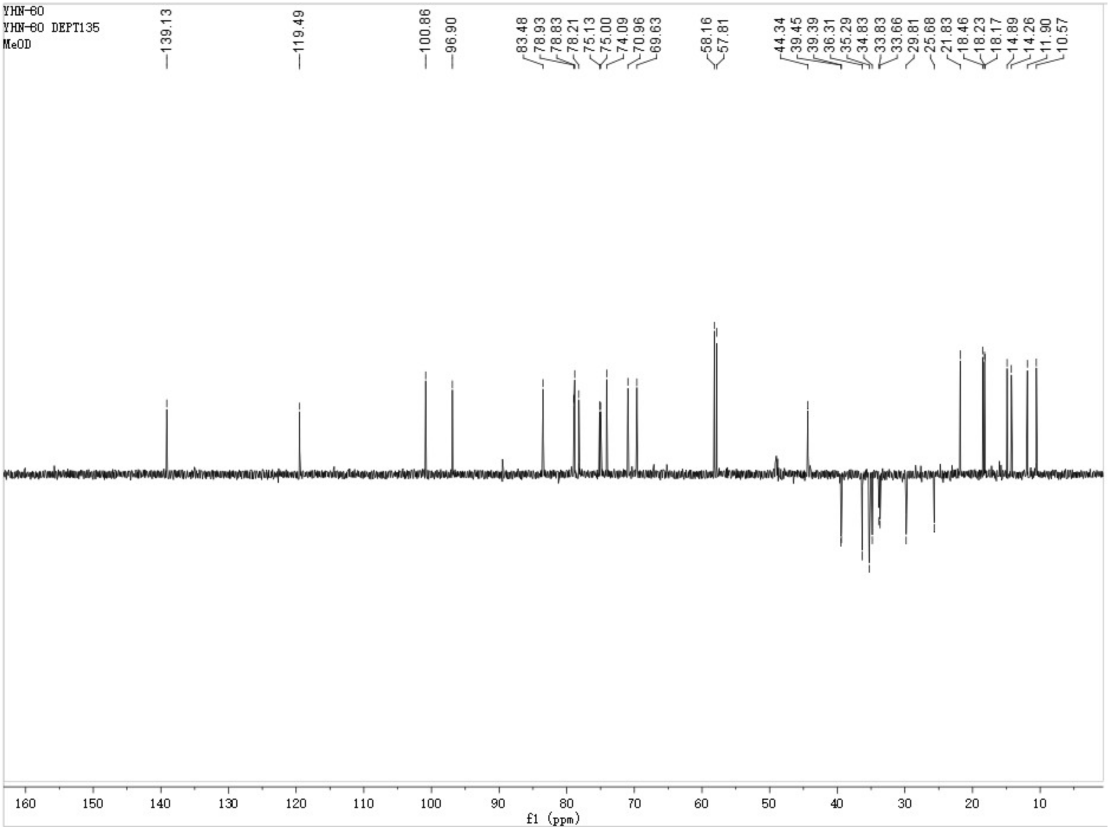

Figure S87. DEPT spectrum of **13** in CD_3OD

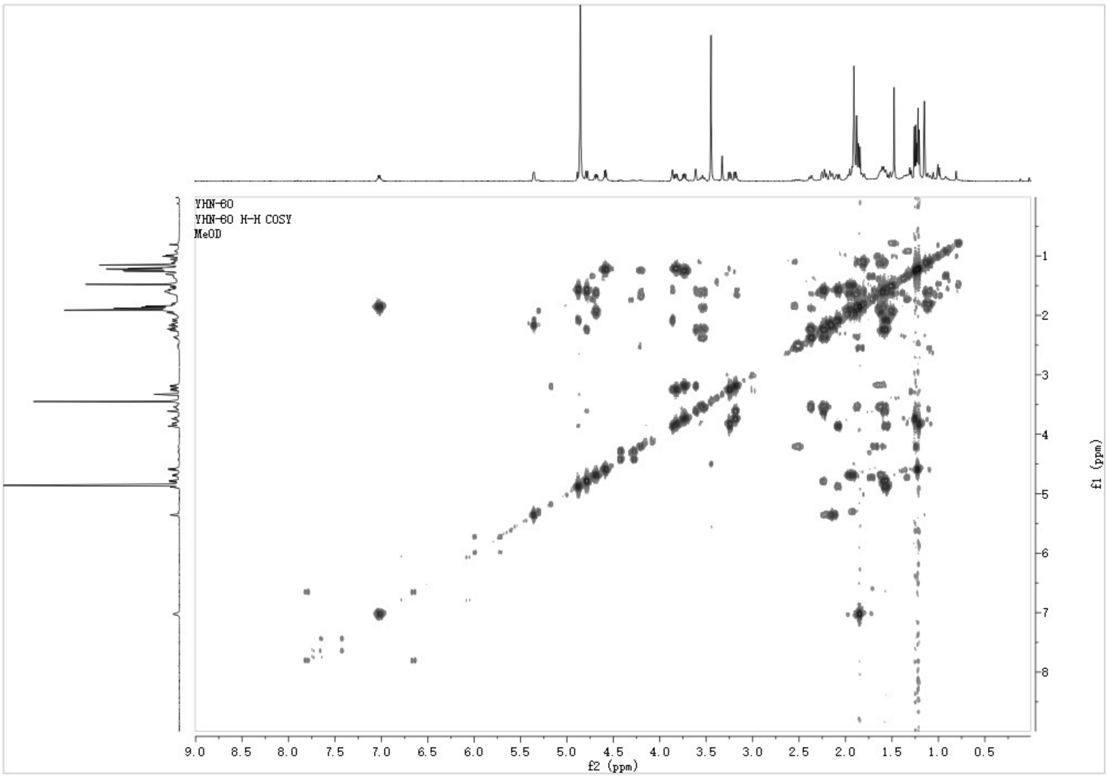

Figure S88. ^1H-^1H COSY of **13** in CD_3OD

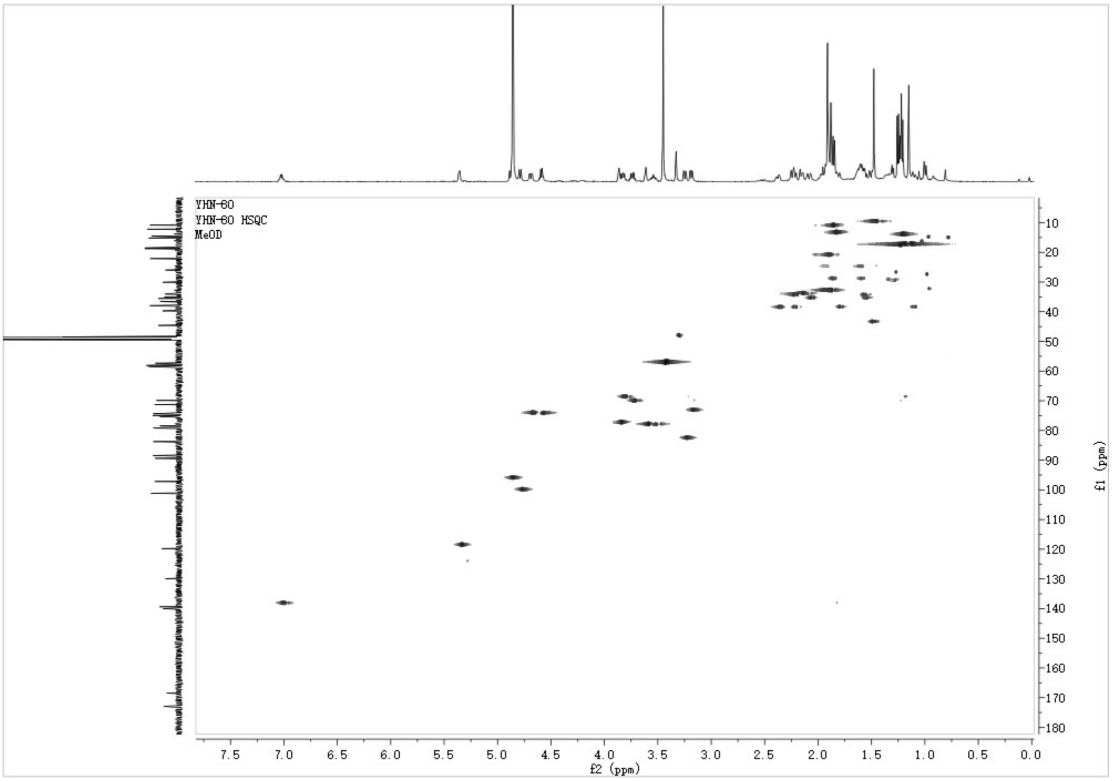

Figure S89. HSQC of **13** in CD_3OD

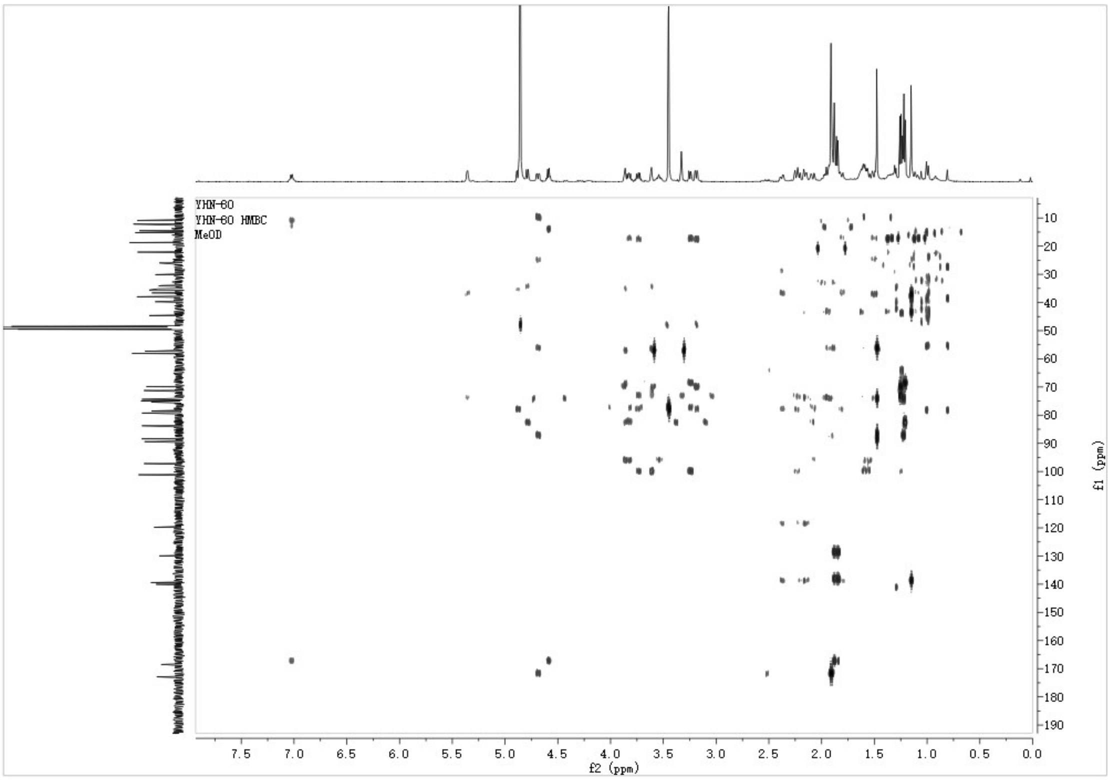

Figure S90. HMBC of **13** in CD_3OD

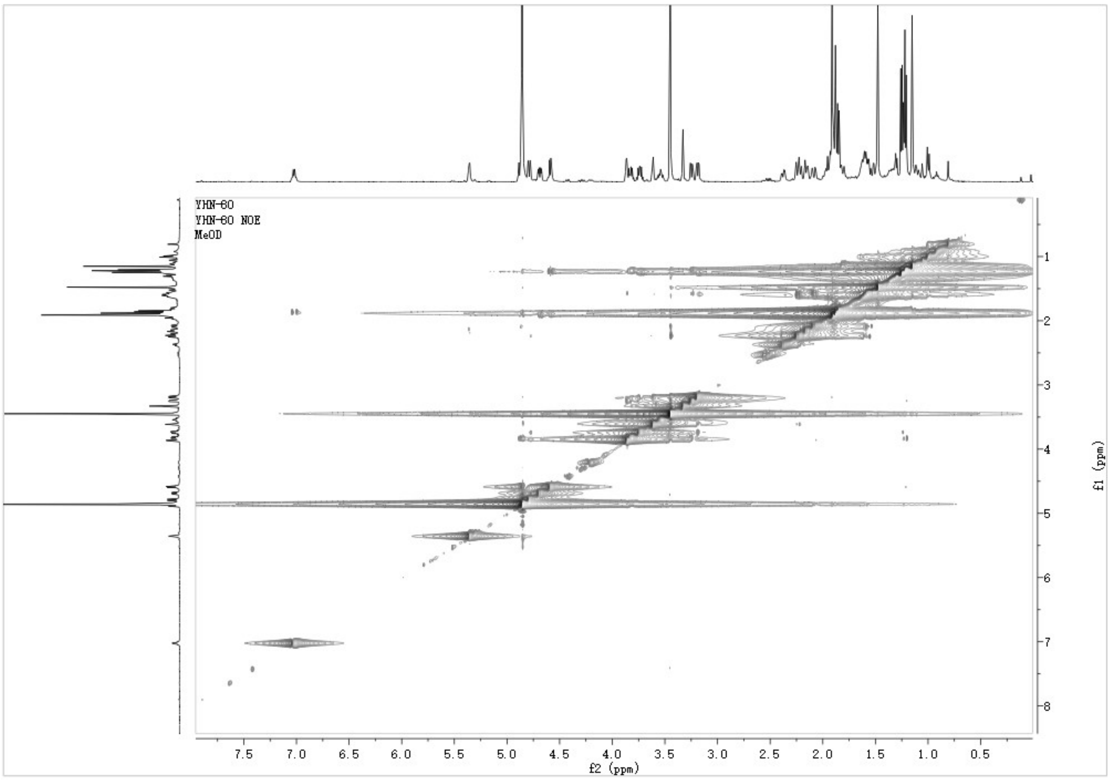

Figure S91. NOESY of **13** in CD_3OD

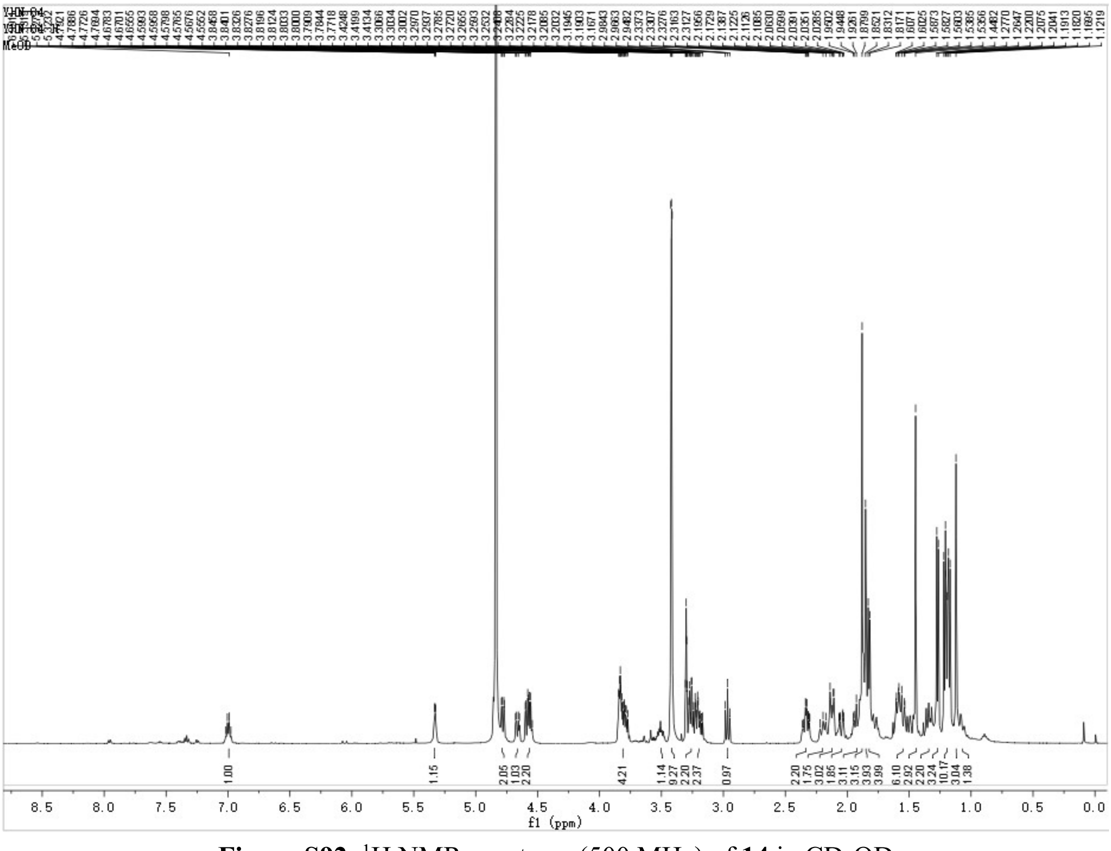

Figure S92. ^{1}H NMR spectrum (500 MHz) of **14** in CD_3OD

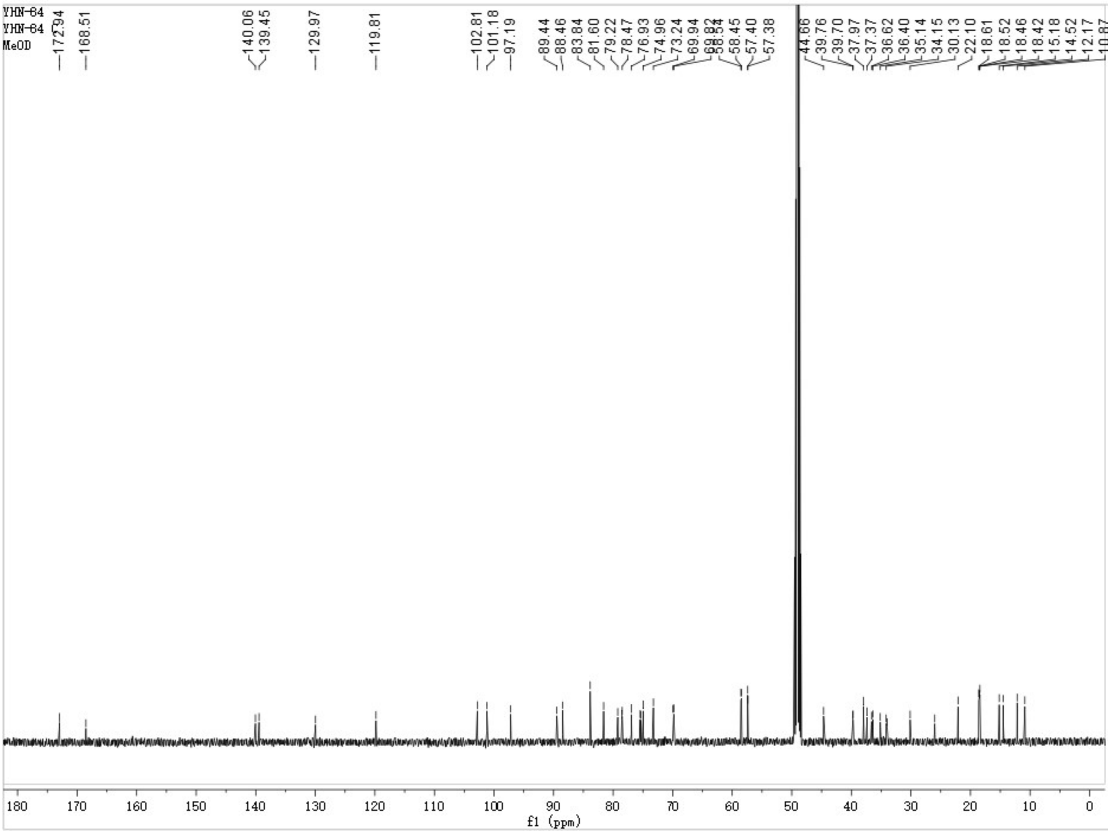

Figure S93. ^{13}C NMR spectrum (125 MHz) of **14** in CD_3OD

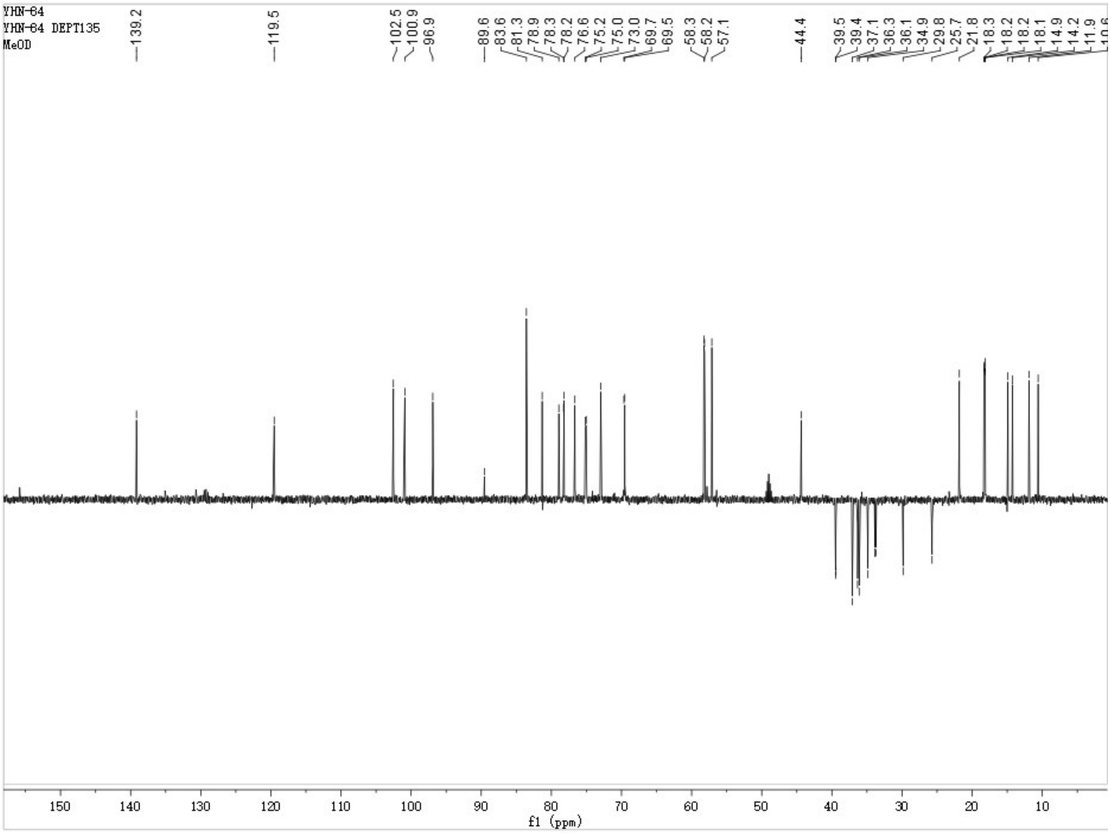

Figure S94. DEPT spectrum of **14** in CD_3OD

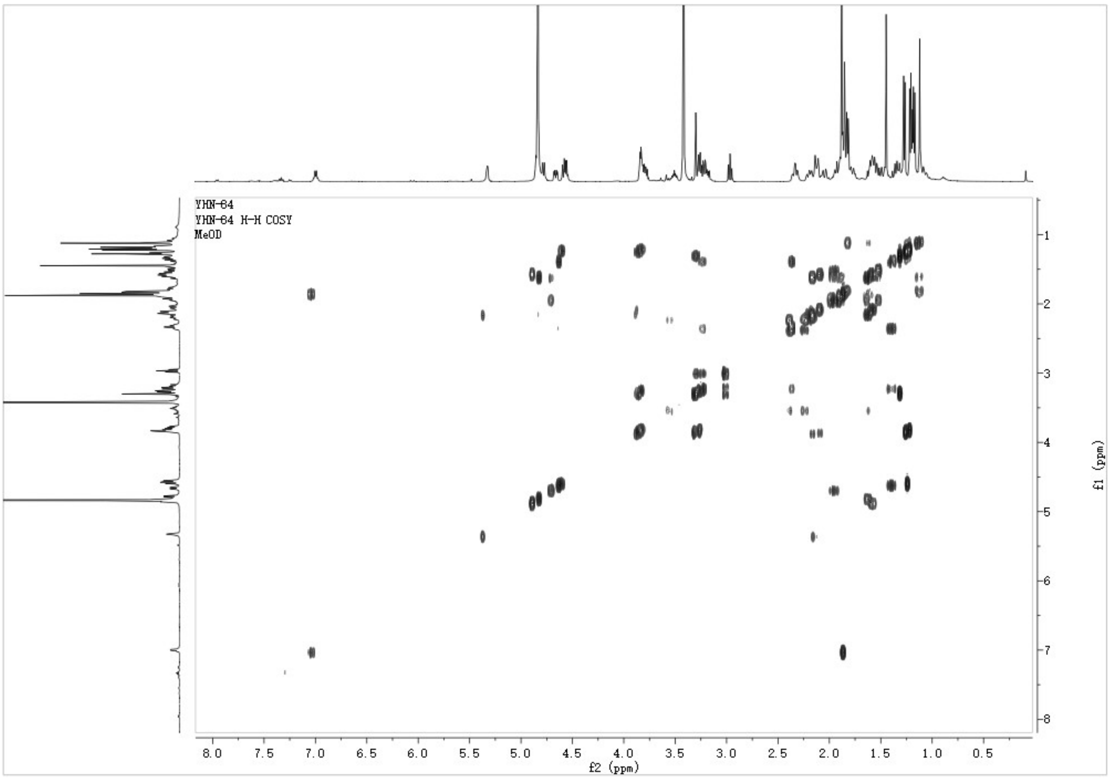

Figure S95. ^{1}H-^{1}H COSY of **14** in CD_3OD

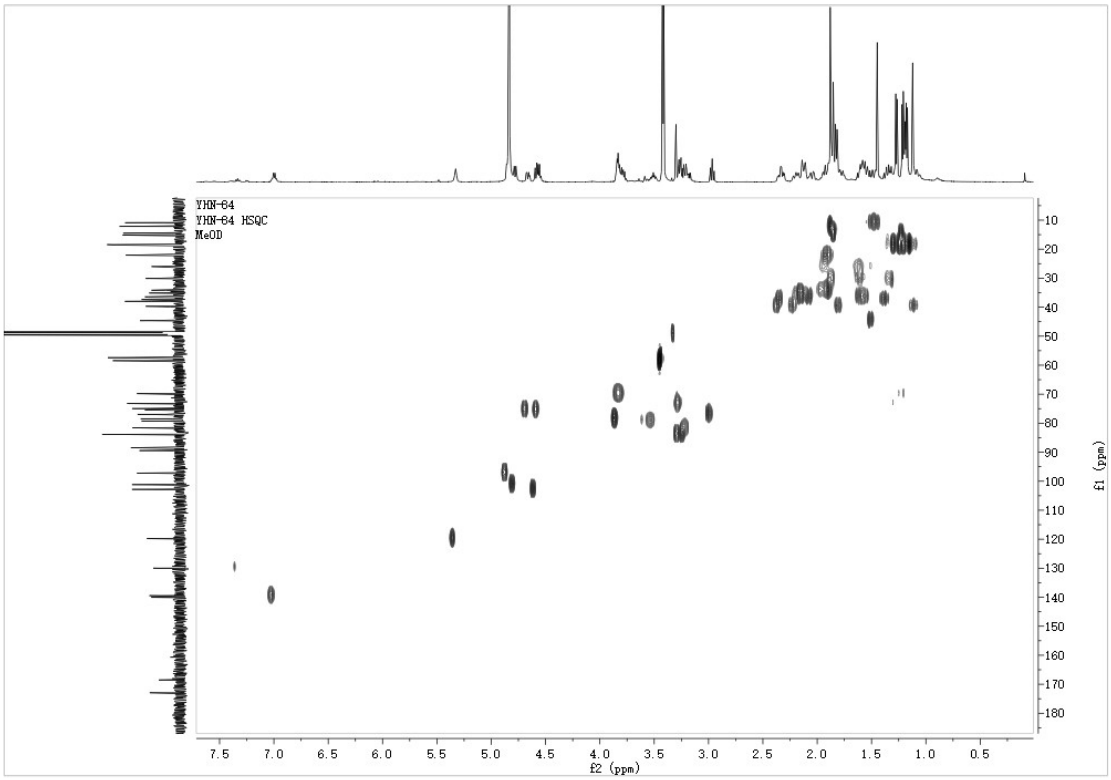

Figure S96. HSQC of **14** in CD_3OD

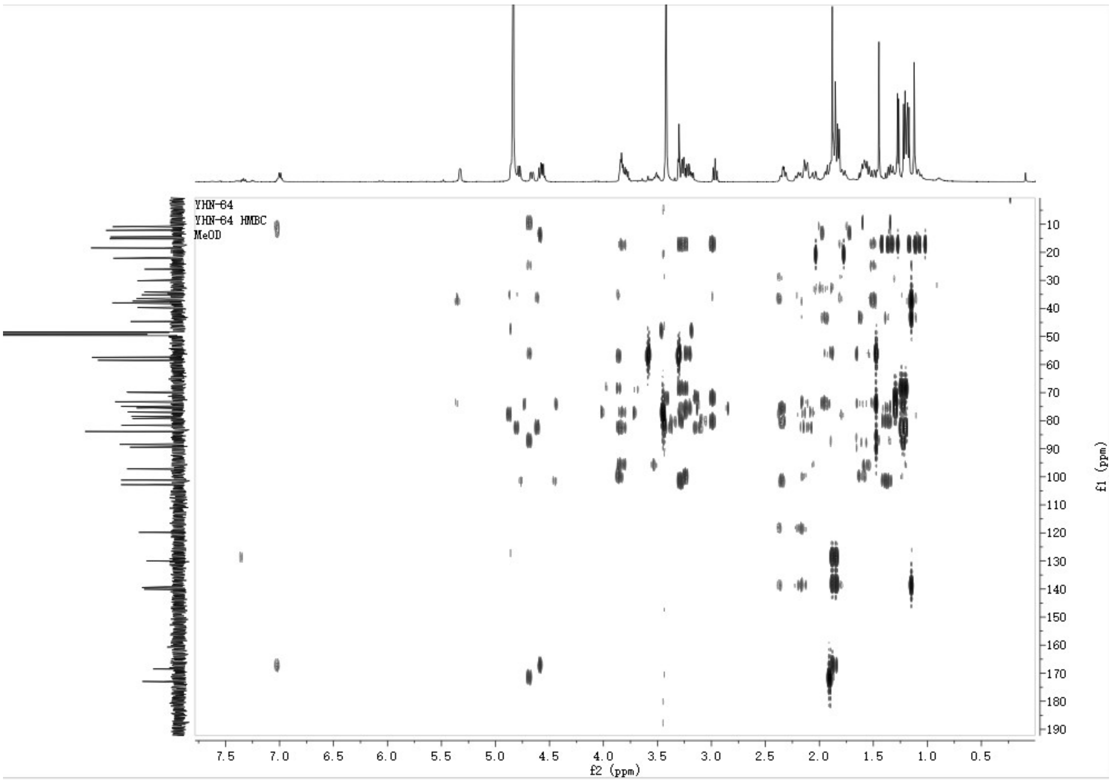

Figure S97. HMBC of **14** in CD_3OD

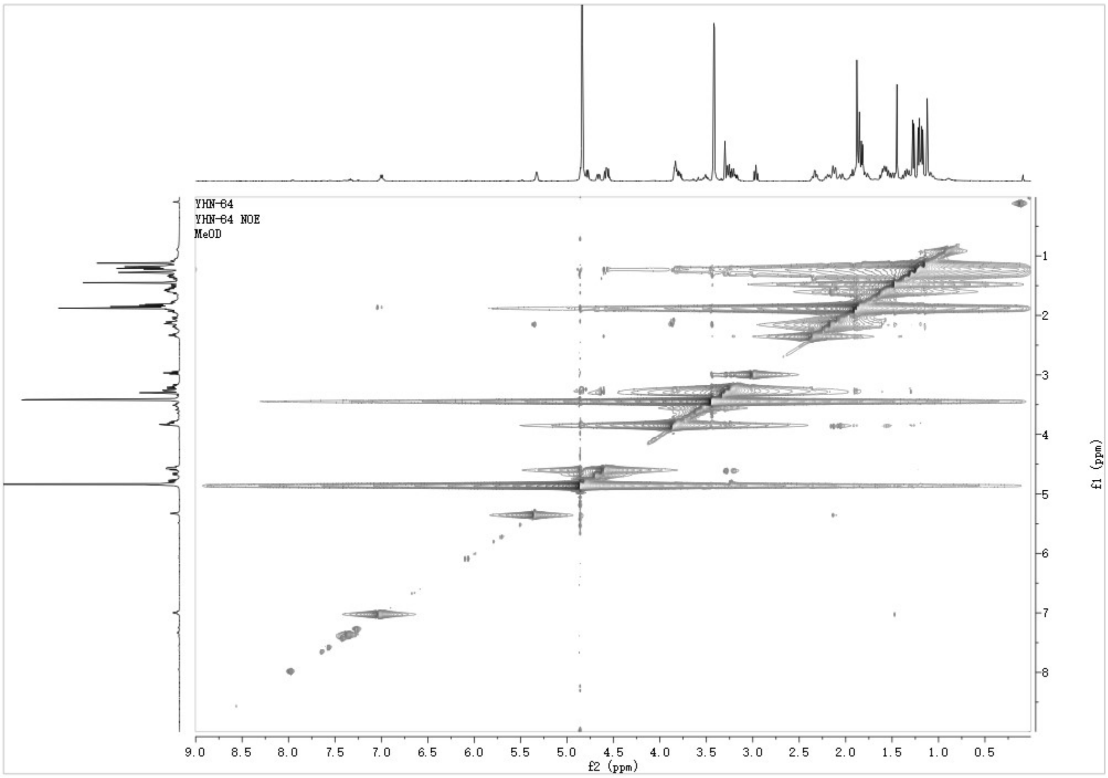

Figure S98. NOESY of **14** in CD_3OD
